# Simultaneous enhancement of photoluminescence efficiency and photostability in diphenylpyridylmethyl radicals by pentaphenylphenyl substitution

**DOI:** 10.1039/d6sc05107c

**Published:** 2026-08-11

**Authors:** Yohei Hattori, Wataru Ota, Emiko Fujiwara, Kingo Uchida, Tohru Sato, Gwénaël Rapenne

**Affiliations:** a Division of Materials Science, Nara Institute of Science and Technology 8916-5 Takayama Ikoma 630-0192 Japan hattori.yohei@naist.ac.jp; b Fukui Institute for Fundamental Chemistry, Kyoto University Takano Nishibiraki-cho 34-4, Sakyo-ku Kyoto 606-8103 Japan ota.wataru.8z@kyoto-u.ac.jp; c Department of Molecular Engineering, Graduate School of Engineering, Kyoto University Kyotodaigaku Katsura, Nishikyo-ku Kyoto 615-8510 Japan; d Materials Chemistry Course, Faculty of Advanced Science and Technology, Ryukoku University Seta Otsu Shiga 520-2194 Japan; e Université de Toulouse, CEMES-CNRS 29, Rue Marvig 31055 Toulouse France

## Abstract

Sterically demanding substituents on phenyl donor groups are required to enhance the photoluminescence efficiency of weakly emissive diphenylpyridylmethyl stable radicals by suppressing non-radiative decay. However, the resulting reduction in π-conjugation compromises photostability, leading to a trade-off between photoluminescence quantum yield (PLQY) and photostability in these systems. Here, we report pentaphenylphenyl-substituted diphenylpyridylmethyl radicals that simultaneously exhibit PLQYs exceeding 60% and photostabilities approximately 500 times greater than that of tris(2,4,6-trichlorophenyl)methyl (TTM) radical. Furthermore, peripheral methyl substitution modulates molecular polarity and alters the solvent dependence of the emission properties. Theoretical analyses reveal that pentaphenylphenyl substitution improves emission efficiency by increasing the transition dipole moment and suppressing vibronic coupling at the D_1_-optimised geometry. At the same time, charge-transfer stabilisation of the D_1_ state at the D_0_ optimised geometry increases the activation barrier for photocyclisation. These findings demonstrate that the trade-off between PLQY and photostability can be overcome by independent control of the electronic structures at the D_0_- and D_1_-optimised geometries, providing a molecular design strategy for highly efficient and photostable luminescent triarylmethyl-type radicals.

## Abstract 2

Pentaphenylphenyl-substituted diphenylpyridylmethyl radicals combine high photoluminescence quantum yields and remarkable photostability with independently controllable electronic structures at the D_0_- and D_1_-optimised geometries.

## Graphical abstract

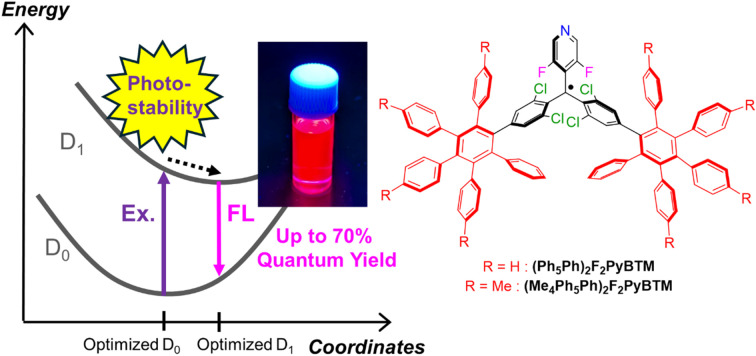

## Introduction

Recently, the chemistry of stable luminescent radicals has developed rapidly.^1–3^ These species are expected to find applications in organic light-emitting diodes,^4–6^ magnetoluminescent materials,^7–9^ and spin-optical interfaces.^10–12^ They exhibit fluorescence from the doublet lowest excited state to the doublet ground state, together with the coexistence of spin and emissive properties. Additional applications include tunable photoluminescence anisotropy amplification,^13^ fluorescent probes in heavy-atom environments,^14,15^ circularly polarized luminescent materials,^16–19^ luminescent solar concentrators,^20^ organic light-emitting transistors,^21^ near-IR imaging and phototherapy,^22,23^ combined fluorescence and magnetic resonance imaging,^24^ and X-ray scintillators.^25,26^

Triarylmethyl-type stable luminescent radicals have been known since the late 1960s;^27^ however, a major breakthrough in their development as luminescent materials was achieved in 2006 through the introduction of electron-donating substituents onto the triphenylmethyl radical framework.^28^ The photoluminescence quantum yield (PLQY) of tris(2,4,6-trichlorophenyl)methyl radical (TTM) is only a few percent; however, carbazole substitution increases the PLQY of Cz-TTM to 53% in cyclohexane. Charge-transfer processes from donor substituents to the electron-accepting radical centre have been extensively studied using triphenylamine-substituted systems.^29,30^

In 2014, we reported (3,5-dichloro-4-pyridyl)bis(2,4,6-trichlorophenyl)methyl radical (PyBTM)^31^ and demonstrated that its stability under UV irradiation was several dozen to over one hundred times higher than that of TTM^33^ in various organic solvents. At that time, high-yield photodegradation pathways of triphenylmethyl radicals had been reported,^33,34^ and we first identified photostability as a key benchmark for evaluating new stable luminescent radicals.

In 2022, we reported that the mesityl group acted as an effective donor substituent in (3,5-difluoro-4-pyridyl)bis(2,4,6-trichlorophenyl)methyl radical (F_2_PyBTM);^35^ notably, it contains only a single benzene ring as its aromatic unit.^36^ Steric hindrance from the methyl groups suppresses vibronic coupling and emission redshift, as supported by detailed density functional theory (DFT) calculations. As a result, Mes_2_F_2_PyBTM exhibited an excellent PLQY of 69% in chloroform. Subsequently, alkyl-substituted phenyl groups have been employed as luminescence-enhancing and protective substituents in triphenylmethyl radicals.^37,38^ However, Mes_2_F_2_PyBTM shows only a limited improvement in photostability compared to PyBTM (1.35-fold).

Here, we report pentaphenylphenyl-substituted F_2_PyBTM derivatives that exhibit both high PLQYs and enhanced photostability. The design concept of introducing additional phenyl groups to improve photostability was inspired by diphenylcarbazole-substituted TTM derivative (Ph2CzTTM).^39^ Building on this concept, we further introduced as many as five phenyl groups onto a single phenyl unit. Steric repulsion among these phenyl groups prevents excessive π-conjugation, thereby suppressing luminescence quenching. Furthermore, methyl substitution on the peripheral phenyl rings modulates charge separation in the excited state, thereby altering the solvent dependence of the optical properties.

## Results and discussion

**Scheme 1:**
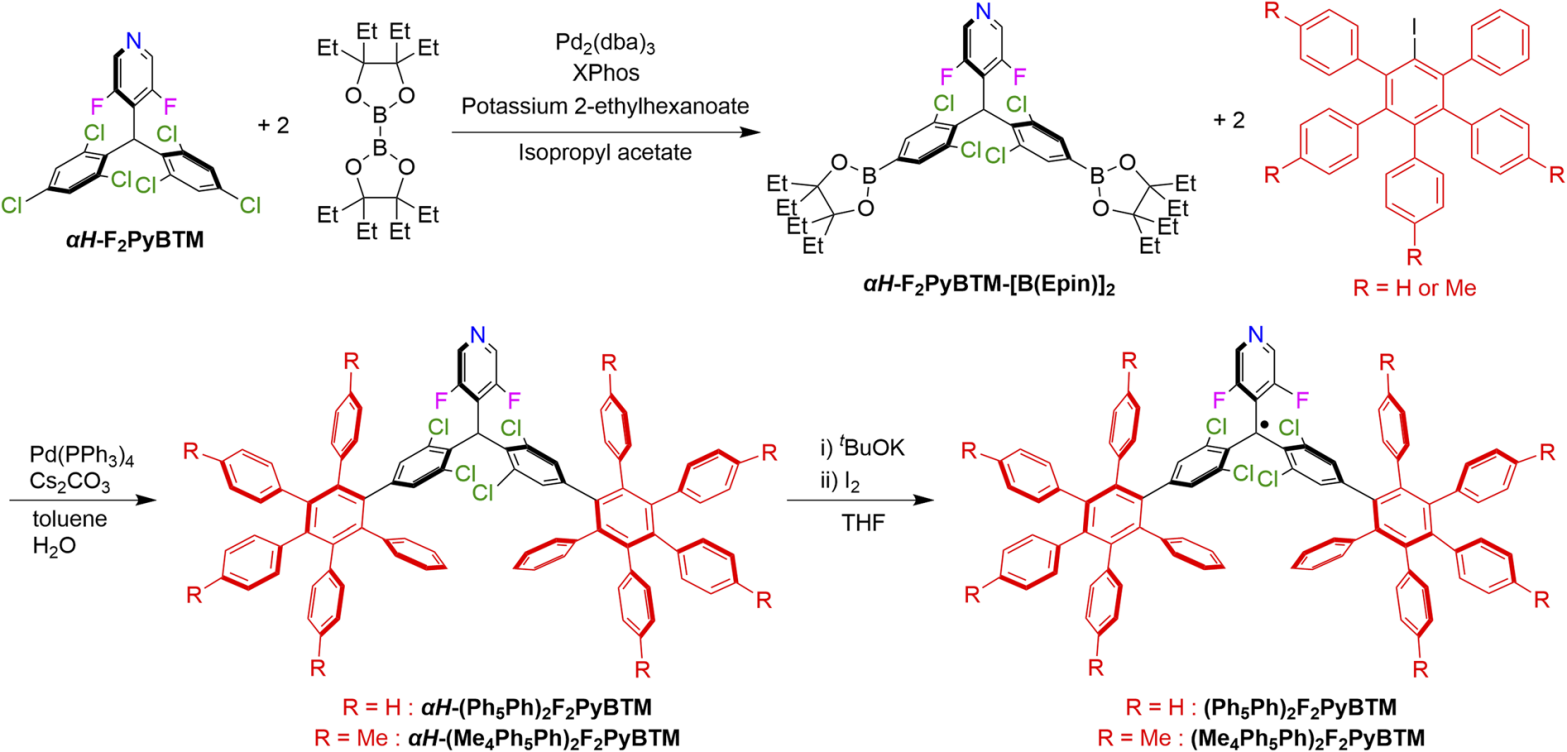
Synthetic scheme of (Ph_5_Ph)_2_F_2_PyBTM and (Me_4_Ph_5_Ph)_2_F_2_PyBTM.

The chlorine atoms at the *para*-positions of αH-F_2_PyBTM were converted into boronic ester groups to afford αH-F_2_PyBTM-[B(Epin)]_2_, using a method similar to that previously reported for the synthesis of boronic ester derivatives of αH-PyBTM (Scheme 1; see the SI for detailed synthetic procedures).^19^ Since F_2_PyBTM derivatives generally exhibit higher PLQYs than their corresponding PyBTM analogues,^35,36,40^ this new intermediate represents an important platform for the development of novel stable luminescent diphenylmethyl radicals.^41^

The diboronic ester, αH-F_2_PyBTM-[B(Epin)]_2_, was then coupled with known pentaphenylbenzene derivatives, 1-iodo-2,3,4,5,6-pentaphenylbenzene or 1-iodo-2-phenyl-3,4,5,6-(*p*-tolyl)benzene.^42^ Notably, despite the significant steric hindrance of the pentaphenylbenzene substituents, the Suzuki–Miyaura coupling reactions catalysed by Pd(PPh_3_)_4_ proceeded smoothly, affording *αH*-(Ph_5_Ph)_2_F_2_PyBTM and *αH*-(Me_4_Ph_5_Ph)_2_F_2_PyBTM in high yields.

The corresponding radicals, (Ph_5_Ph)_2_F_2_PyBTM and (Me_4_Ph_5_Ph)_2_F_2_PyBTM, were obtained by deprotonation with *t*BuOK followed by one-electron oxidation with I_2_. These radicals were characterised by HR-MS and elemental analysis. ESR (EPR) spectra recorded in toluene showed a signal at *g* = 2.003 (Fig. S1), and the presence of one *S* = 1/2 spin per molecule was confirmed by comparing the double-integrated signal intensity with that of a TEMPO reference.

**Fig. 1:**
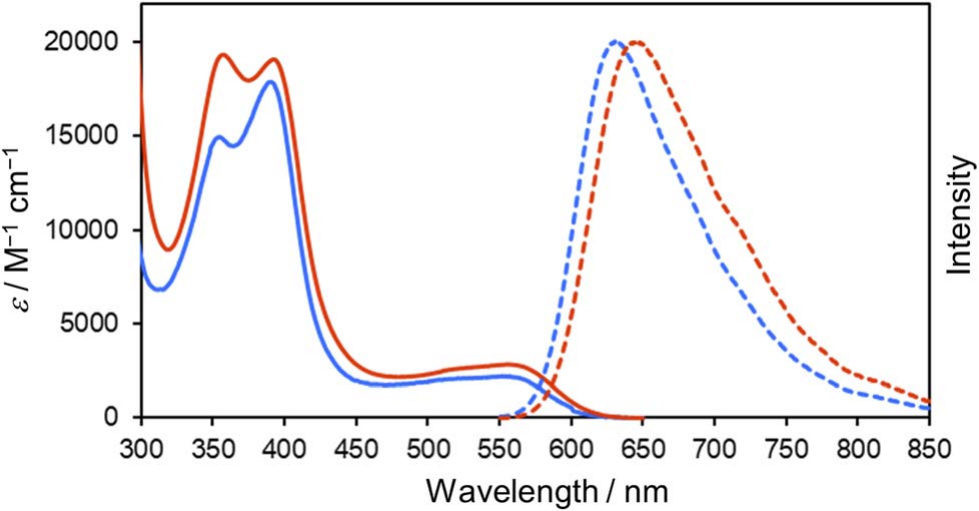
Absorption (plain lines) and emission (dashed line) spectra of (Ph_5_Ph)_2_F_2_PyBTM (blue) and (Me_4_Ph_5_Ph)_2_F_2_PyBTM (red) in chloroform.

The absorption and emission spectra of the radicals in chloroform are shown in Fig. 1. These radicals exhibit strong absorption bands in the range *λ* = 350–400 nm and relatively weak absorption in the range *λ* = 450–650 nm, similar to those observed for previously reported triarylmethyl radicals. In general, the weaker absorption in the visible region corresponds primarily to β-spin electron transitions, whereas the stronger absorption in the near-UV region mainly arises from α-spin electron transitions.

(Ph_5_Ph)_2_F_2_PyBTM shows absorption peaks at *λ* = 554, 391, and 355 nm, while (Me_4_Ph_5_Ph)_2_F_2_PyBTM shows peaks at *λ* = 555, 393, and 358 nm. The absorption intensity of (Me_4_Ph_5_Ph)_2_F_2_PyBTM is slightly higher than that of (Ph_5_Ph)_2_F_2_PyBTM. Although the absorption peaks are very similar, (Me_4_Ph_5_Ph)_2_F_2_PyBTM exhibits red-shifted emission compared to (Ph_5_Ph)_2_F_2_PyBTM. This red shift may be attributed to the enhanced donor strength of the peripheral methyl-substituted phenyl groups, which stabilises the excited state through increased charge-transfer character. The emission maxima are located at *λ* = 630 nm for (Ph_5_Ph)_2_F_2_PyBTM and at 645 nm for (Me_4_Ph_5_Ph)_2_F_2_PyBTM.

The PLQYs obtained from absolute PLQY measurements, photoluminescence lifetimes, and the calculated photophysical parameters, *Φ*_f_ = *k*_f_/(*k*_f_ + *k*_nr_) and *τ* = 1/(*k*_f_ + *k*_nr_), are summarized in Tables 1 and 2. Photoluminescence lifetimes exceeding 50 ns in cyclohexane are among the longest reported for luminescent radicals, except for through-space charge-transfer radicals.^43^ A PLQY of 70% was observed for (Ph_5_Ph)_2_F_2_PyBTM in chloroform (Fig. S2a).^36^ This value is comparable to the PLQY of 69% reported for Mes_2_F_2_PyBTM, previously the highest among diphenylpyridylmethyl radicals. (Ph_5_Ph)_2_F_2_PyBTM also exhibits a similarly high PLQY of 66% in dichloromethane (DCM), and PLQYs of approximately 50% in cyclohexane, toluene, and acetone. Higher PLQYs in halogenated solvents are attributed to larger fluorescence rate constants (*k*_f_). This suggests that the transition dipole moment of the molecule may be enhanced in solvents with comparable polarity.

**Table 1:** PLQYs and photophysical parameters of (Ph_5_Ph)_2_F_2_PyBTM

Solvent	*Φ*_f_/%	*τ*/ns	*k*_f_/10^7^ s^−1^	*k*_nr_/10^7^ s^−1^
Cyclohexane	57	53	1.1	0.8
Toluene	50	38	1.3	1.3
Chloroform	70	47	1.5	0.6
DCM	66	45	1.5	0.7
Acetone	58	44	1.3	1.0
PMMA film	82	58	1.4	0.3

**Table 2:** PLQYs and photophysical parameters of (Me_4_Ph_5_Ph)_2_F_2_PyBTM

Solvent	*Φ*_f_/%	*τ*/ns	*k*_f_/10^7^ s^−1^	*k*_nr_/10^7^ s^−1^
Cyclohexane	64	51	1.2	0.7
Toluene	64	42	1.5	0.9
Chloroform	60	37	1.6	1.1
DCM	8.3	5.9	1.4	16
Acetone	4.6	3.5	1.3	27
PMMA film	83	53	1.6	0.3

In contrast, (Me_4_Ph_5_Ph)_2_F_2_PyBTM exhibits a maximum PLQY of 64% in cyclohexane and toluene (Fig. S2b). The PLQY in chloroform is 60%, but decreases markedly to 8% in DCM and to less than 5% in acetone. A similar phenomenon has been previously observed for Ph*_2_F_2_PyBTM (Ph* = pentamethylphenyl),^44^ and fluorescence quenching in donor-radical acceptor compounds in polar solvents has been widely reported.^28,45^

These observations may be attributed to relaxation of the charge-separated D_1_ excited state *via* reorientation of polar solvent molecules. Donor-radical acceptor compounds remain highly luminescent in polar solvents only when the excited-state dipole moment is reduced through resonance effects^46^ or when the electron-donating ability of the donor is weak.^45^ Since the electron-donating ability of the pentaphenylphenyl groups is low, (Ph_5_Ph)_2_F_2_PyBTM exhibits high PLQY in all solvents. In contrast, the 2-phenyl-3,4,5,6-(*p*-tolyl)phenyl group possesses a moderate electron-donating ability due to the additional methyl groups.

PyBTM derivatives generally exhibit aggregation-induced quenching, and solid-state emission is not observed at room temperature without structural modification.^47^ However, (Ph_5_Ph)_2_F_2_PyBTM and (Me_4_Ph_5_Ph)_2_F_2_PyBTM show weak solid-state luminescence in the powder state, and their PLQYs were measured to be 0.5% and 1%, respectively. The powder emission maxima are located 629 and 634 nm, respectively (Fig. S3), and are not red-shifted relative to those in solution. It appears that the bulky pentaphenylphenyl groups weaken aggregation-induced quenching. Although the powder emission remains weak, (Ph_5_Ph)_2_F_2_PyBTM and (Me_4_Ph_5_Ph)_2_F_2_PyBTM exhibit intense emission in PMMA films with very high PLQYs (Fig. S4).

**Fig. 2:**
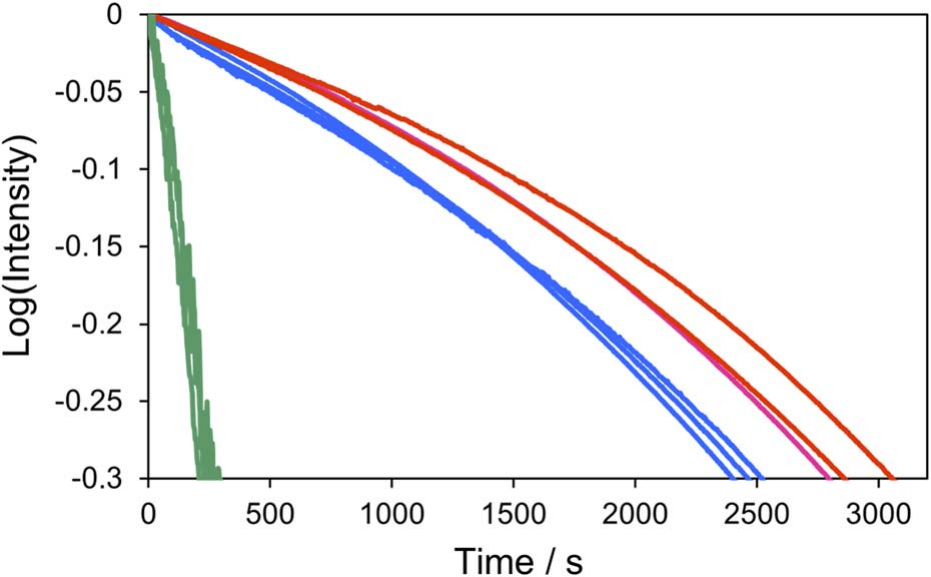
Plots showing the emission decay of (Ph_5_Ph)_2_F_2_PyBTM (blue), (Me_4_Ph_5_Ph)_2_F_2_PyBTM (red), and PyBTM (green) in cyclohexane under continuous excitation with light at *λ*_ex_ = 370 nm ± 10 nm (3 times for each radical).

The photostability of the radicals was evaluated by UV irradiation experiments in cyclohexane. In a fluorometer, (Ph_5_Ph)_2_F_2_PyBTM, (Me_4_Ph_5_Ph)_2_F_2_PyBTM, and PyBTM as a reference were irradiated with 370 nm UV light under identical conditions (Fig. 2). The average half-life of (Ph_5_Ph)_2_F_2_PyBTM is *t*_1/2_ = 2.47 × 10^3^ s, which is 10.7 times that of PyBTM (*t*_1/2_ = 2.3 × 10^2^ s). The average half-life of (Me_4_Ph_5_Ph)_2_F_2_PyBTM is slightly longer, at *t*_1/2_ = 2.91 × 10^3^ s, corresponding to 12.7 times that of PyBTM.

Considering that the photostability of PyBTM is 45 times higher than that of TTM in cyclohexane,^31,32^ the photostabilities of (Ph_5_Ph)_2_F_2_PyBTM and (Me_4_Ph_5_Ph)_2_F_2_PyBTM are estimated to be 4.8 × 10^2^ and 5.7 × 10^2^ times that of TTM, respectively. These values exceed those reported for simple donor-substituted trityl radicals such as Cz-TTM (*t*_1/2_/*t*^TTM^_1/2_ = 50),^39^ TTM-1ID (240),^48^ and PTM-3PCz (154).^49^

Although more photostable and highly luminescent radicals have recently been reported,^50–52^ improvements in photostability have generally been accompanied by decreases in PLQY in diphenylpyridylmethyl radicals.^20,44^ In general, there appears to be a trade-off between PLQY and photostability: longer photoluminescence lifetimes (*τ* = 1/(*k*_f_ + *k*_nr_)) favour high PLQYs (*Φ*_f_ = *k*_f_/(*k*_f_ + *k*_nr_) = *k*_f_ × *τ*) but can reduce photostability, as prolonged D_1_ excited-state lifetimes increase the probability of photodegradation (*t*_1/2_ ∝ 1/*τ*). In contrast, red-shifted emission enhances photostability due to higher activation barriers for photodegradation, but is often accompanied by lower PLQYs as a result of increased *k*_nr_ following the energy gap law. Despite this trade-off, the present system achieves both high PLQYs (>60%) and high photostability (*t*_1/2_/*t*^TTM^_1/2_ ≈ 500). These radicals represent the first diphenylpyridyl radicals that balance high emissive efficiency with high photostability.

Recently, theoretical and computational studies of luminescent radicals have advanced significantly. In 2020, Abdurahman *et al.* attributed the high PLQYs of donor-radical acceptor systems to intensity borrowing of the transition dipole moment and reduced non-radiative decay following the energy gap law.^51^ Cho *et al.* computed not only fluorescence (radiative decay) rate constants (*k*_f_), which are proportional to the square of transition dipole moments, but also non-radiative decay rate constants (*k*_nr_) using the Marcus–Levich–Jortner approach.^53^ In 2022, we visualized the spatial distribution of overlap densities between the D_1_ and D_0_ states and explained the increase in *k*_f_ and the decrease in *k*_nr_.^36^ In 2024, Lu *et al.* analysed structure-property relationships to achieve nearly 100% PLQY.^54^ Ghosh *et al.* investigated exciton–vibration coupling both computationally and experimentally and demonstrated how to decouple excitons from vibrational modes in organic systems.^55^

On the other hand, long-standing questions regarding the photodegradation mechanisms of triarylmethyl radicals^33,34^ have recently been resolved, and experimentally and theoretically validated pathways have been reported this year.^56,57^ We are now approaching an era in which both the PLQY and photostability of such radicals can be predicted computationally, as demonstrated in this work.

Using Gaussian 16 (Rev. C02),^58^ DFT calculations were performed at the UM06-2X/6-31G(d,p) level of theory within the Tamm–Dancoff approximation, which provides reasonable vibronic structures for donor-substituted TTM radicals.^59,60^ The solvent effect was taken into account using a polarizable continuum model (PCM).^61,62^ First, to investigate the fluorescence properties in chloroform, the D_0_ and D_1_ states of (Ph_5_Ph)_2_F_2_PyBTM and (Me_4_Ph_5_Ph)_2_F_2_PyBTM were optimised.

The torsion angles of triarylmethane moieties and the dihedral angles between benzene rings are summarized in Table S1. Although these radicals contain many single bonds, their sterically congested structures restrict conformational flexibility. All dihedral angles between two benzene rings are approximately 62° in the D_0_ states. Changes in the torsion angles of the diphenylpyridylmethyl moiety upon excitation from D_0_ to D_1_ are observed, consistent with previous studies.^36^ While most dihedral angles between benzene rings change only slightly upon excitation, the dihedral angle between one side of the dichlorophenyl ring and the substituted phenyl ring (*ϕ*_1_) decreases to 54° in the D_1_ state. The dihedral angles between the *ortho*-phenyl ring and the central phenyl ring (*ϕ*_2_ and *ϕ*_6_) also decrease to 56° or around 59°. These changes are considered to enhance π-conjugation in the charge-separated excited state, as suggested in previous studies, although the magnitude of the changes is relatively small, likely due to steric restraints.^36,44^

**Fig. 3:**
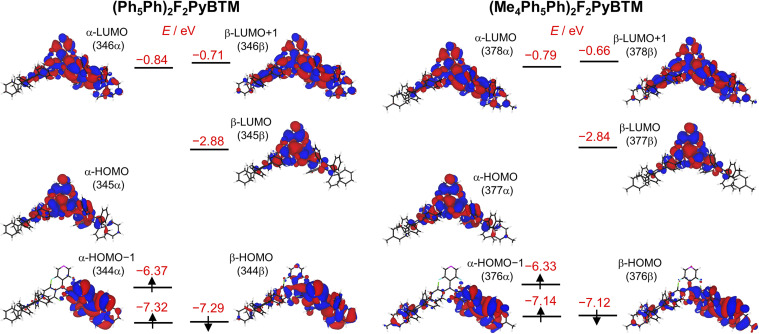
DFT-calculated frontier orbitals of (Ph_5_Ph)_2_F_2_PyBTM and (Me_4_Ph_5_Ph)_2_F_2_PyBTM in chloroform at the D_1_ optimised structure using UM06-2X/6-31G(d, p). Isosurface of molecular orbital is 1 × 10^−2^ a.u.

The frontier orbitals of (Ph_5_Ph)_2_F_2_PyBTM and (Me_4_Ph_5_Ph)_2_F_2_PyBTM at the D_1_ optimised structure are shown in Fig. 3. The α-HOMO is regarded as the so-called singly occupied molecular orbital (SOMO), while the β-LUMO is regarded as the singly unoccupied molecular orbital (SUMO). The α-HOMO (345α and 377α, respectively) and the β-LUMO (345β and 377β, respectively) are centred on the methyl radical moieties and localised on the F_2_PyBTM moieties. The α-HOMO−1 and β-LUMO of (Me_4_Ph_5_Ph)_2_F_2_PyBTM are higher than those of (Ph_5_Ph)_2_F_2_PyBTM by 0.18 and 0.17 eV, respectively.

D_1_ of (Ph_5_Ph)_2_F_2_PyBTM is mainly composed of the β-HOMO → β-LUMO transition (Table S2). Additional β-spin transitions including β-HOMO−1 → β-LUMO and β-HOMO−4 → β-LUMO, also contribute to D_1_ (Fig. S5). In (Me_4_Ph_5_Ph)_2_F_2_PyBTM, D_1_ is mainly composed of β-HOMO−4 → β-LUMO, β-HOMO → β-LUMO, and β-HOMO−1 → β-LUMO transitions. Because the β-LUMO is centred on the methyl radical moiety and delocalised over the surrounding triaryl groups, while the β-HOMO, β-HOMO−1, and β-HOMO−4 are primarily distributed over the benzene rings, the D_1_ states of these radicals are assigned to intramolecular charge-transfer (ICT) states from the hexaphenylphenyl donor to the diphenylpyridylmethyl acceptor. Therefore, these radicals can be regarded as donor-radical acceptor systems, similar to previously reported highly luminescent radicals.^36^

Within the crude adiabatic approximation,^63,64^ the rate constants of radiative and non-radiative transitions can be treated in a similar manner.^65,66^ In this approximation, the vibronic state |*Φ*_mν_(***r***,***Q***)〉, where ***r*** and ***Q*** respectively represent the electronic and mass-weighted normal coordinates, is expressed as a product of vibrational |*χ*_mν_(***Q***)〉 and electronic states |*Ψ*_m_(***r***,***Q***_0_)〉 at a fixed nuclear configuration ***Q***_0_. According to Fermi's golden rule, the rate constant of one-photon emission (mode ***k***,*λ*) from initial electronic state *m* (D_1_) to final state *n* (D_0_) is given by eqn (1) (ref. 67 and 68)1
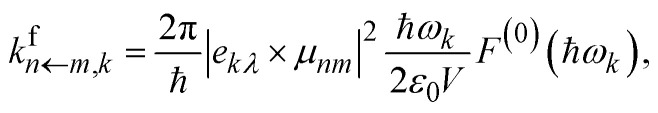
where *ħ* is the Dirac constant, ***e***_***k****λ*_ is the polarization vector, ***µ***_*nm*_ is the transition dipole moment between electronic states *m* and *n*, *ω*_***k***_ is the photon frequency, *ε*_0_ is the vacuum permittivity, and *V* is the quantisation volume. *F*^(0)^(*E*) is the Franck–Condon envelope with all vibrational modes,2
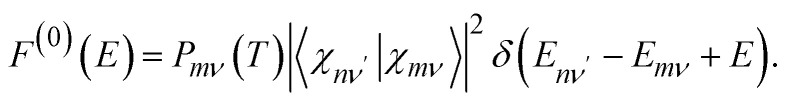
Here, *P*_*mν*_(*T*) is the Boltzmann distribution function of the initial vibrational state at temperature *T*, 
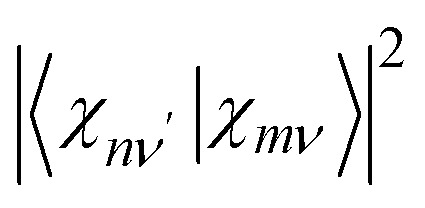
 is the Franck–Condon factor, and *E*_*mν*_ and 
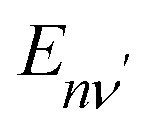
 are the initial and final vibronic energies, respectively. The rate constant for one-phonon emission (mode *α*), corresponding to internal conversion, from D_1_ to D_0_ is formulated as eqn (3) (ref. 65 and 66)3
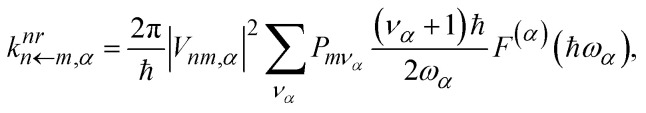
where *V*_*nm*,*α*_ is the off-diagonal vibronic coupling constant (VCC) between electronic states *m* and *n* for vibrational mode *α*, and *ν*_*α*_ is the vibrational quantum number. *F*^(*α*)^(*E*) is the Franck–Condon envelope obtained by excluding mode *α*, which is responsible for phonon emission, from the envelope that includes all vibrational modes (eqn (2)).

*k* ^r^_*n*←*m*_ and *k*^IC^_*n*←*m*_ are proportional to the squares of the transition dipole moment and the off-diagonal VCC, respectively, which can be expressed as an integral of the spatially distributed density, eqn (4) and (5).^69,70^

4 *µ*_*nm*_ = ∫d***x****τ*_*nm*_(***x***),

5 *V*_*nm*,*α*_ = ∫d***x****η*_*nm*,*α*_(***x***).

The transition dipole moment density is written as a product of a three-dimensional Cartesian coordinate (***x***) and an overlap density between electronic states *m* and *n* (*ρ*_*nm*_) as in eqn (6).

6 *τ*_*nm*_(***x***) = −*e**x**ρ*_*nm*_(***x***).

The delocalisation of the overlap density from the origin (the centre of charge) yields a large transition dipole moment. The off-diagonal vibronic coupling density (VCD) is defined by a product of the overlap density and potential derivative *ν*_*α*_ as in eqn (7).

7 *η*_*nm*,*α*_(***x***) = *ρ*_*nm*_(***x***) × *ν*_*α*_(***x***).

**Fig. 4:**
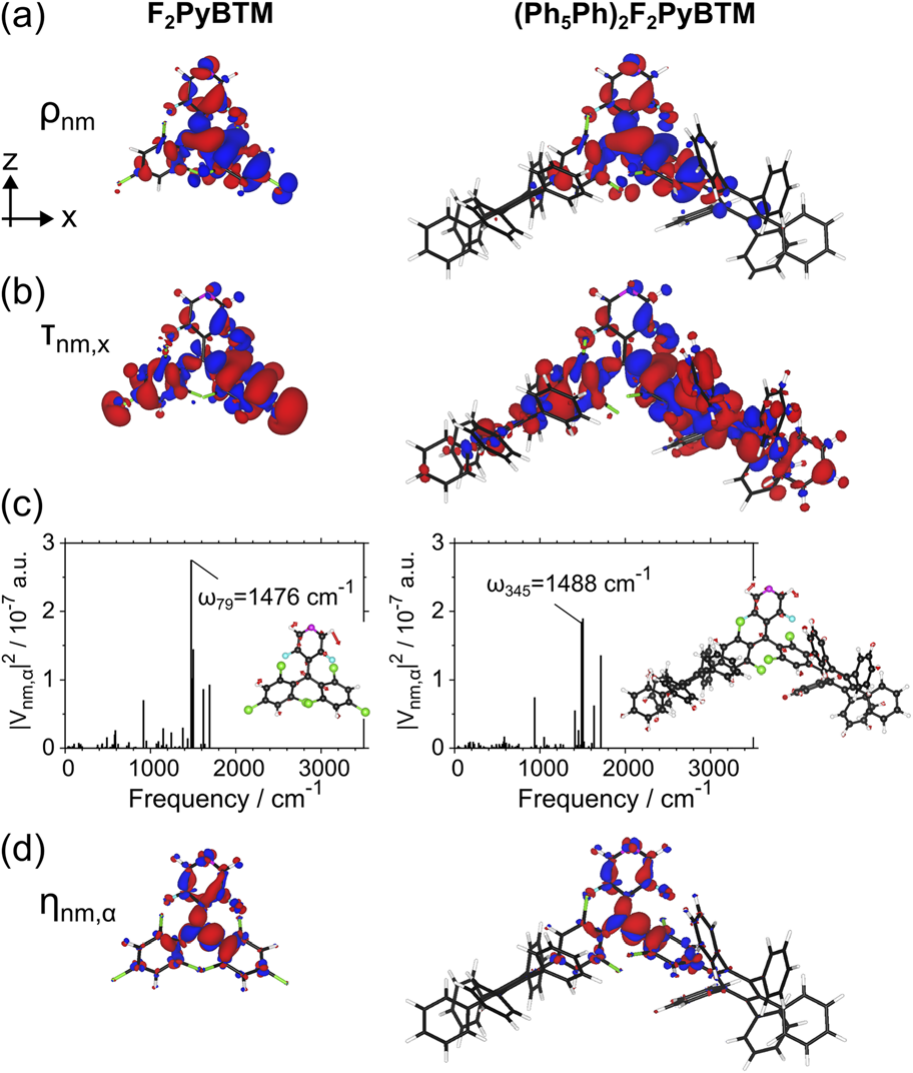
(a) Overlap density between D_1_ and D_0_ at the D_1_ optimised structure for F_2_PyBTM and (Ph_5_Ph)_2_F_2_PyBTM in chloroform. Isosurface value is 5 × 10^−4^ a.u. (b) Transition dipole moment density between D_1_ and D_0_ in the *x* component (isosurface value: 1 × 10^−3^ a.u.) at the D_1_ optimised structure in chloroform. The sign is taken so that the integral becomes positive. Red/blue region is positive/negative. Transition dipole moment in the *x* component, *µ*_*nm*,*x*_, is 0.976 a.u. for F_2_PyBTM and 1.433 a.u. for (Ph_5_Ph)_2_F_2_PyBTM. (c) Off-diagonal vibronic coupling constant between D_1_ and D_0_ at the D_1_ optimised structure in chloroform. (d) Vibronic coupling density between D_1_ and D_0_ for vibrational mode 79 in F_2_PyBTM and 345 in (Ph_5_Ph)_2_F_2_PyBTM (isosurface value: 3 × 10^−6^ a.u). The sign is taken so that the integral becomes positive. Red/blue region is positive/negative.

The distribution of the overlap density at vibrational sites increases the VCC. Therefore, the D_1_–D_0_ overlap density governs both the radiative and non-radiative rate constants.

(Ph_5_Ph)_2_F_2_PyBTM shows a fast *k*_f_ (1.4 × 10^7^ s^−1^) and a slow *k*_nr_ (0.7 × 10^7^ s^−1^) in chloroform, compared to F_2_PyBTM (*k*_f_ = 0.3 × 10^7^ s^−1^, *k*_nr_ = 5.2 × 10^7^ s^−1^). To understand this difference, the D_1_–D_0_ overlap density of F_2_PyBTM and (Ph_5_Ph)_2_F_2_PyBTM was calculated (Fig. 4a). In contrast to F_2_PyBTM, the overlap density of (Ph_5_Ph)_2_F_2_PyBTM is delocalised over the substituted pentaphenylphenyl groups. Consequently, the transition dipole moment density becomes large over the donor moiety (Fig. 4b). As a result, the D_1_–D_0_ transition dipole moment increases from |***µ***_*nm*_|^2^ = 1.27 a.u. in F_2_PyBTM to 2.45 a.u. in (Ph_5_Ph)_2_F_2_PyBTM, which explains the enhanced radiative rate constant of the donor-substituted F_2_PyBTM derivative. In addition, delocalisation of the overlap density in (Ph_5_Ph)_2_F_2_PyBTM suppresses vibronic coupling within the F_2_PyBTM moiety (Fig. 4c and d), contributing to the decreased non-radiative rate constant. Mes_2_F_2_PyBTM also exhibits delocalisation of the overlap density over the mesityl groups (Fig. S6) and therefore shows *k*_f_ and *k*_nr_ values similar to those of (Ph_5_Ph)_2_F_2_PyBTM (*k*_f_ = 1.4 × 10^7^ s^−1^, *k*_nr_ = 0.6 × 10^7^ s^−1^ in chloroform).^36^

**Scheme 2:**
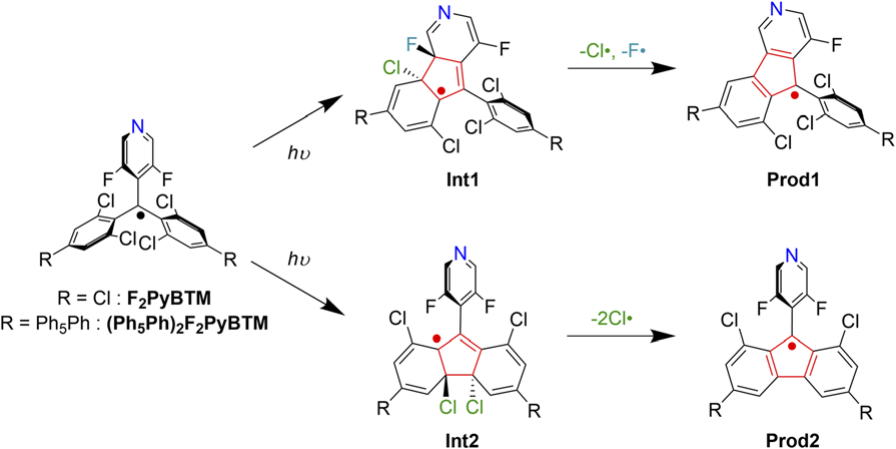
Assumed photodegradation pathways of F_2_PyBTM derivatives.

According to studies by Hackney *et al.*^56^ and Hosokai *et al.*,^57^ the photodegradation of polychlorotriarylmethyl radical derivatives begins with conrotatory photocyclisation between two aryl rings. Since the subsequent chlorine dissociation steps have much lower activation barriers, the initial photocyclisation step is rate-determining. This reaction is generally considered to proceed *via* the D_1_ excited state. Therefore, the activation barrier associated with the first transition state on the D_1_ potential energy surface was calculated. In the case of PyBTM derivatives, the photocyclisation can occur either between a chlorobenzene ring and a fluoropyridine ring or between two chlorobenzene rings (Scheme 2). Both cyclisation pathways were investigated.

**Fig. 5:**
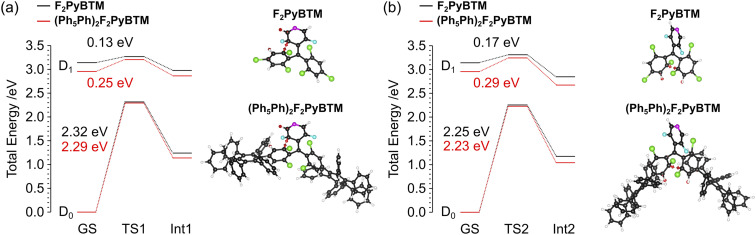
Energy diagrams for cyclisation (a) between a chlorobenzene ring and a fluoropyridine ring and (b) between two chlorobenzene rings in F_2_PyBTM (black lines) and (Ph_5_Ph)_2_F_2_PyBTM (red lines) in cyclohexane. Energy reference is the energy of D_0_@D_0_:−3704.5603 a.u. for F_2_PyBTM and −5557.0052 a.u. for (Ph_5_Ph)_2_F_2_PyBTM. Also shown is the vibrational mode with the imaginary frequency at the TS structure. The imaginary frequencies of F_2_PyBTM and (Ph_5_Ph)_2_F_2_PyBTM are 980i cm^−1^ and 993i cm^−1^, respectively, in (a), and are 784i cm^−1^ and 809i cm^−1^, respectively, in (b).

Transition states (TS) for the cyclisation reactions of F_2_PyBTM and (Ph_5_Ph)_2_F_2_PyBTM in cyclohexane were calculated as shown in Fig. 5 (see Fig. S7 for the corresponding potential energy curves along the intrinsic reaction coordinate). In the ground state (GS), the activation energies for cyclisation are greater than 2.2 eV (≈50 kcal mol^−1^), indicating that thermal cyclisation is unlikely to occur.

For F_2_PyBTM, the activation energy in the D_1_ excited state is 0.13 eV (3.0 kcal mol^−1^) for cyclisation between a chlorobenzene ring and a fluoropyridine ring and 0.17 eV (3.9 kcal mol^−1^) for cyclisation between two chlorobenzene rings. These results indicate that cyclisation between a chlorobenzene ring and a fluoropyridine ring proceeds more readily than that between two chlorobenzene rings. This trend is opposite to that reported previously for PyBTM, in which the symmetric cyclisation pathway was found to have the lower activation barrier,^55^ and may explain why F_2_PyBTM exhibits slightly lower photostability than PyBTM.^35^

For (Ph_5_Ph)_2_F_2_PyBTM, the activation energies in the D_1_ excited state were calculated to be 0.25 eV (5.8 kcal mol^−1^) for cyclisation between a chlorobenzene ring and a fluoropyridine ring, and 0.29 eV (6.7 kcal mol^−1^) for cyclisation between two chlorobenzene rings. For both pathways, the cyclisation reactions of (Ph_5_Ph)_2_F_2_PyBTM are estimated to be approximately 99 times slower than those of F_2_PyBTM. Therefore, these calculations are consistent with the experimentally observed high photostability of (Ph_5_Ph)_2_F_2_PyBTM.

The higher activation energy of (Ph_5_Ph)_2_F_2_PyBTM can be rationalized based on the electronic structures. At the GS optimised structure (Fig. S8a), the negative electron density difference of D_1_–D_0_ is delocalised over the pentaphenylphenyl groups, whereas the positive electron density difference is localised over the F_2_PyBTM moiety. This charge-transfer (CT) character of the D_0_ → D_1_ excitation stabilises the D_1_ state and leads to a longer excitation wavelength for (Ph_5_Ph)_2_F_2_PyBTM. At the TS structures (TS1, Fig. S8b, and TS2, Fig. S8c), the D_1_ state exhibits predominantly locally excited character over the F_2_PyBTM moiety, and the excited state is only weakly stabilised by the pentaphenylphenyl groups. As a result of these differences, the activation barriers of (Ph_5_Ph)_2_F_2_PyBTM are higher than those of F_2_PyBTM.

The Franck–Condon D_1_ state exhibits CT character because the D_0_ → D_1_ excitation is mainly composed of β-spin electron transitions from the pentaphenylphenyl moiety to the radical centre (Table S3). In contrast, the D_1_ states at TS1 and TS2 exhibit locally excited character because the D_0_ → D_1_ excitation is mainly composed of the α-HOMO–LUMO transition. At the transition states, the α-HOMO is destabilised (Fig. S9), and the α-HOMO–LUMO gap becomes smaller than the corresponding β-HOMO–LUMO gap.

On the other hand, the Franck–Condon D_1_ state of Mes_2_F_2_PyBTM exhibits predominantly locally excited character. In this compound, the mesityl rings are nearly perpendicular to the attached dichlorophenyl rings in the GS structure, and CT character develops only after structural relaxation to the D_1_ optimised geometry.^36^ Fragment analysis reveals that the β-spin occupied orbitals of the mesityl moiety are lower in energy than those of the pentaphenylphenyl moiety (Fig. S10). As a result, the electron density difference of D_1_–D_0_ is predominantly localised on the F_2_PyBTM moiety. Because of the limited excited-state stabilisation at the GS geometry, the activation energies in the D_1_ excited state remain low (Fig. S11). This explains why Mes_2_F_2_PyBTM exhibits photostability similar to that of F_2_PyBTM.

Proposed photodegradation products were also calculated (Fig. S12). Because these compounds exhibit low-energy D_1_ excitation (∼1000 nm) and small oscillator strengths, they are expected to be essentially non-luminescent, consistent with the photodegradation experiments. We previously found that suppressing CT-induced redshifts in the D_1_ state at the D_1_ optimised structure is important for achieving a high PLQY. In the present study, we further found that maximizing CT stabilisation in the D_1_ state at the D_0_ optimised structure is important for enhancing photostability.

Unfortunately, the actual photodegradation products of these radicals have not yet been identified experimentally. Mass spectrometry of the photodegraded residues of these radicals often gives complicated results, and this was also the case in our study (Fig. S13). This is probably why experimental identification of the photodegradation products of triphenylmethyl radicals has not been reported until this year.^56,57^ There may be multiple photodegradation pathways beyond the mechanism shown in Scheme 2. We note that even if alternative photodegradation pathways exist, the strategy of stabilising the D_1_ state should remain effective.

These results demonstrate that the trade-off between PLQY and photostability can be overcome in diphenylpyridylmethyl radicals by independently controlling the electronic structures at the D_0-_ and D_1_-optimised geometries. This design principle provides a mechanistic framework for understanding how donor substitution can simultaneously improve emission efficiency and photostability. Although its applicability to other radical frameworks remains to be established, the present findings provide a basis for the future molecular design of stable luminescent radicals.

## Conclusions

In summary, pentaphenylphenyl-substituted diphenylpyridylmethyl radicals, (Ph_5_Ph)_2_F_2_PyBTM and (Me_4_Ph_5_Ph)_2_F_2_PyBTM, were synthesised and found to exhibit both high photoluminescence quantum yields (>60%) and high photostabilities, approximately 500 times greater than that of TTM in cyclohexane. Peripheral methyl substitution modulates molecular polarity and alters the solvent dependence of the emission properties.

Computational analyses revealed that the D_1_ states of these radicals possess CT character from the hexaphenylphenyl donor to the diphenylpyridylmethyl acceptor. Delocalisation of the D_1_–D_0_ overlap density over the pentaphenylphenyl groups increases the transition dipole moment and suppresses vibronic coupling, thereby enhancing the radiative decay rate while reducing the non-radiative decay rate. In addition, calculations of photocyclisation pathways showed that the energy of the initial Franck–Condon D_1_ state is lowered by CT stabilisation, whereas the D_1_ states at the transition-state structures are only weakly stabilised because of their locally excited character. As a result, the activation barriers of (Ph_5_Ph)_2_F_2_PyBTM are higher than those of F_2_PyBTM.

The trade-off between photoluminescence quantum yield and photostability arises because molecular modifications that suppress non-radiative decay at the D_1_-optimised geometry often simultaneously weaken CT stabilisation of the Franck–Condon D_1_ state at the D_0_-optimised geometry. Independent control of the electronic structures at the D_0_- and D_1_-optimised geometries is therefore suggested as a molecular design strategy for highly efficient and photostable luminescent triarylmethyl-type radicals.

## Author contributions

Conceptualization: YH; formal analysis: WO, EF; funding acquisition: YH, WO, GR; investigation: YH; methodology: WO, TS; resources: KU; writing – original draft: YH, WO; writing – review and editing: EF, KU, TS, GR.

## Conflicts of interest

There are no conflicts to declare.

## Supplementary Material

SC-OLF-D6SC05107C-s001

## Data Availability

The data supporting this article have been included as part of the supplementary information (SI). Supplementary information is available. See DOI: https://doi.org/10.1039/d6sc05107c.
