# Supplementary material for: Simultaneous enhancement of photoluminescence efficiency and photostability in diphenylpyridylmethyl radicals by pentaphenylphenyl substitution
Source: Chem Sci. 2026 Aug 11;17(39):18675–84. doi: 10.1039/d6sc05107c (PMC13480373; doi:10.1039/d6sc05107c)

Supplementary Information (SI) for Chemical Science.
This journal is © The Royal Society of Chemistry 2026

Supplementary Information

Simultaneous Enhancement of Photoluminescence Efficiency and Photostability in Diphenylpyridylmethyl Radicals by Pentaphenylphenyl Substitution

Yohei Hattori,*[a] Wataru Ota,[b,c] Emiko Fujiwara,[b] Kingo Uchida,[d] Tohru Sato,[b,c] and Gwénaël Rapenne [a,e]

[a] Nara Institute of Science and Technology, 8916–5 Takayama, Ikoma, 630–0192, Japan

[b] Fukui Institute for Fundamental Chemistry, Kyoto University, Takano Nishibiraki-cho 34-4, Sakyo-ku, Kyoto 606-8103, Japan

[c] Department of Molecular Engineering, Graduate School of Engineering, Kyoto University, Kyotodaigaku Katsura, Nishikyo-ku, Kyoto 615-8510, Japan

[d] Materials Chemistry Course, Faculty of Advanced Science and Technology, Ryukoku University, Seta, Otsu, Shiga 520-2194, Japan

[e] Université de Toulouse, CEMES-CNRS, 29, rue Marvig, 31055 Toulouse, France

Content

I. Experimental section **2**

Fig. S1 Temperature-dependent ESR spectra of the powder sample 6

Fig. S2 Absolute PLQY and lifetime in solution 6

Fig. S3 Emission spectra in powder state 7

Fig. S4 Absolute PLQY and lifetime in powder state 7

II. Cartesian coordinates of all the optimized geometries by DFT calculation 8

Table S1 Calculated torsion angles of the aryl rings of the radicals in chloroform. 12

Table S2 D_1 excited states at the D_1 optimized structure in chloroform. 12

Fig. S5 Orbital levels and molecular orbitals at the D_1 optimized structure in chloroform. 13

Fig. S6 Overlap density, transition dipole moment density, and vibronic coupling of Mes_2F_2PyBTM. 14

Fig. S7 Potential energy curves along intrinsic reaction coordinate for cyclisation . 14

Fig. S8 Electron density difference of D_1–D_0. 15

Table S3 D_1 excited states at the ground state and transition state structures in cyclohexane. 15

Fig. S9 Orbital levels at the ground state and transition state structures in cyclohexane. 16

Fig. S10 Density of states and its projection onto radical and donor fragment in cyclohexane. 16

Fig. S11 D_1-D_0 electron density difference and energy diagrams for cyclisation in Mes_2F_2PyBTM. 17

Fig. S12 D_1 excitation energy and D_1-D_0 electron density difference of the expected photodegradation products. 17

Fig. S13 Mass spectrometry of photodegradation products. 18

III. NMR spectra 19

IV. HR-MS spectra 23

EXPERIMENTAL SECTION

All reactions were carried out under an argon atmosphere. The starting materials, 1-iodo-2,3,4,5,6-pentaphenylbenzene and 1-iodo-2-phenyl-3,4,5,6-tetra(*p*-tolyl)benzene[42] were prepared according to literature procedures. Commercially available compounds were used without further purification. Preparative recycling gel permeation chromatography was performed with a recycling preparative HPLC, LaboACE LC-5060, Japan Analytical Industry Co., Ltd. ^{1}H (400 MHz) spectra were recorded on a JEOL JNM-ECX400P spectrometer, ^{1}H (600 MHz) and $^{13}C\{^{1}H\}$ NMR (151 MHz) spectra were recorded on a JEOL JNM-ECA 600 spectrometer, and ^{19}F NMR (470 MHz) spectra were recorded on a JEOL JNM-ECZ 500R spectrometer using $CDCl_3$. The residual solvent signals (^{1}H NMR: $\delta = 7.26$ or ^{13}C NMR: $\delta = 77.16$) and hexafluorobenzene signals (^{19}F NMR: $\delta = -164.9$) were used as the internal standards. ESR (EPR) spectra were recorded with a JEOL JES-FA100N spectrometer using X-band microwaves. Sample solutions were placed in a 2.5mmϕ sample tube. Magnetic field was calibrated with the Mn^{2+}/MgO standard. Absorption and emission spectra were recorded on a Hitachi U-3310 spectrophotometer and a Hitachi F-7100 fluorescence spectrophotometer, respectively. Photostability under 370 nm light was recorded with a JASCO FP-8600KS spectrofluorometer. Absolute luminescence quantum yields and fluorescence lifetimes were measured using Hamamatsu Photonics Quantaurus QY C11347 and Quantaurus Tau C11367, respectively. Elemental analysis of $(Me_4Ph_5Ph)_2F_2$PyBTM was conducted at Center for Organic Elemental Microanalysis, Graduate School of Pharmaceutical Sciences, Kyoto University. The elemental analyses were performed using YANACO MT-6 at the Center for Organic Elemental Microanalysis, Graduate School and Faculty of Pharmaceutical Sciences, Kyoto University. High-resolution mass spectrometry was conducted using a JEOL JMS S-3000 MALDI-TOF MS spectrometer. DFT calculations were performed using Gaussian 16 Rev. C.02.

Synthesis of *αH*-F_2PyBTM-[B(Epin)]$_2$

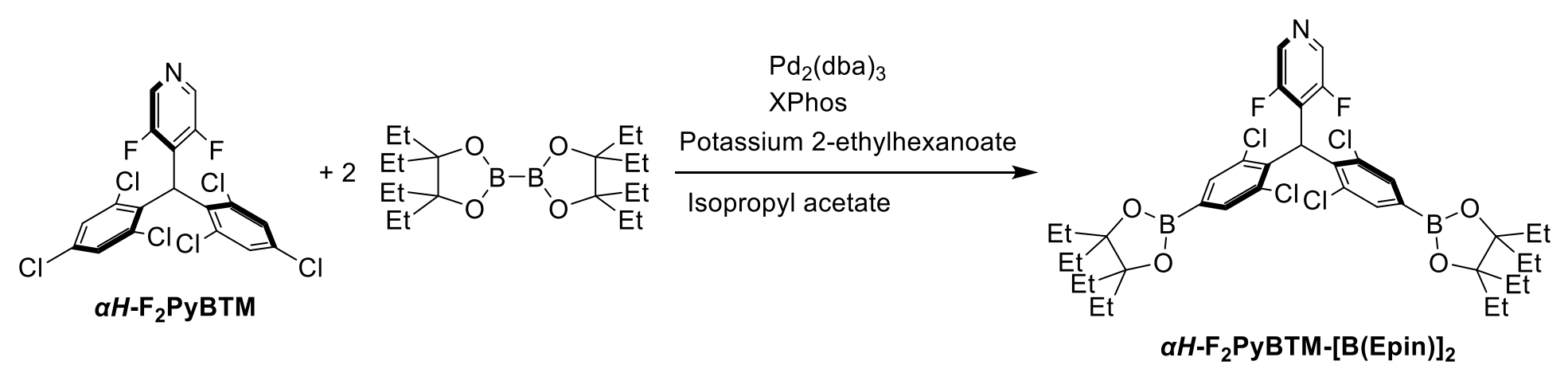

In a 100 mL eggplant flask, a mixture of *αH*-F_2PyBTM (979 mg, 2.01 mmol), $B_2(Epin)_2$ (1.65 g, 4.51 mmol), $Pd_2(dba)_3$ (45.9 mg, 0.0501 mmol), XPhos (48.6 mg, 0.102 mmol), potassium 2-ethylhexanoate (1.92 g, 10.5 mmol), and isopropyl acetate (20 mL) was stirred at 75 °C overnight under an argon atmosphere. After cooling to room temperature, the reaction mixture was diluted with $CHCl_3$ (75 mL), washed with water (50 mL), and the aqueous layer was subsequently extracted with $CHCl_3$ (10 mL × 2). The combined organic layer was washed with water (50 mL), dried over Na_2SO_4, filtered, and concentrated in vacuo. The residue was purified by column chromatography on silica gel ($CHCl_3$) and then GPC (eluent: $CHCl_3$) to give *αH*-F_2PyBTM-[B(Epin)]$_2$ (572 mg, 0.730 mmol, 36%) as a cream-colored solid.

^{1}H NMR (400 MHz, $CDCl_3$, ppm) $\delta = 8.30$ (s, 1H), 8.17 (s, 1H), 7.67 (s, 4H), 6.75 (s, 1H), 1.82–1.66 (m, 16H), 0.95 (t, 18H, $J = 7.4$ Hz).

^{13}C NMR (151 MHz, $CDCl_3$, ppm): δ = 160.0, 158.6, 158.3, 156.9, 136.4, 136.3, 136.2, 135.3, 135.2, 135.0, 133.6, 133.4, 131.2, 125.0, 124.9, 124.8, 89.8, 89.7, 89.3, 42.9, 26.5, 8.9.

^{19}F NMR (470 MHz, $CDCl_3$, ppm): δ = –128.4, –130.8.

HRMS (MALDI-TOF MS positive mode) m/z: $[MH]^+$ calcd for $C_{38}H_{48}B_2F_2NO_4$ 784.2457; found 784.2473.

Synthesis of *αH*-($Me_4Ph_5Ph)_2F_2$PyBTM

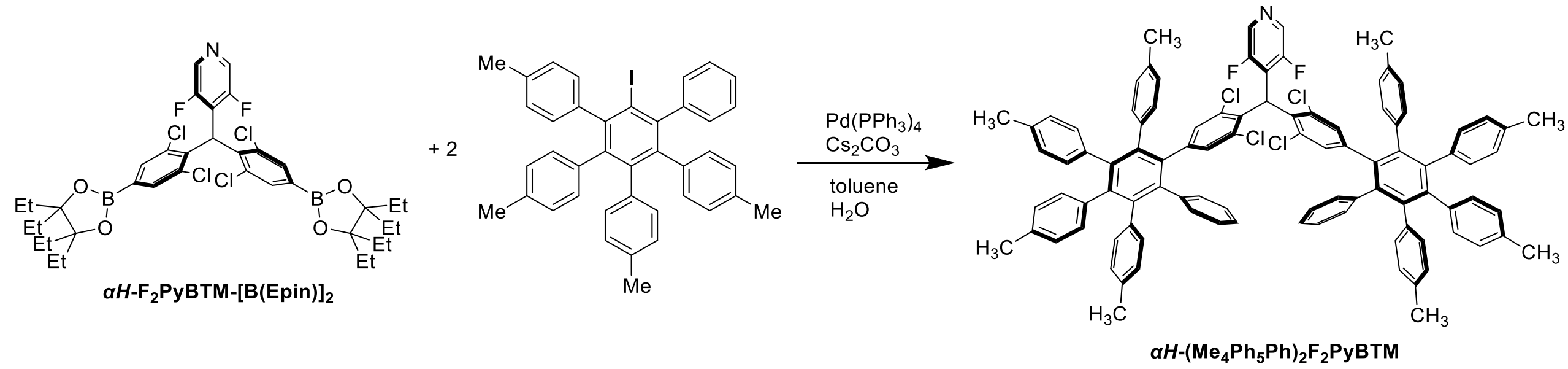

In a Schlenk tube, 1-iodo-2-phenyl-3,4,5,6-tetra(*p*-tolyl)benzene (193 mg, 0.302 mmol), *αH*-F_2PyBTM-$[B(Epin)]_2$ (77.2 mg, 0.0986 mmol), tetrakis(triphenylphosphine)palladium(0) (55.0 mg, 0.0476 mmol), and dry toluene (1.5 mL) were refluxed. Degassed Cs_2CO_3 aq. (326 mg, 1.00 mmol in 0.5 mL) was added and the mixture was heated at 97 °C for 3 days. After cooling to room temperature, water was added to the reaction mixture, and the mixture was extracted with $CHCl_3$. The combined organic layer was concentrated in vacuo. The residue was purified by column chromatography on silica gel ($CHCl_3$: hexane = 1: 1) and then GPC (eluent: $CHCl_3$) to give *αH*-($Me_4Ph_5Ph)_2F_2$PyBTM (114.3 mg, 0.0791 mmol, 80.2%) as a reddish white solid.

^1H NMR (400 MHz, $CDCl_3$, ppm): δ = 8.17 (s, 1H), 8.03 (s, 1H), 6.89 (br, 6H), 6.78 (br, 4H), 6.72-6.62 (m, 32H), 6.59 (s, 4H), 6.11 (s, 1H), 2.14-2.08 (m, 24H).

^{13}C NMR (151 MHz, $CDCl_3$, ppm): δ =142.6, 141.4, 140.5, 140.3, 140.3, 140.1, 140.1, 137.6, 137.6, 137.4, 137.4, 137.0, 135.0, 134.6, 134.6, 134.5, 132.1, 132.0, 131.3, 131.3, 131.1, 130.2, 127.7, 127.5, 127.0, 125.6, 41.8, 21.2, 21.2, 21.2.

^{19}F NMR (470 MHz, $CDCl_3$, ppm): δ = –128.7, –131.3.

HRMS (MALDI-TOF MS positive mode) m/z: $[M]^+$ calcd for $C_{98}H_{73}NF_2Cl_4$ 1441.4460; found 1441.4454.

Synthesis of ($Me_4Ph_5Ph)_2F_2$PyBTM

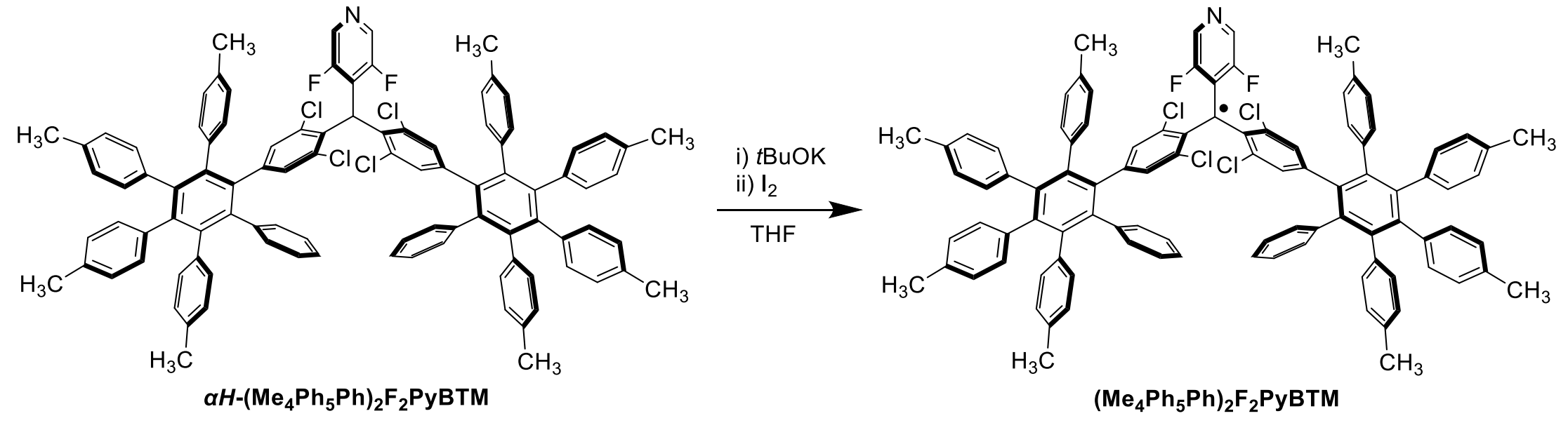

In a Schlenk tube, αH-($Me_4Ph_5Ph)_2F_2$PyBTM (23.0 mg, 0.0159 mmol) was dissolved in dry THF (2 mL) and *t*BuOK in THF (1 M solution, 0.5 mL) was added under an argon atmosphere. The reaction mixture was stirred for 25 min, and a solution of iodine (9.8 mg, 0.039 mmol) in dry THF (0.5 mL) was then added, and the mixture was stirred for 35 min. The excess iodine was reduced by adding $Na_2S_2O_3$ (16 mg, 0.064 mmol) in water (0.5 mL). The reaction mixture was dried over Na_2SO_4, filtered, and concentrated in vacuo. The residue was purified by column chromatography on silica gel ($CHCl_3$: hexane = 1: 1 → 3: 2), and dried in vacuo to give ($Me_4Ph_5Ph)_2F_2$PyBTM (19.5 mg, 0.0135 mmol, 85%) as a red solid.

Elemental analysis calcd for $C_{98}H_{72}NF_2Cl_4$: C 81.55, H 5.03, N 0.97, found: C 81.40, H 4.99, N 0.96.

HRMS (MALDI-TOF MS positive mode) m/z: $[MH]^+$ calcd for $C_{98}H_{73}NF_2Cl_4$ 1441.4460; found 1441.4478.

Synthesis of αH-($Ph_5Ph)_2F_2$PyBTM

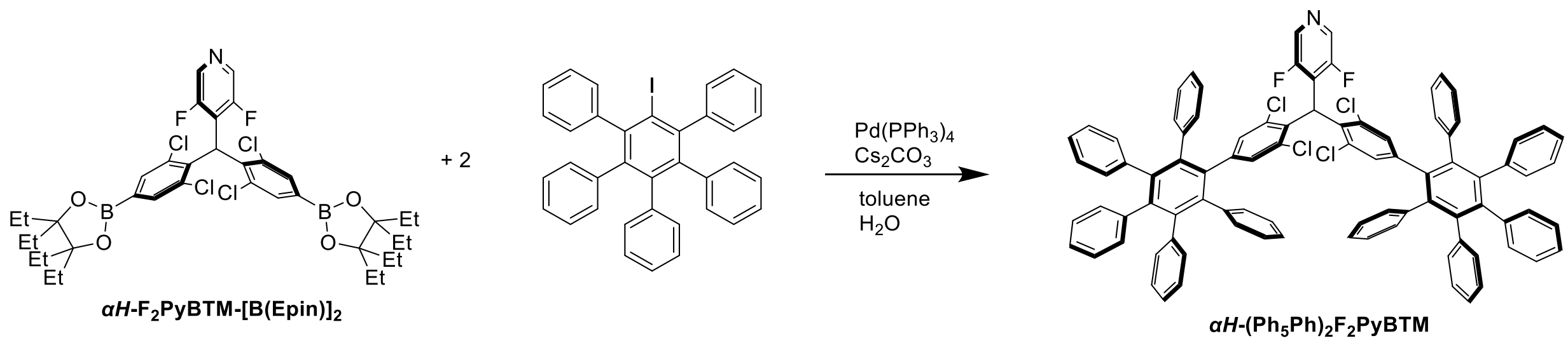

In a Schlenk tube, 1-iodo-2,3,4,5,6-pentaphenylbenzene (176.7 mg, 0.302 mmol), αH-F_2PyBTM-[B(Epin)]$_2$ (77.6 mg, 0.0990 mmol), tetrakis(triphenylphosphine)palladium(0) (63.2 mg, 0.0547 mmol), and dry toluene (4.7 mL) were refluxed. Degassed Cs_2CO_3 aq. (326 mg, 1.00 mmol in 0.5 mL) was added, and the mixture was heated at 95 °C for 3 days. After cooling to room temperature, the reaction mixture was dried over Na_2SO_4. $CHCl_3$ and toluene were added to the solution, which was then filtered through a celite pad and concentrated in vacuo. The residue was purified by column chromatography on silica gel ($CHCl_3$), GPC (eluent: $CHCl_3$), and then reprecipitated from $CHCl_3$-MeOH to give αH-($Ph_5Ph)_2F_2$PyBTM (92.2 mg, 0.0692 mmol, 70%) as a white solid.

^{1}H NMR (600 MHz, $CDCl_3$, ppm): δ = 8.18 (s, 1H), 8.05 (s, 1H), 6.92-6.79 (m, 50H), 6.64 (s, 4H), 6.11 (s, 1H).

^{13}C NMR (151MHz, $CDCl_3$, ppm): δ =159.8, 158.4, 158.1, 156.7, 142.2, 141.3, 140.5, 140.4, 140.4, 140.3, 139.8, 137.4, 134.9, 134.7, 133.3, 133.1, 132.1, 131.4, 131.4, 131.3, 130.4, 127.1, 126.8, 126.8, 125.8, 125.6, 125.5, 125.3, 125.2, 125.1, 41.8.

^{19}F NMR (470 MHz, $CDCl_3$, ppm): δ = –128.8, –131.2.

HRMS (MALDI-TOF MS positive mode) m/z: $[MH]^+$ calcd for $C_{90}H_{58}NF_2Cl_4$ 1330.3286; found 1330.3304.

Synthesis of ($Ph_5Ph)_2F_2$PyBTM

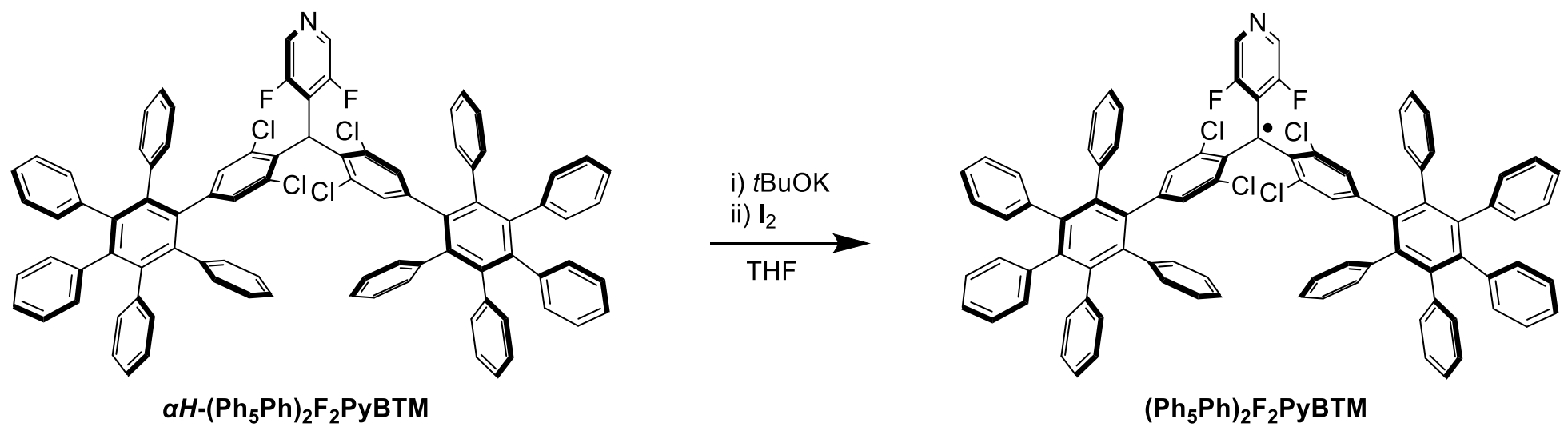

In a Schlenk tube, *αH*-$(Ph_5Ph)_2F_2$PyBTM (28.0 mg, 0.0210 mmol) was dissolved in dry THF (3 mL) and *t*BuOK in THF (1 M solution, 0.5 mL) was added under an argon atmosphere. The reaction mixture was stirred for 20 min, and a solution of iodine (14.7 mg, 0.0579 mmol) in dry THF (0.6 mL) was then added, and the mixture was stirred for 50 min. The excess iodine was reduced by adding $Na_2S_2O_3$ (16 mg, 0.064 mmol) in water (0.5 mL). The reaction mixture was dried over Na_2SO_4, filtered, and concentrated in vacuo. The residue was purified by column chromatography on silica gel ($CHCl_3$: hexane = 1: 1), GPC (eluent: $CHCl_3$), and dried in vacuo to give $(Ph_5Ph)_2F_2$PyBTM (23.8 mg, 0.0179 mmol, 85%) as a red solid.

Elemental analysis calcd for $C_{90}H_{56}NF_2Cl_4$: C 81.20, H 4.24, N 1.05, found: C 81.00, H 4.31, N 0.87.

HRMS (MALDI-TOF MS positive mode) m/z: $[MH]^+$ calcd for $C_{90}H_{57}NF_2Cl_4$ 1329.3208; found 1329.3208.

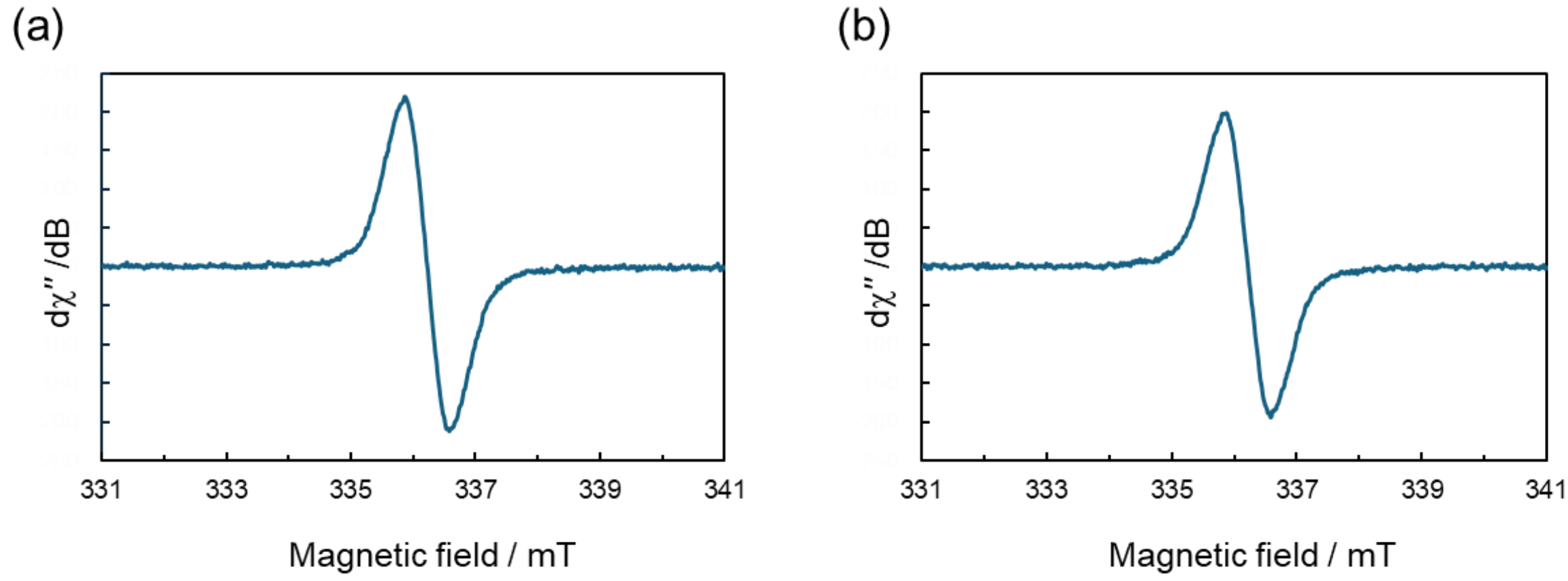

Fig. S1 ESR spectra of (a) $(Me_4Ph_5Ph)_2F_2PyBTM$ and (b) $(Ph_5Ph)_2F_2PyBTM$. The *g* factors of the radicals were *g* = 2.003.

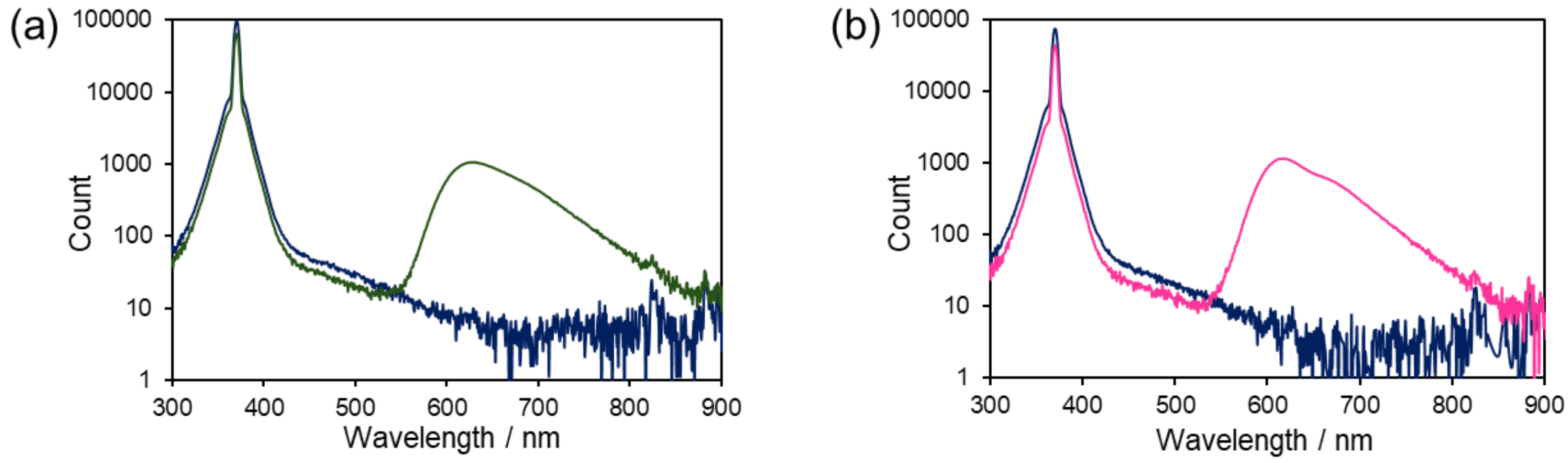

	359.68 - 380.89(nm)	545.02 – 874.58(nm)	PLQY
Blank	256503641	---	---
$(Ph_5Ph)_2$ F_2PyBTM	172990868	58540758	0.701

	359.68 - 380.89(nm)	541.27 - 875.31(nm)	PLQY
Blank	202624548	---	---
$(Me_4Ph_5Ph)_2$ F_2PyBTM	117522922	54094868	0.636

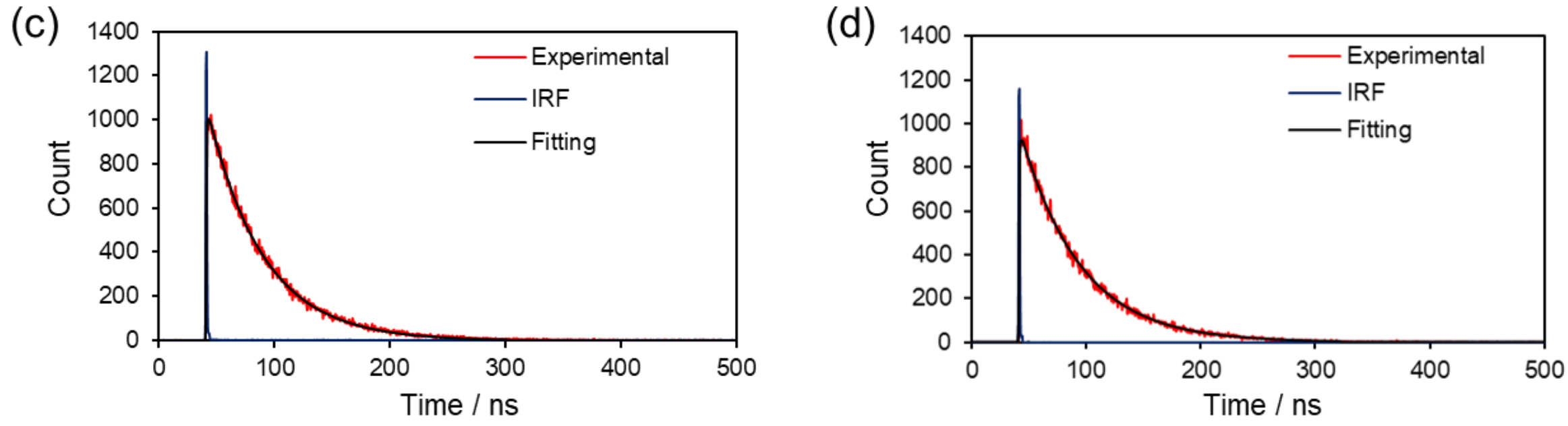

Fig. S2 (a) Absolute PLQY measurement of $(Ph_5Ph)_2F_2PyBTM$ in chloroform. (b) Absolute PLQY measurement of $(Me_4Ph_5Ph)_2F_2PyBTM$ in cyclohexane. (c) Photoluminescence decay curve of $(Ph_5Ph)_2F_2PyBTM$ in chloroform excited at λ_{ex} = 365 nm. (d) Photoluminescence decay curve of $(Me_4Ph_5Ph)_2F_2PyBTM$ in cyclohexane excited at λ_{ex} = 365 nm.

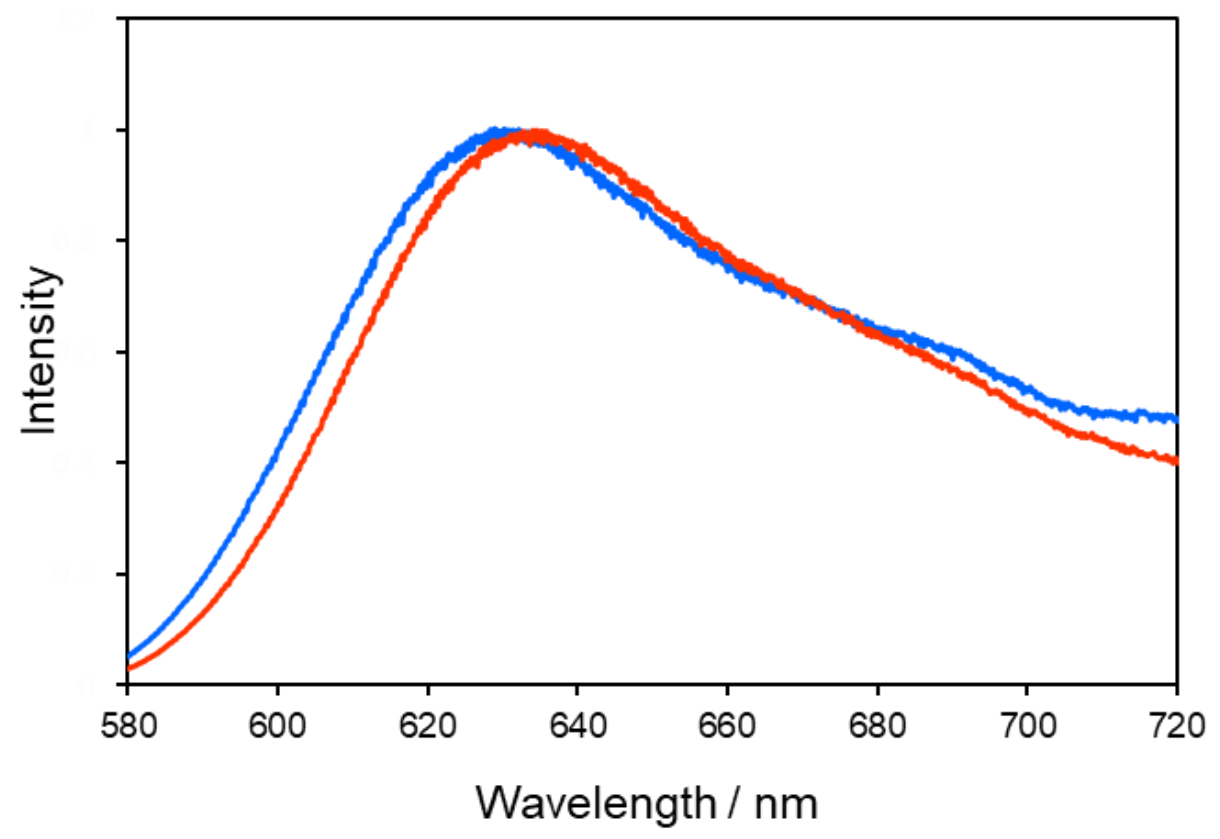

Fig. S3 Emission (dashed line) spectra of $(Ph_5Ph)_2F_2PyBTM$ (blue) and $(Me_4Ph_5Ph)_2F_2PyBTM$ (red) in the powder state excited at λ_{ex} = 370 nm.

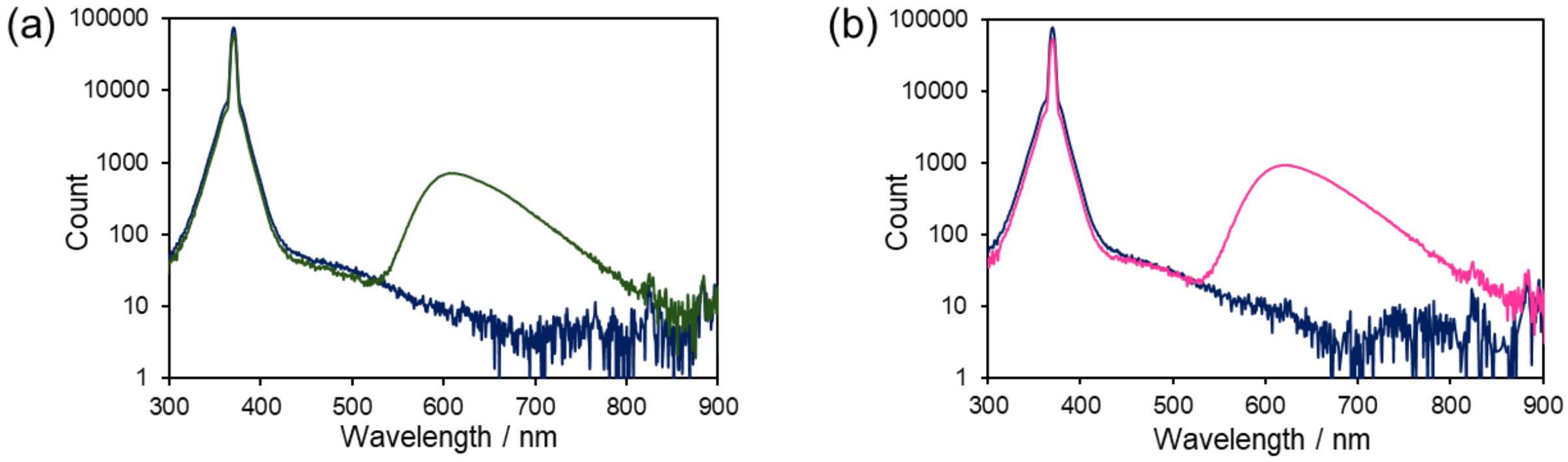

	359.68 - 380.89(nm)	528.54 – 819.79(nm)	PLQY
Blank	201177040	---	---
$(Ph_5Ph)_2$ F_2PyBTM	156023202	37043702	0.820

	359.68 - 380.89(nm)	530.79 - 874.58(nm)	PLQY
Blank	208733532	---	---
$(Me_4Ph_5Ph)_2$ F_2PyBTM	146225696	51881792	0.830

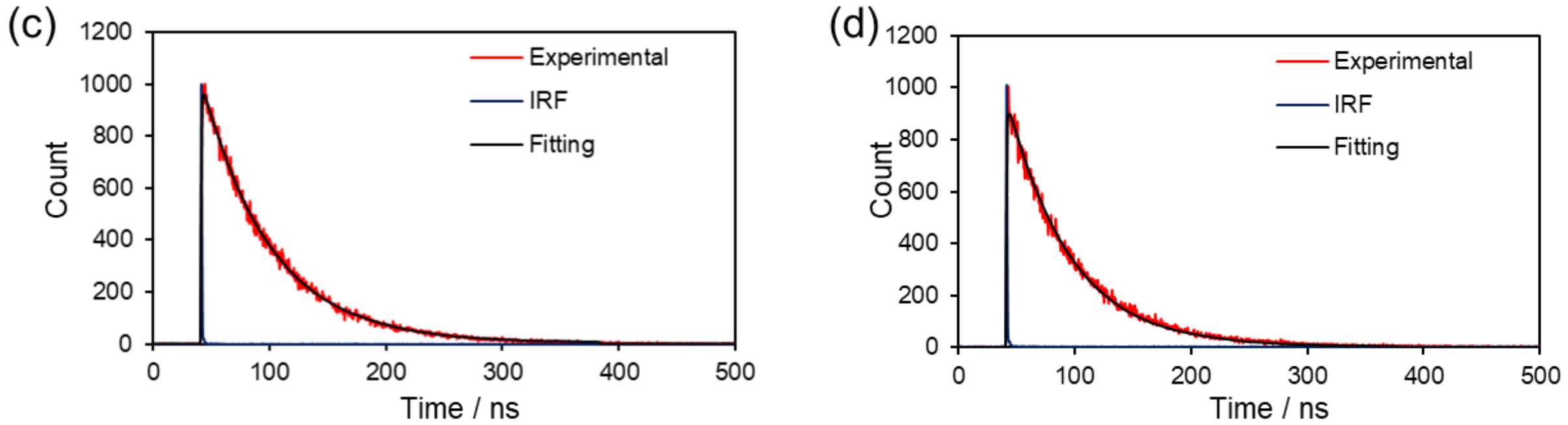

Fig. S4 (a) Absolute PLQY measurement of $(Ph_5Ph)_2F_2PyBTM$ in PMMA film. (b) Absolute PLQY measurement of $(Me_4Ph_5Ph)_2F_2PyBTM$ in PMMA film. (c) Photoluminescence decay curve of $(Ph_5Ph)_2F_2PyBTM$ in PMMA film excited at λ_{ex} = 365 nm. (d) Photoluminescence decay curve of $(Me_4Ph_5Ph)_2F_2PyBTM$ in PMMA film excited at λ_{ex} = 365 nm.

Cartesian coordinates of the optimized geometries by DFT calculations (UM06-2X/6-31G(d,p)) in chloroform

$(Ph_5Ph)_2F_2PyBTM$ (D_0 state)

Standard orientation:

Center Number	Atomic Number	Atomic Type	Coordinates (Angstroms) X	Y	Z
1	6	0	-1.180536	6.621026	-1.584701
2	6	0	-1.176796	5.386143	-0.912247
3	6	0	1.055622	5.867122	-0.044962
4	6	0	-0.058986	5.014674	-0.145219
5	6	0	-0.058071	3.712631	0.585667
6	6	0	-1.001428	3.465594	1.583545
7	6	0	0.895421	2.737056	0.305146
8	1	0	-1.769649	4.198427	1.804202
9	6	0	-0.971431	2.275243	2.287571
10	6	0	0.910177	1.549756	1.022163
11	1	0	1.651904	2.912004	-0.451856
12	17	0	-2.238596	2.024072	3.460891
13	6	0	-0.015890	1.268507	2.043563
14	17	0	2.189412	0.422143	0.648126
15	6	0	0.000000	0.000000	2.789102
16	6	0	0.000000	0.000000	4.235782
17	6	0	0.015890	-1.268507	2.043563
18	6	0	0.525059	1.047827	5.013006
19	6	0	-0.525059	-1.047827	5.013006
20	6	0	0.971431	-2.275243	2.287571
21	6	0	-0.910177	-1.549756	1.022163
22	6	0	0.511819	1.019480	6.397107
23	6	0	-0.511819	-1.019480	6.397107
24	17	0	2.238596	-2.024072	3.460891
25	6	0	1.001428	-3.465594	1.583545
26	17	0	-2.189412	-0.422143	0.648126
27	6	0	-0.895421	-2.737056	0.305146
28	7	0	0.000000	0.000000	7.088242
29	1	0	0.937394	1.854244	6.945667
30	1	0	-0.937394	-1.854244	6.945667
31	6	0	0.058071	-3.712631	0.585667
32	1	0	1.769649	-4.198427	1.804202
33	1	0	-1.651904	-2.912004	-0.451856
34	6	0	0.058986	-5.014674	-0.145219
35	6	0	1.176796	-5.386143	-0.912247
36	6	0	-1.055622	-5.867122	-0.044962
37	6	0	1.180536	-6.621026	-1.584701
38	6	0	0.067479	-7.477204	-1.488175
39	6	0	-0.067479	7.477204	-1.488175
40	6	0	-1.050776	-7.100227	-0.720682
41	6	0	1.050776	7.100227	-0.720682
42	6	0	2.235201	5.444436	0.771468
43	6	0	2.096190	5.184350	2.138446
44	6	0	3.482717	5.254451	0.170114
45	6	0	3.176652	4.726806	2.886169
46	1	0	1.128827	5.325628	2.612523
47	6	0	4.564667	4.798178	0.917374
48	1	0	3.599761	5.462676	-0.889694
49	6	0	4.413538	4.529321	2.276087
50	1	0	3.048313	4.515721	3.942952
51	1	0	5.525680	4.647544	0.436233
52	1	0	5.255575	4.166438	2.856409
53	6	0	2.235700	8.006394	-0.623170
54	6	0	2.632337	8.526229	0.612198
55	6	0	2.966795	8.343066	-1.766095
56	6	0	3.735903	9.369318	0.702951
57	1	0	2.072594	8.261485	1.504928
58	6	0	4.074279	9.180766	-1.675493
59	1	0	2.658768	7.947457	-2.729876
60	6	0	4.460536	9.697756	-0.440678
61	1	0	4.030556	9.768837	1.668047
62	1	0	4.634936	9.431130	-2.570390
63	6	0	-0.076041	8.792731	-2.198921
64	6	0	-0.164532	8.847144	-3.592790
65	6	0	0.000000	9.987812	-1.477873
66	6	0	-0.174923	10.072005	-4.253210
67	1	0	-0.231589	7.921696	-4.157882
68	6	0	-0.016817	11.213350	-2.136867
69	1	0	0.076721	9.951308	-0.394797
70	6	0	-0.102848	11.258592	-3.526692
71	1	0	-0.240918	10.099438	-5.336150
72	1	0	0.038779	12.133585	-1.564215
73	6	0	-2.369888	7.020465	-2.397596
74	6	0	-2.756975	6.262200	-3.506101
75	6	0	-3.113194	8.155118	-2.060040
76	6	0	-3.862765	6.633158	-4.265365
77	1	0	-2.187191	5.375193	-3.768704
78	6	0	-4.223083	8.523101	-2.814497
79	1	0	-2.812541	8.752404	-1.203811
80	6	0	-4.599659	7.764029	-3.920522
81	1	0	-4.149703	6.037453	-5.125989
82	1	0	-4.793235	9.404524	-2.539038
83	6	0	-2.354385	4.468444	-0.994212
84	6	0	-3.598902	4.866052	-0.495896
85	6	0	-2.220492	3.187877	-1.538404
86	6	0	-4.684047	3.995692	-0.529318
87	1	0	-3.710839	5.863032	-0.078958
88	6	0	-3.307966	2.319841	-1.580754
89	1	0	-1.255340	2.870580	-1.923767
90	6	0	-4.541224	2.719589	-1.070694
91	1	0	-5.641943	4.314073	-0.130909
92	1	0	-3.189758	1.329261	-2.008330
93	6	0	2.354385	-4.468444	-0.994212
94	6	0	3.598902	-4.866052	-0.495896
95	6	0	2.220492	-3.187877	-1.538404
96	6	0	4.684047	-3.995692	-0.529318
97	1	0	3.710839	-5.863032	-0.078958
98	6	0	3.307966	-2.319841	-1.580754
99	1	0	1.255340	-2.870580	-1.923767
100	6	0	4.541224	-2.719589	-1.070694
101	1	0	5.641943	-4.314073	-0.130909
102	1	0	3.189758	-1.329261	-2.008330
103	6	0	2.369888	-7.020465	-2.397596
104	6	0	2.756975	-6.262200	-3.506101
105	6	0	3.113194	-8.155118	-2.060040
106	6	0	3.862765	-6.633158	-4.265365
107	1	0	2.187191	-5.375193	-3.768704
108	6	0	4.223083	-8.523101	-2.814497
109	1	0	2.812541	-8.752404	-1.203811
110	6	0	4.599659	-7.764029	-3.920522
111	1	0	4.149703	-6.037453	-5.125989
112	1	0	4.793235	-9.404524	-2.539038
113	6	0	0.076041	-8.792731	-2.198921
114	6	0	-0.000000	-9.987812	-1.477873
115	6	0	0.164532	-8.847144	-3.592790
116	6	0	0.016817	-11.213350	-2.136867
117	1	0	-0.076721	-9.951308	-0.394797
118	6	0	0.174923	-10.072005	-4.253210
119	1	0	0.231589	-7.921696	-4.157882
120	6	0	0.102848	-11.258592	-3.526692
121	1	0	-0.038779	-12.133585	-1.564215
122	1	0	0.240918	-10.099438	-5.336150
123	6	0	-2.235700	-8.006394	-0.623170
124	6	0	-2.632337	-8.526229	0.612198
125	6	0	-2.966795	-8.343066	-1.766095
126	6	0	-3.735903	-9.369318	0.702951
127	1	0	-2.072594	-8.261485	1.504928
128	6	0	-4.074279	-9.180766	-1.675493
129	1	0	-2.658768	-7.947457	-2.729876
130	6	0	-4.460536	-9.697756	-0.440678
131	1	0	-4.030556	-9.768837	1.668047
132	1	0	-4.634936	-9.431130	-2.570390
133	6	0	-2.235201	-5.444436	0.771468
134	6	0	-3.482717	-5.254451	0.170114
135	6	0	-2.096190	-5.184350	2.138446
136	6	0	-4.564667	-4.798178	0.917374
137	1	0	-3.599761	-5.462676	-0.889694
138	6	0	-3.176652	-4.726806	2.886169
139	1	0	-1.128827	-5.325628	2.612523
140	6	0	-4.413538	-4.529321	2.276087
141	1	0	-5.525680	-4.647544	0.436233
142	1	0	-3.048313	-4.515721	3.942952
143	1	0	-5.255575	-4.166438	2.856409
144	9	0	-1.074323	-2.106431	4.408515
145	9	0	1.074323	2.106431	4.408515
146	1	0	-0.113793	12.214062	-4.041139
147	1	0	-5.463513	8.052443	-4.510665
148	1	0	-5.387441	2.040785	-1.096707
149	1	0	5.322535	10.353239	-0.369935
150	1	0	5.387441	-2.040785	-1.096707
151	1	0	5.463513	-8.052443	-4.510665
152	1	0	0.113793	-12.214062	-4.041139
153	1	0	-5.322535	-10.353239	-0.369935

$(Ph_5Ph)_2F_2PyBTM$ (D_1 state)

Standard orientation:

Center Number	Atomic Number	Atomic Type	Coordinates (Angstroms) X	Y	Z
1	6	0	6.607729	1.658663	-1.061492
2	6	0	5.394986	1.416786	-0.392619
3	6	0	5.850200	-0.980478	-0.434901
4	6	0	5.020313	0.097838	-0.077935
5	6	0	3.738800	-0.152032	0.643446
6	6	0	3.527773	0.381436	1.918751
7	6	0	2.721884	-0.907916	0.064139
8	1	0	4.287502	1.004924	2.377708
9	6	0	2.336667	0.165794	2.585637
10	6	0	1.544747	-1.127303	0.759626
11	1	0	2.857843	-1.350704	-0.916602
12	17	0	2.129608	1.012542	4.097898
13	6	0	1.272926	-0.601230	2.048305
14	17	0	0.341821	-2.129023	-0.022896
15	6	0	-0.012431	-0.806922	2.700861
16	6	0	-0.100229	-1.152078	4.091474
17	6	0	-1.204379	-0.674524	1.932530
18	6	0	0.909217	-1.798634	4.836732
19	6	0	-1.228450	-0.877419	4.892464
20	6	0	-2.302951	-1.647747	1.968420
21	6	0	-1.458271	0.440892	1.027758
22	6	0	0.771263	-2.111989	6.173602
23	6	0	-1.321087	-1.227076	6.226134
24	17	0	-2.076624	-3.121038	2.841741
25	6	0	-3.478018	-1.484270	1.295654
26	17	0	-0.259337	1.683386	0.861329
27	6	0	-2.634520	0.602999	0.360955
28	7	0	-0.331002	-1.836343	6.881726
29	1	0	1.590301	-2.618875	6.676284
30	1	0	-2.228343	-0.982114	6.771486
31	6	0	-3.695600	-0.340706	0.491796
32	1	0	-4.248824	-2.242779	1.370968
33	1	0	-2.777161	1.476835	-0.264821
34	6	0	-4.984057	-0.118692	-0.184083
35	6	0	-5.522547	-1.115994	-1.026746
36	6	0	-5.676310	1.095617	0.026617
37	6	0	-6.751911	-0.892819	-1.664892
38	6	0	-7.445008	0.317383	-1.453823
39	6	0	7.441841	0.582068	-1.415916
40	6	0	-6.909077	1.309952	-0.608580
41	6	0	7.062626	-0.737028	-1.104696
42	6	0	5.434564	-2.376830	-0.099660
43	6	0	5.244551	-2.759878	1.231508
44	6	0	5.205650	-3.312541	-1.112700
45	6	0	4.829448	-4.050654	1.544050
46	1	0	5.413893	-2.035713	2.023551
47	6	0	4.787442	-4.602879	-0.801306
48	1	0	5.359030	-3.022962	-2.148537
49	6	0	4.597566	-4.975028	0.527839
50	1	0	4.681265	-4.331552	2.581752
51	1	0	4.607957	-5.317692	-1.597988
52	1	0	4.269153	-5.980410	0.770595
53	6	0	7.949122	-1.880331	-1.482045
54	6	0	8.502689	-2.704267	-0.498023
55	6	0	8.235649	-2.143068	-2.824768
56	6	0	9.328704	-3.767916	-0.848778
57	1	0	8.277248	-2.509483	0.546770
58	6	0	9.056456	-3.210257	-3.177092
59	1	0	7.814431	-1.501253	-3.593498
60	6	0	9.606420	-4.024792	-2.189635
61	1	0	9.754398	-4.397464	-0.073962
62	1	0	9.267621	-3.404735	-4.223767
63	6	0	8.734958	0.842467	-2.119869
64	6	0	8.748818	1.432409	-3.386930
65	6	0	9.949910	0.502738	-1.518039
66	6	0	9.953021	1.676192	-4.040404
67	1	0	7.807823	1.705067	-3.856517
68	6	0	11.155114	0.752239	-2.167591
69	1	0	9.944807	0.036461	-0.536773
70	6	0	11.159757	1.338429	-3.431459
71	1	0	9.948726	2.131913	-5.025409
72	1	0	12.091169	0.486951	-1.686610
73	6	0	7.010580	3.060546	-1.390072
74	6	0	6.239554	3.835862	-2.260623
75	6	0	8.163772	3.616622	-0.829106
76	6	0	6.615736	5.140222	-2.567473
77	1	0	5.338238	3.411196	-2.693848
78	6	0	8.537619	4.922800	-1.130367
79	1	0	8.770761	3.015799	-0.157618
80	6	0	7.765214	5.687649	-2.001920
81	1	0	6.009567	5.729769	-3.247885
82	1	0	9.433692	5.342946	-0.684921
83	6	0	4.501490	2.554129	-0.012043
84	6	0	4.931642	3.518315	0.904283
85	6	0	3.217002	2.660451	-0.553820
86	6	0	4.091877	4.562883	1.278867
87	1	0	5.931233	3.444065	1.323415
88	6	0	2.381602	3.713252	-0.190299
89	1	0	2.872672	1.910547	-1.260999
90	6	0	2.814540	4.663776	0.731810
91	1	0	4.435953	5.298932	1.998221
92	1	0	1.391483	3.791286	-0.629149
93	6	0	-4.750023	-2.372892	-1.248536
94	6	0	-5.302329	-3.618225	-0.928807
95	6	0	-3.440272	-2.316966	-1.741923
96	6	0	-4.552230	-4.779719	-1.078449
97	1	0	-6.320015	-3.669279	-0.553099
98	6	0	-2.693924	-3.481408	-1.900568
99	1	0	-3.007601	-1.354866	-2.003549
100	6	0	-3.246037	-4.714355	-1.562696
101	1	0	-4.986595	-5.738041	-0.813562
102	1	0	-1.682295	-3.423905	-2.289351
103	6	0	-7.318511	-1.934091	-2.575629
104	6	0	-6.633211	-2.306978	-3.735136
105	6	0	-8.537936	-2.549229	-2.279016
106	6	0	-7.159228	-3.275221	-4.585137
107	1	0	-5.681741	-1.836128	-3.966449
108	6	0	-9.060885	-3.522407	-3.125103
109	1	0	-9.078108	-2.257041	-1.382900
110	6	0	-8.373501	-3.886631	-4.281012
111	1	0	-6.619295	-3.553169	-5.484497
112	1	0	-10.006889	-3.995205	-2.881879
113	6	0	-8.757816	0.547834	-2.129053
114	6	0	-9.922641	0.713112	-1.374326
115	6	0	-8.837561	0.600906	-3.523486
116	6	0	-11.146033	0.923044	-2.003015
117	1	0	-9.864612	0.679873	-0.290092
118	6	0	-10.059452	0.820598	-4.151980
119	1	0	-7.936237	0.465319	-4.114520
120	6	0	-11.217025	0.979840	-3.393292
121	1	0	-12.044095	1.044786	-1.406134
122	1	0	-10.107383	0.864808	-5.235196
123	6	0	-7.654842	2.584634	-0.377557
124	6	0	-8.101062	2.920300	0.903597
125	6	0	-7.911801	3.458092	-1.438004
126	6	0	-8.795415	4.106830	1.120238
127	1	0	-7.897764	2.247569	1.732221
128	6	0	-8.600039	4.647745	-1.220475
129	1	0	-7.573641	3.197488	-2.436963
130	6	0	-9.044991	4.974089	0.058979
131	1	0	-9.140254	4.354248	2.118976
132	1	0	-8.790590	5.319271	-2.051352
133	6	0	-5.090955	2.105124	0.955523
134	6	0	-4.807060	3.405055	0.519059
135	6	0	-4.762275	1.743646	2.269273
136	6	0	-4.183084	4.310638	1.369359
137	1	0	-5.066722	3.695931	-0.494487
138	6	0	-4.137171	2.650686	3.119273
139	1	0	-4.990974	0.741553	2.621407
140	6	0	-3.841775	3.934654	2.669110
141	1	0	-3.954463	5.310440	1.015300
142	1	0	-3.872588	2.346834	4.126657
143	1	0	-3.346998	4.641466	3.326860
144	9	0	-2.274230	-0.241467	4.325938
145	9	0	2.050923	-2.144792	4.219600
146	1	0	12.099226	1.531222	-3.939367
147	1	0	8.057611	6.705476	-2.239113
148	1	0	2.160425	5.479834	1.020959
149	1	0	10.248429	-4.855741	-2.463662
150	1	0	-2.662745	-5.621784	-1.678238
151	1	0	-8.782777	-4.643535	-4.942157
152	1	0	-12.170596	1.147314	-3.883401
153	1	0	-9.584369	5.900386	0.228003

$(Me_4Ph_5Ph)_2F_2PyBTM$ (D_0 state)

Standard orientation:

Center Number	Atomic Number	Atomic Type	Coordinates (Angstroms) X	Y	Z
1	6	0	6.594133	1.331463	-1.343269
2	6	0	5.357640	1.281054	-0.675750
3	6	0	5.898720	-0.951380	0.156254
4	6	0	5.017244	0.141489	0.074288
5	6	0	3.717603	0.100757	0.808834
6	6	0	3.452147	1.031917	1.813743
7	6	0	2.760500	-0.869520	0.522270
8	1	0	4.170216	1.812514	2.039156
9	6	0	2.261056	0.977480	2.514371
10	6	0	1.571802	-0.908764	1.236565
11	1	0	2.949893	-1.617831	-0.239405
12	17	0	1.985418	2.233690	3.694448
13	6	0	1.270912	0.007074	2.261507
14	17	0	0.465978	-2.204481	0.852342
15	6	0	0.000505	-0.032942	3.003004
16	6	0	-0.003683	-0.041050	4.449696
17	6	0	-1.266696	-0.063022	2.255566
18	6	0	1.049099	-0.556096	5.226922
19	6	0	-1.060827	0.464903	5.227111
20	6	0	-2.258865	-1.035231	2.493164
21	6	0	-1.563883	0.867239	1.242717
22	6	0	1.016782	-0.551471	6.610993
23	6	0	-1.036118	0.443611	6.611201
24	17	0	-1.986794	-2.308499	3.655705
25	6	0	-3.449902	-1.076477	1.791563
26	17	0	-0.454767	2.165409	0.877029
27	6	0	-2.752156	0.841022	0.527311
28	7	0	-0.011624	-0.058104	7.302360
29	1	0	1.855894	-0.968737	7.159316
30	1	0	-1.878255	0.854365	7.159812
31	6	0	-3.712714	-0.129498	0.800892
32	1	0	-4.170452	-1.857926	2.005871
33	1	0	-2.939276	1.600657	-0.223630
34	6	0	-5.014501	-0.151209	0.069545
35	6	0	-5.364433	-1.276437	-0.697393
36	6	0	-5.888490	0.946048	0.171924
37	6	0	-6.603094	-1.307868	-1.362036
38	6	0	-7.482406	-0.212535	-1.261765
39	6	0	7.481260	0.240929	-1.262443
40	6	0	-7.124384	0.915665	-0.498726
41	6	0	7.132677	-0.901716	-0.516841
42	6	0	5.505309	-2.156397	0.949757
43	6	0	5.238036	-2.050130	2.318251
44	6	0	5.351070	-3.397504	0.325194
45	6	0	4.809378	-3.156670	3.044808
46	1	0	5.351462	-1.088172	2.810479
47	6	0	4.923194	-4.505316	1.050967
48	1	0	5.565951	-3.489043	-0.735778
49	6	0	4.647546	-4.387069	2.411648
50	1	0	4.593419	-3.054103	4.103474
51	1	0	4.800429	-5.461084	0.551713
52	1	0	4.307414	-5.249552	2.975543
53	6	0	8.068909	-2.063818	-0.439859
54	6	0	8.589292	-2.483624	0.786852
55	6	0	8.435819	-2.762146	-1.593383
56	6	0	9.456397	-3.568921	0.855419
57	1	0	8.303662	-1.959077	1.694542
58	6	0	9.297493	-3.850905	-1.519257
59	1	0	8.041707	-2.445923	-2.555249
60	6	0	9.826262	-4.269569	-0.295296
61	1	0	9.850713	-3.880087	1.819233
62	1	0	9.564922	-4.386004	-2.426709
63	6	0	8.798295	0.299639	-1.966761
64	6	0	8.861537	0.426745	-3.357053
65	6	0	9.994892	0.230011	-1.249288
66	6	0	10.088197	0.481398	-4.009084
67	1	0	7.939569	0.486107	-3.928904
68	6	0	11.219936	0.292355	-1.905100
69	1	0	9.962607	0.118185	-0.169028
70	6	0	11.287770	0.420012	-3.294525
71	1	0	10.116512	0.573180	-5.091763
72	1	0	12.139845	0.237442	-1.328950
73	6	0	6.962016	2.547170	-2.130498
74	6	0	6.197530	2.942979	-3.231038
75	6	0	8.077834	3.313442	-1.784280
76	6	0	6.543426	4.071588	-3.966173
77	1	0	5.324955	2.359134	-3.511049
78	6	0	8.415782	4.445612	-2.517787
79	1	0	8.689835	3.011534	-0.938819
80	6	0	7.654844	4.844340	-3.619739
81	1	0	5.940909	4.358418	-4.824116
82	1	0	9.287626	5.028606	-2.232694
83	6	0	4.402223	2.428527	-0.739540
84	6	0	4.745226	3.676403	-0.213554
85	6	0	3.126577	2.263355	-1.286701
86	6	0	3.827496	4.722067	-0.214052
87	1	0	5.738142	3.824896	0.202108
88	6	0	2.216816	3.314493	-1.295407
89	1	0	2.844501	1.299414	-1.701800
90	6	0	2.546708	4.557220	-0.746941
91	1	0	4.109332	5.682428	0.209236
92	1	0	1.230695	3.166892	-1.728307
93	6	0	-4.416375	-2.428703	-0.781165
94	6	0	-4.766774	-3.683095	-0.275986
95	6	0	-3.139410	-2.261920	-1.324946
96	6	0	-3.854895	-4.733767	-0.293200
97	1	0	-5.760729	-3.832806	0.136726
98	6	0	-2.235339	-3.317654	-1.349913
99	1	0	-2.851593	-1.292937	-1.724026
100	6	0	-2.572741	-4.567401	-0.822311
101	1	0	-4.142466	-5.699380	0.113974
102	1	0	-1.247982	-3.168370	-1.779402
103	6	0	-6.981586	-2.508332	-2.167330
104	6	0	-6.221148	-2.893683	-3.274371
105	6	0	-8.103984	-3.269983	-1.832330
106	6	0	-6.577685	-4.007337	-4.027028
107	1	0	-5.343557	-2.313329	-3.545816
108	6	0	-8.452471	-4.387398	-2.583309
109	1	0	-8.712806	-2.975919	-0.981835
110	6	0	-7.695889	-4.775364	-3.692095
111	1	0	-5.978308	-4.285953	-4.889863
112	1	0	-9.329352	-4.966972	-2.306823
113	6	0	-8.800889	-0.250796	-1.964719
114	6	0	-9.995942	-0.181283	-1.244710
115	6	0	-8.866936	-0.358496	-3.356543
116	6	0	-11.222289	-0.224742	-1.899576
117	1	0	-9.961350	-0.084454	-0.163078
118	6	0	-10.094834	-0.394366	-4.007512
119	1	0	-7.946238	-0.417481	-3.930459
120	6	0	-11.292952	-0.332953	-3.290497
121	1	0	-12.140978	-0.170178	-1.321449
122	1	0	-10.125306	-0.471159	-5.091299
123	6	0	-8.051650	2.083445	-0.401359
124	6	0	-8.564047	2.489830	0.833245
125	6	0	-8.416835	2.801520	-1.543252
126	6	0	-9.421540	3.581353	0.920783
127	1	0	-8.279492	1.949872	1.732192
128	6	0	-9.268903	3.896368	-1.450148
129	1	0	-8.028912	2.495987	-2.511075
130	6	0	-9.789578	4.301741	-0.218271
131	1	0	-9.809653	3.881854	1.890484
132	1	0	-9.535051	4.446824	-2.348745
133	6	0	-5.483602	2.135280	0.983416
134	6	0	-5.322795	3.385281	0.378565
135	6	0	-5.208899	2.004236	2.348330
136	6	0	-4.880783	4.477168	1.119863
137	1	0	-5.543303	3.496286	-0.679391
138	6	0	-4.765299	3.094564	3.090229
139	1	0	-5.327221	1.035221	2.825307
140	6	0	-4.596876	4.333873	2.476437
141	1	0	-4.752988	5.440040	0.635751
142	1	0	-4.542244	2.972008	4.145276
143	1	0	-4.245058	5.183815	3.052086
144	6	0	-10.740846	5.466995	-0.124986
145	1	0	-10.538965	6.205632	-0.904375
146	1	0	-11.777008	5.134480	-0.247195
147	1	0	-10.666259	5.961770	0.846248
148	6	0	-12.621782	-0.407766	-3.997689
149	1	0	-12.575489	0.072985	-4.977916
150	1	0	-12.920312	-1.449249	-4.156759
151	1	0	-13.408497	0.075661	-3.413915
152	6	0	-8.059542	-6.002149	-4.488460
153	1	0	-7.772265	-5.892447	-5.536925
154	1	0	-7.545759	-6.886623	-4.097492
155	1	0	-9.133345	-6.198603	-4.444168
156	6	0	-1.568470	-5.690754	-0.803554
157	1	0	-0.963324	-5.694992	-1.713640
158	1	0	-0.883956	-5.583560	0.044590
159	1	0	-2.060724	-6.661496	-0.712080
160	6	0	10.787771	-5.427863	-0.222505
161	1	0	10.588593	-6.157240	-1.011258
162	1	0	11.820520	-5.084873	-0.344651
163	1	0	10.721767	-5.936767	0.742020
164	6	0	12.614957	0.515311	-4.002340
165	1	0	12.570949	0.048456	-4.989364
166	1	0	12.905219	1.561265	-4.146699
167	1	0	13.406233	0.029591	-3.426686
168	6	0	8.006695	6.087001	-4.396551
169	1	0	7.719024	5.991797	-5.446321
170	1	0	7.485689	6.960467	-3.990676
171	1	0	9.078791	6.292141	-4.350307
172	6	0	1.536119	5.674470	-0.710620
173	1	0	0.930756	5.689395	-1.620443
174	1	0	0.852417	5.550304	0.135868
175	1	0	2.022917	6.646440	-0.604156
176	9	0	-2.125093	1.004086	4.623364
177	9	0	2.116581	-1.087547	4.622272

$(Me_4Ph_5Ph)_2F_2PyBTM$ (D_1 state)

Standard orientation:

Center Number	Atomic Number	Atomic Type	Coordinates (Angstroms) X	Y	Z
1	6	0	-6.636352	-1.133740	-1.338123
2	6	0	-5.432311	-1.241262	-0.625364
3	6	0	-5.637902	1.106416	0.064743
4	6	0	-4.933120	-0.119275	0.074411
5	6	0	-3.673118	-0.229166	0.825654
6	6	0	-3.487611	-1.246743	1.795511
7	6	0	-2.606197	0.684875	0.599179
8	1	0	-4.271188	-1.977517	1.960144
9	6	0	-2.329303	-1.330195	2.508831
10	6	0	-1.446061	0.601630	1.308385
11	1	0	-2.726748	1.470850	-0.137831
12	17	0	-2.146615	-2.675093	3.581846
13	6	0	-1.211361	-0.398628	2.347505
14	17	0	-0.231666	1.802735	1.009840
15	6	0	-0.009642	-0.460706	3.110183
16	6	0	-0.076498	-0.623578	4.534568
17	6	0	1.266790	-0.361383	2.416276
18	6	0	-1.183261	-0.226736	5.314073
19	6	0	0.937267	-1.186824	5.339046
20	6	0	2.349427	0.454664	2.829384
21	6	0	1.508637	-1.052800	1.201546
22	6	0	-1.253740	-0.399577	6.683065
23	6	0	0.821949	-1.322756	6.707549
24	17	0	2.181151	1.493245	4.222505
25	6	0	3.528130	0.569030	2.117270
26	17	0	0.280758	-2.133732	0.578668
27	6	0	2.672087	-0.936832	0.459701
28	7	0	-0.260698	-0.937878	7.394499
29	1	0	-2.145671	-0.069414	7.208449
30	1	0	1.643394	-1.775377	7.256123
31	6	0	3.707697	-0.123741	0.915585
32	1	0	4.302109	1.238152	2.477264
33	1	0	2.782261	-1.502033	-0.459417
34	6	0	4.971338	0.024301	0.136363
35	6	0	5.349987	1.294632	-0.336161
36	6	0	5.777320	-1.099056	-0.124274
37	6	0	6.541499	1.443325	-1.067721
38	6	0	7.351724	0.321469	-1.326155
39	6	0	-7.340835	0.087937	-1.347422
40	6	0	6.969133	-0.949371	-0.856219
41	6	0	-6.844445	1.205801	-0.643738
42	6	0	-5.093877	2.255800	0.844820
43	6	0	-4.823935	2.112010	2.212391
44	6	0	-4.791891	3.471086	0.218714
45	6	0	-4.239275	3.149015	2.932851
46	1	0	-5.066744	1.176958	2.709773
47	6	0	-4.207157	4.506374	0.939257
48	1	0	-5.007646	3.593986	-0.838481
49	6	0	-3.924344	4.346468	2.296101
50	1	0	-4.022379	3.014814	3.987430
51	1	0	-3.964353	5.438592	0.439908
52	1	0	-3.460791	5.153742	2.853372
53	6	0	-7.606167	2.491061	-0.639613
54	6	0	-8.102790	3.018828	0.554282
55	6	0	-7.830914	3.193193	-1.826625
56	6	0	-8.810768	4.216135	0.558478
57	1	0	-7.926131	2.489332	1.486605
58	6	0	-8.532499	4.393331	-1.815921
59	1	0	-7.454336	2.792202	-2.763581
60	6	0	-9.038000	4.922599	-0.625012
61	1	0	-9.190334	4.611037	1.497036
62	1	0	-8.691514	4.929647	-2.747690
63	6	0	-8.619115	0.200769	-2.111072
64	6	0	-8.638059	0.023463	-3.496854
65	6	0	-9.819128	0.493958	-1.457774
66	6	0	-9.826441	0.141960	-4.209068
67	1	0	-7.713487	-0.208185	-4.018292
68	6	0	-11.006244	0.600528	-2.173377
69	1	0	-9.818083	0.647092	-0.382207
70	6	0	-11.030451	0.426749	-3.559858
71	1	0	-9.820143	0.010427	-5.287796
72	1	0	-11.930082	0.827391	-1.647875
73	6	0	-7.162062	-2.311821	-2.091811
74	6	0	-6.432279	-2.865898	-3.146181
75	6	0	-8.393338	-2.881561	-1.757903
76	6	0	-6.925069	-3.959446	-3.850080
77	1	0	-5.471884	-2.434657	-3.415494
78	6	0	-8.877690	-3.979977	-2.459532
79	1	0	-8.977976	-2.452887	-0.948651
80	6	0	-8.152474	-4.537497	-3.516110
81	1	0	-6.347624	-4.371753	-4.673233
82	1	0	-9.837438	-4.410092	-2.185802
83	6	0	-4.642018	-2.504975	-0.615260
84	6	0	-5.191408	-3.696576	-0.129903
85	6	0	-3.309214	-2.511381	-1.049160
86	6	0	-4.417629	-4.847680	-0.047004
87	1	0	-6.227489	-3.713119	0.195376
88	6	0	-2.546057	-3.671524	-0.978900
89	1	0	-2.870971	-1.600490	-1.448847
90	6	0	-3.081170	-4.853851	-0.461536
91	1	0	-4.855695	-5.759155	0.350080
92	1	0	-1.518472	-3.659573	-1.332911
93	6	0	4.480294	2.477190	-0.055032
94	6	0	4.941639	3.533161	0.733504
95	6	0	3.176789	2.539890	-0.557246
96	6	0	4.114577	4.613109	1.028251
97	1	0	5.956749	3.505024	1.120133
98	6	0	2.361008	3.629475	-0.275612
99	1	0	2.800676	1.725098	-1.170488
100	6	0	2.810664	4.677784	0.533287
101	1	0	4.488209	5.419217	1.653812
102	1	0	1.355994	3.668656	-0.689217
103	6	0	6.942997	2.793076	-1.568749
104	6	0	6.141337	3.483661	-2.481210
105	6	0	8.130402	3.391827	-1.139769
106	6	0	6.520082	4.736067	-2.952880
107	1	0	5.213193	3.033107	-2.822047
108	6	0	8.501971	4.647091	-1.608680
109	1	0	8.771195	2.862152	-0.440237
110	6	0	7.703369	5.340856	-2.521669
111	1	0	5.886663	5.253775	-3.668502
112	1	0	9.429634	5.095747	-1.262939
113	6	0	8.619501	0.479787	-2.101879
114	6	0	9.854614	0.174976	-1.524047
115	6	0	8.596279	0.930718	-3.423956
116	6	0	11.032452	0.322159	-2.248494
117	1	0	9.888384	-0.189256	-0.500968
118	6	0	9.775971	1.069736	-4.147236
119	1	0	7.643957	1.175399	-3.886402
120	6	0	11.013672	0.772852	-3.571045
121	1	0	11.983345	0.079963	-1.781003
122	1	0	9.736195	1.414729	-5.177166
123	6	0	7.830118	-2.139430	-1.132454
124	6	0	8.407868	-2.861744	-0.085454
125	6	0	8.074335	-2.556511	-2.443515
126	6	0	9.212226	-3.966409	-0.344577
127	1	0	8.217381	-2.557491	0.940131
128	6	0	8.873356	-3.665784	-2.697864
129	1	0	7.635207	-2.002774	-3.268839
130	6	0	9.459802	-4.386212	-1.653919
131	1	0	9.653079	-4.514813	0.483794
132	1	0	9.045575	-3.978796	-3.724399
133	6	0	5.358516	-2.443515	0.377691
134	6	0	5.094418	-3.485728	-0.516025
135	6	0	5.199917	-2.671252	1.748000
136	6	0	4.672223	-4.727080	-0.049579
137	1	0	5.223944	-3.317643	-1.581497
138	6	0	4.781083	-3.913037	2.215825
139	1	0	5.396580	-1.864056	2.448089
140	6	0	4.513456	-4.943723	1.317593
141	1	0	4.465300	-5.525395	-0.755158
142	1	0	4.658002	-4.072575	3.282182
143	1	0	4.181869	-5.910902	1.681267
144	6	0	10.354592	-5.566292	-1.933817
145	1	0	10.061142	-6.075318	-2.855011
146	1	0	11.395362	-5.246773	-2.051590
147	1	0	10.323024	-6.289587	-1.115564
148	6	0	12.293776	0.958575	-4.344634
149	1	0	12.131204	0.823962	-5.416640
150	1	0	12.695794	1.966348	-4.196418
151	1	0	13.058474	0.248716	-4.020367
152	6	0	8.094673	6.713202	-3.006555
153	1	0	7.695608	6.908740	-4.004655
154	1	0	7.705172	7.488249	-2.338166
155	1	0	9.180967	6.825398	-3.041784
156	6	0	1.898919	5.828171	0.873543
157	1	0	1.360951	6.180526	-0.010699
158	1	0	1.150249	5.522132	1.611568
159	1	0	2.458414	6.667205	1.292626
160	6	0	-9.828070	6.205853	-0.623590
161	1	0	-9.471822	6.890133	-1.397372
162	1	0	-10.887333	6.010172	-0.820180
163	1	0	-9.759502	6.711603	0.342213
164	6	0	-12.323104	0.518376	-4.328844
165	1	0	-12.148279	0.835157	-5.359622
166	1	0	-12.824643	-0.454417	-4.363081
167	1	0	-13.011560	1.226821	-3.862126
168	6	0	-8.667914	-5.744113	-4.257316
169	1	0	-8.308429	-5.757706	-5.288865
170	1	0	-8.329380	-6.668575	-3.777970
171	1	0	-9.760179	-5.762184	-4.272291
172	6	0	-2.232869	-6.090624	-0.329809
173	1	0	-1.513685	-6.165626	-1.148828
174	1	0	-1.664009	-6.065368	0.605588
175	1	0	-2.846361	-6.994108	-0.321880
176	9	0	2.061366	-1.629843	4.751446
177	9	0	-2.230024	0.350389	4.689850

Table S1. Calculated torsion angles of the aryl rings of the radicals in chloroform.

	$(Ph_5Ph)_2F_2PyBTM$ (D_0)	$(Ph_5Ph)_2F_2PyBTM$ (D_1)	$(Me_4Ph_5Ph)_2F_2PyBTM$ (D_0)	$(Me_4Ph_5Ph)_2F_2PyBTM$ (D_1)
φ_1	27°	25°	27°	25°
φ_2	52°	47°	52°	46°
φ_3	52°	44°	52°	45°
ϕ_1	62°	54°	62°	54°
ϕ_2	62°	56°	61°	56°
ϕ_3	62°	63°	62°	62°
ϕ_4	62°	63°	61°	62°
ϕ_5	62°	63°	61°	63°
ϕ_6	62°	59°	62°	60°
ϕ_7	62°	61°	62°	60°
ϕ_8	62°	63°	62°	63°
ϕ_9	62°	63°	62°	62°
ϕ_{10}	62°	63°	62°	62°
ϕ_{11}	62°	62°	62°	62°
ϕ_{12}	62°	62°	62°	62°

Table S2. D_1 excited states of F_2PyBTM, $(Ph_5Ph)F_2PyBTM$, and $(Me_4Ph_5Ph)F_2PyBTM$ at the D_1 optimized structure in chloroform. Major configurations are shown with the CI coefficients larger than 0.3.

	Excitation Energy /eV	Excitation Energy /nm	Oscillator Strength	Major Configurations (CI Coefficients)
F_2PyBTM	2.379	521	0.074	βHO → βLU (0.9356)
Mes_2F_2PyBTM	2.280	544	0.1445	βHO → βLU (0.8222)
$(Ph_5Ph)_2F_2PyBTM$	2.218	559	0.1330	βHO-4 → βLU (0.3947)
				βHO-1 → βLU (-0.3961)
				βHO → βLU (0.6166)
$(Me_4Ph_5Ph)_2F_2PyBTM$	2.215	560	0.1388	βHO-4 → βLU (0.6491)
				βHO-1 → βLU (-0.3190)
				βHO → βLU (-0.3705)

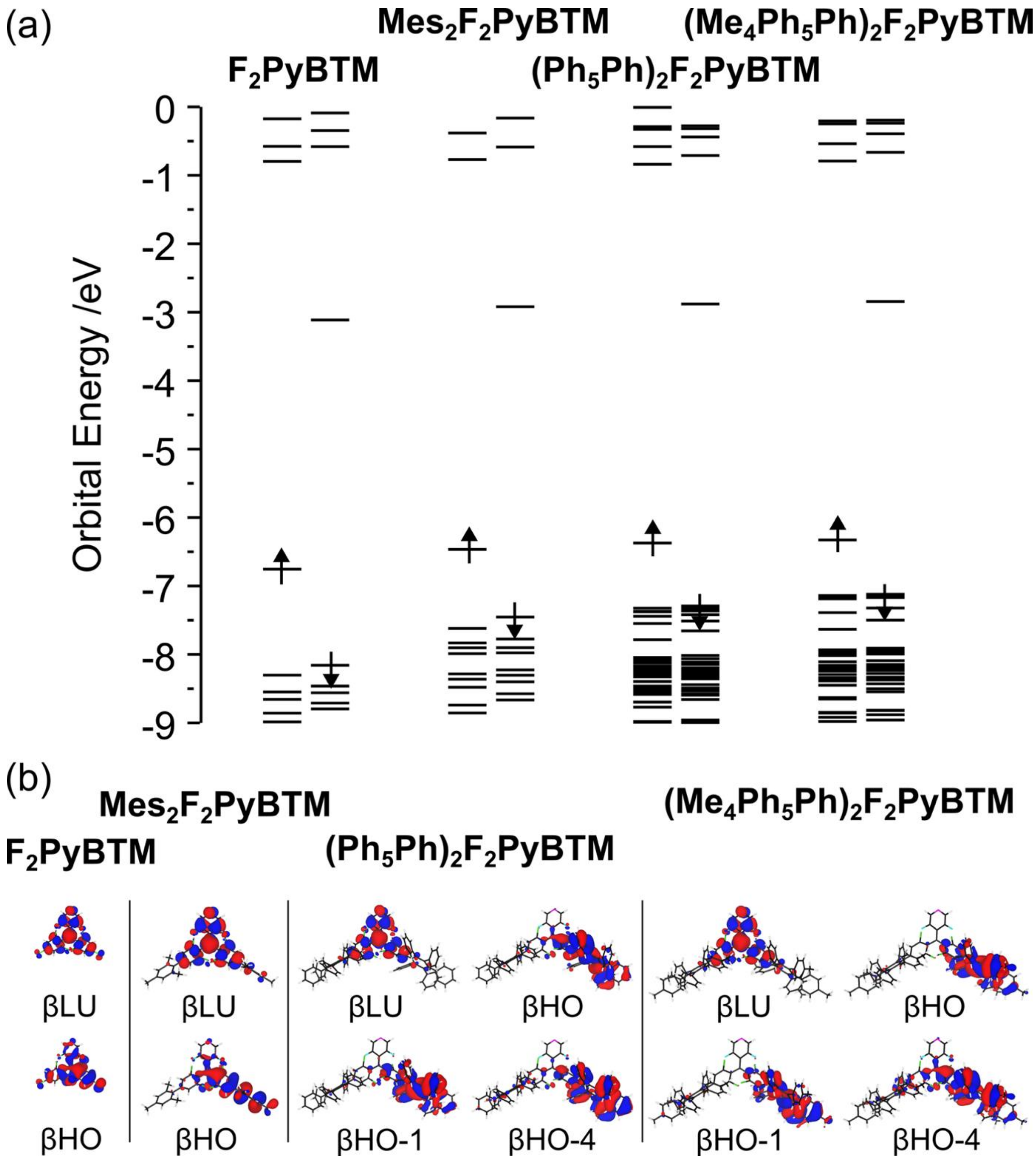

Fig. S5 (a) Orbital levels and (b) molecular orbitals (isosurface value: 2×10^{-2} a.u.) of F_2PyBTM, Mes_2F_2PyBTM, $(Ph_5Ph)_2F_2$PyBTM, and $(Me_4Ph_5Ph)_2F_2$PyBTM at the D_1 optimized structure in chloroform.

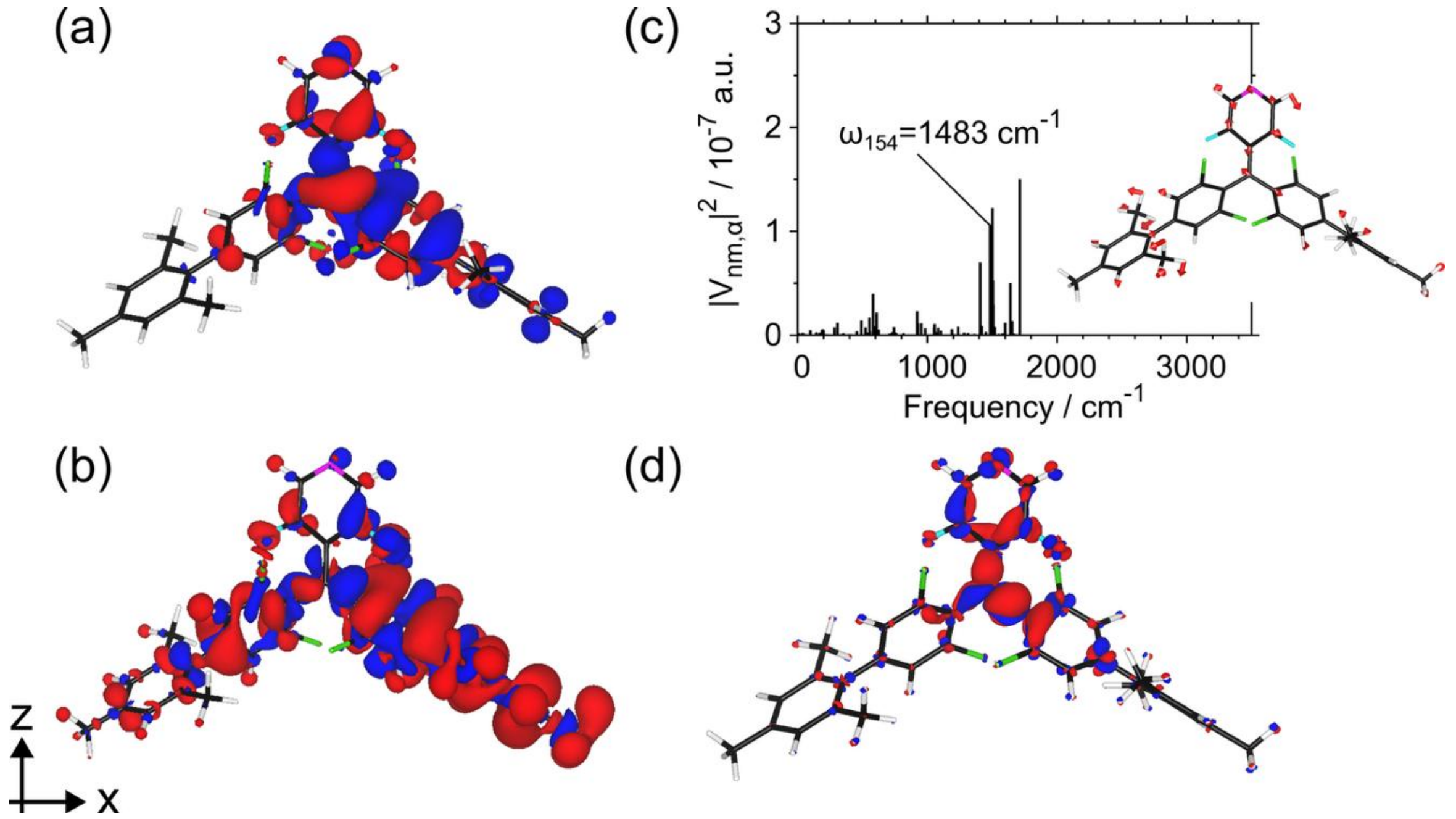

Fig. S6 (a) Overlap density (isosurface value: 5×10^{-4} a.u.), (b) transition dipole moment density in the *x* component (isosurface value: 1×10^{-3} a.u.), (c) off-diagonal vibronic coupling constant, and (d) vibronic coupling density for vibrational mode 154 (isosurface value: 3×10^{-6} a.u.) of Mes_2F_2PyBTM between D_1 and D_0 at the D_1 optimized structure in chloroform. The sign of (b) and (d) is taken so that the integral becomes positive. Red/blue region is positive/negative.

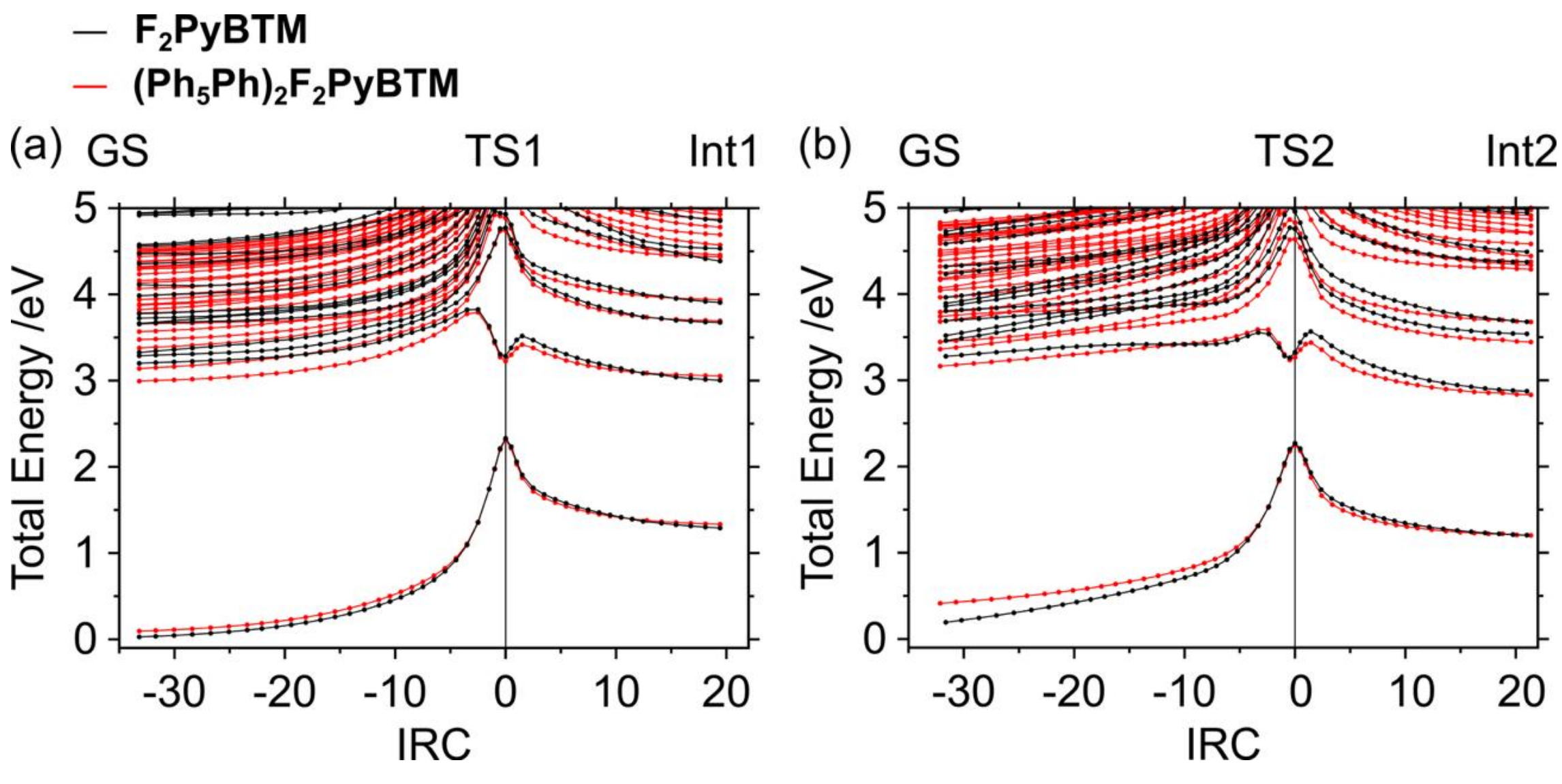

Fig. S7 Potential energy curves along intrinsic reaction coordinate for cyclization (a) between a chlorobenzene ring and a fluoropyridine ring and (b) between two chlorobenzene rings in F_2PyBTM (black lines) and $(Ph_5Ph)_2F_2$PyBTM (red lines) in cyclohexane. Energy reference is the energy of $D_0@D_0$:-3704.5603 a.u. for F_2PyBTM and-5557.0052 a.u. for $(Ph_5Ph)_2F_2$PyBTM.

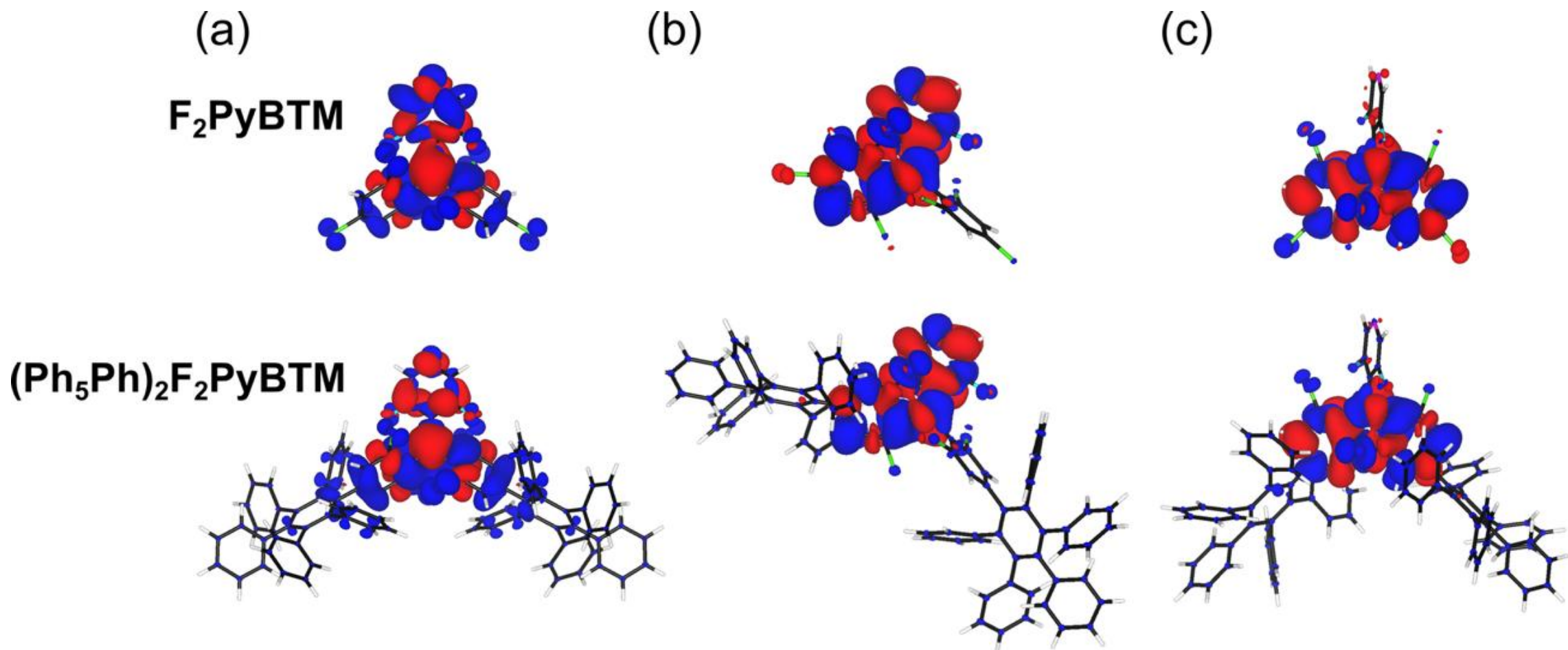

Fig. S8 Electron density difference of D_1–D_0 (a) at the ground state, and at the transition state for cyclization (b) between a chlorobenzene ring and a fluoropyridine ring and (c) between two chlorobenzene rings in F_2PyBTM (top) and $(Ph_5Ph)_2F_2$PyBTM (bottom). Isosurface value is 5×10^{-4} a.u. Red/blue region is positive/negative.

Table S3. D_1 excited states of F_2PyBTM and $(Ph_5Ph)F_2$PyBTM at the ground state and transition state structures in cyclohexane. Major configurations are shown with the CI coefficients larger than 0.3.

		Excitation Energy /eV	/nm	Oscillator Strength	Major Configurations (CI Coefficients)
F_2PyBTM	GS	3.141	395	0.015	βHO-2 → βLU (0.4962)
					βHO → βLU (0.7721)
	TS1	0.953	1302	0.008	αHO → αLU (0.9744)
	TS2	1.055	1175	0.006	αHO → αLU (0.9737)
$(Ph_5Ph)_2F_2$PyBTM	GS	2.955	420	0.099	βHO-23 → βLU (0.3125)
					βHO-18 → βLU (0.3018)
					βHO-4 → βLU (0.6259)
					βHO-2 → βLU (0.3550)
	TS1	0.911	1360	0.010	αHO → αLU (0.9725)
	TS2	1.015	1221	0.006	αHO → αLU (0.9704)

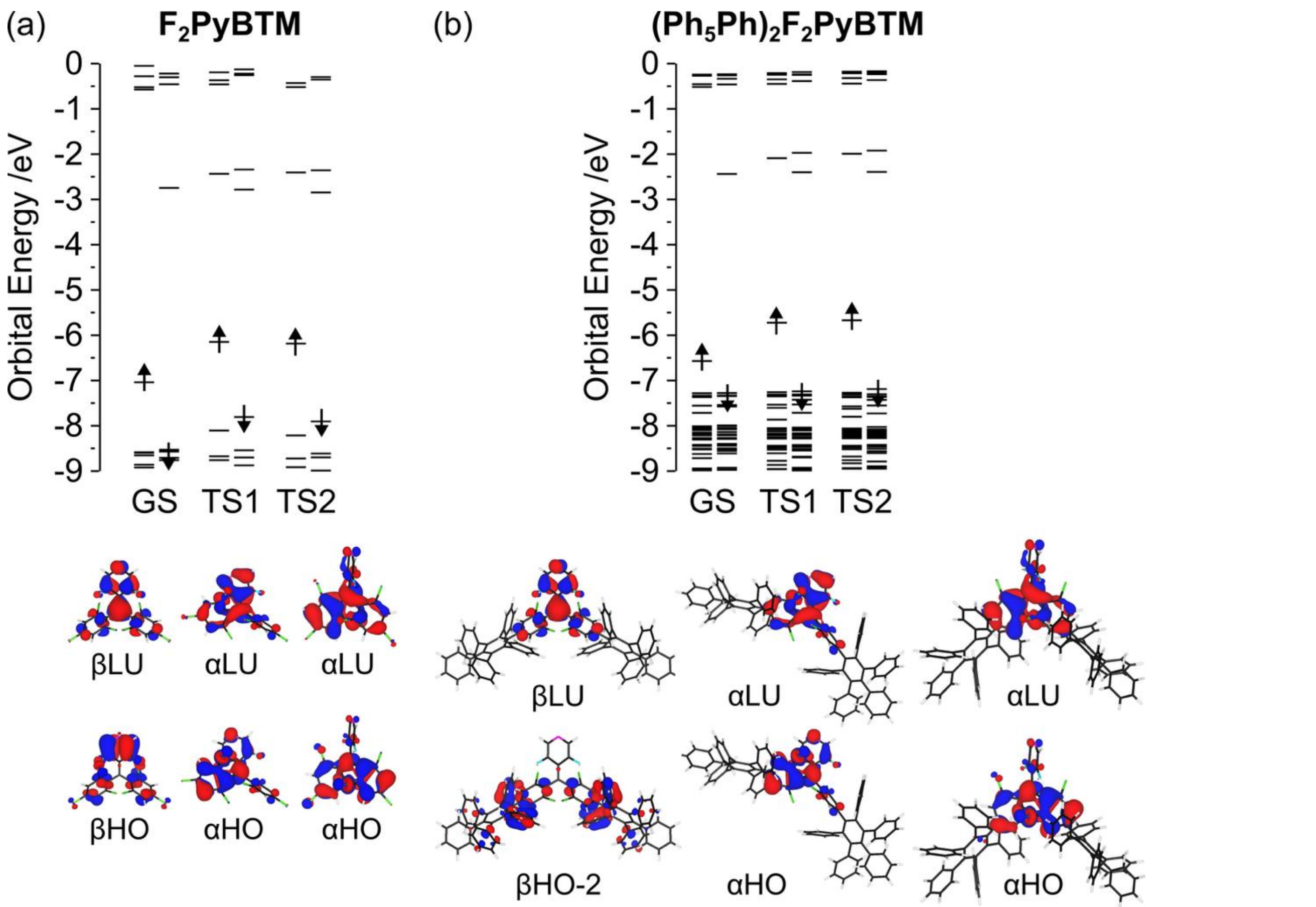

Fig. S9 Orbital levels of (a) F_2PyBTM and (b) $(Ph_5Ph)_2F_2$PyBTM at the ground state and transition state structures in cyclohexane. Also shown is the molecular orbitals of that mainly consists of the D_1 excited state (see Table S3). Isosurface value is 3×10^{-2} a.u.

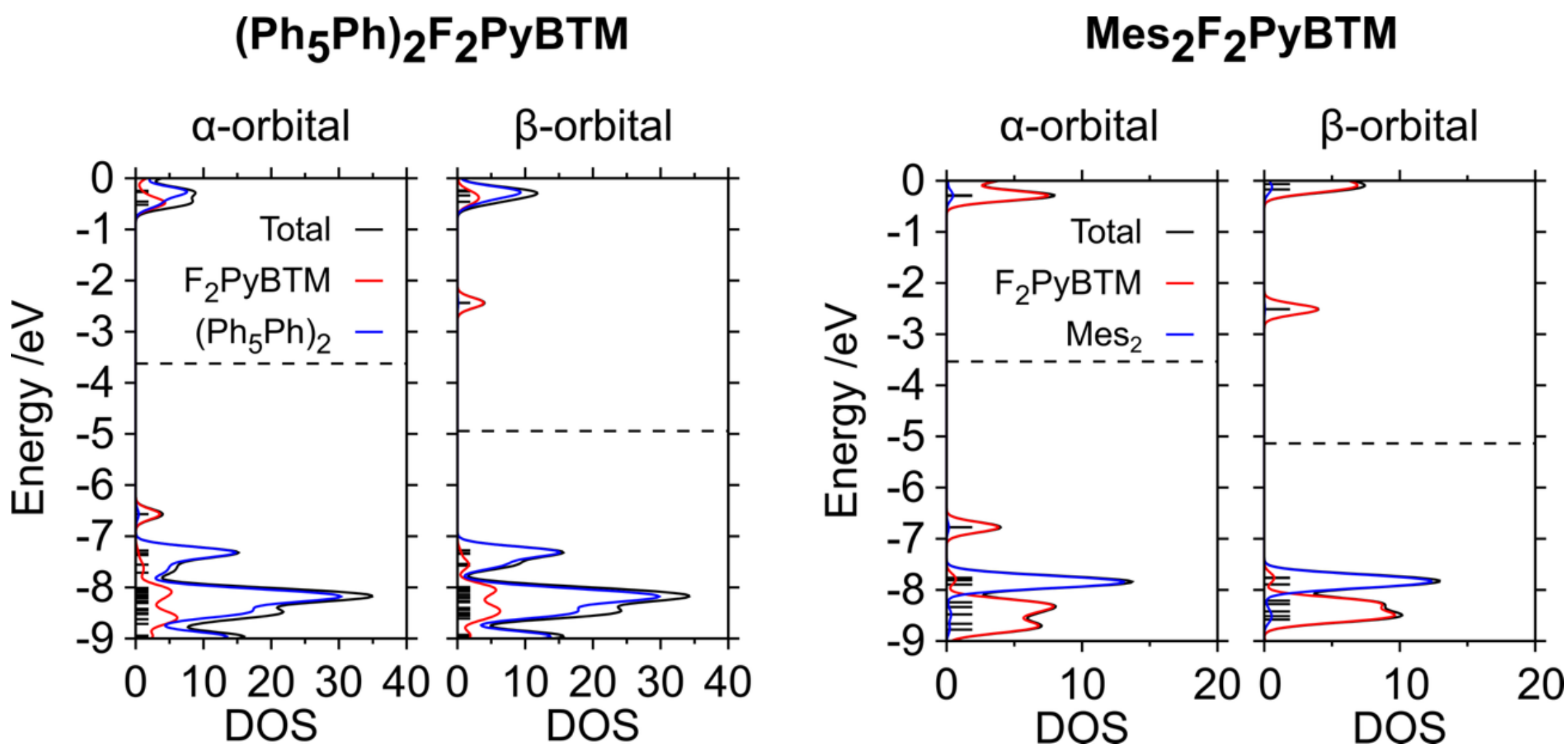

Fig. S10 Density of states (DOS) and its projection onto the F_2PyBTM moiety and donor fragment for $(Ph_5Ph)_2F_2$PyBTM and Mes_2F_2PyBTM at the D_0 optimized structure in cyclohexane. The DOS was broadened using the Gaussian function with a linewidth of 0.1 eV.

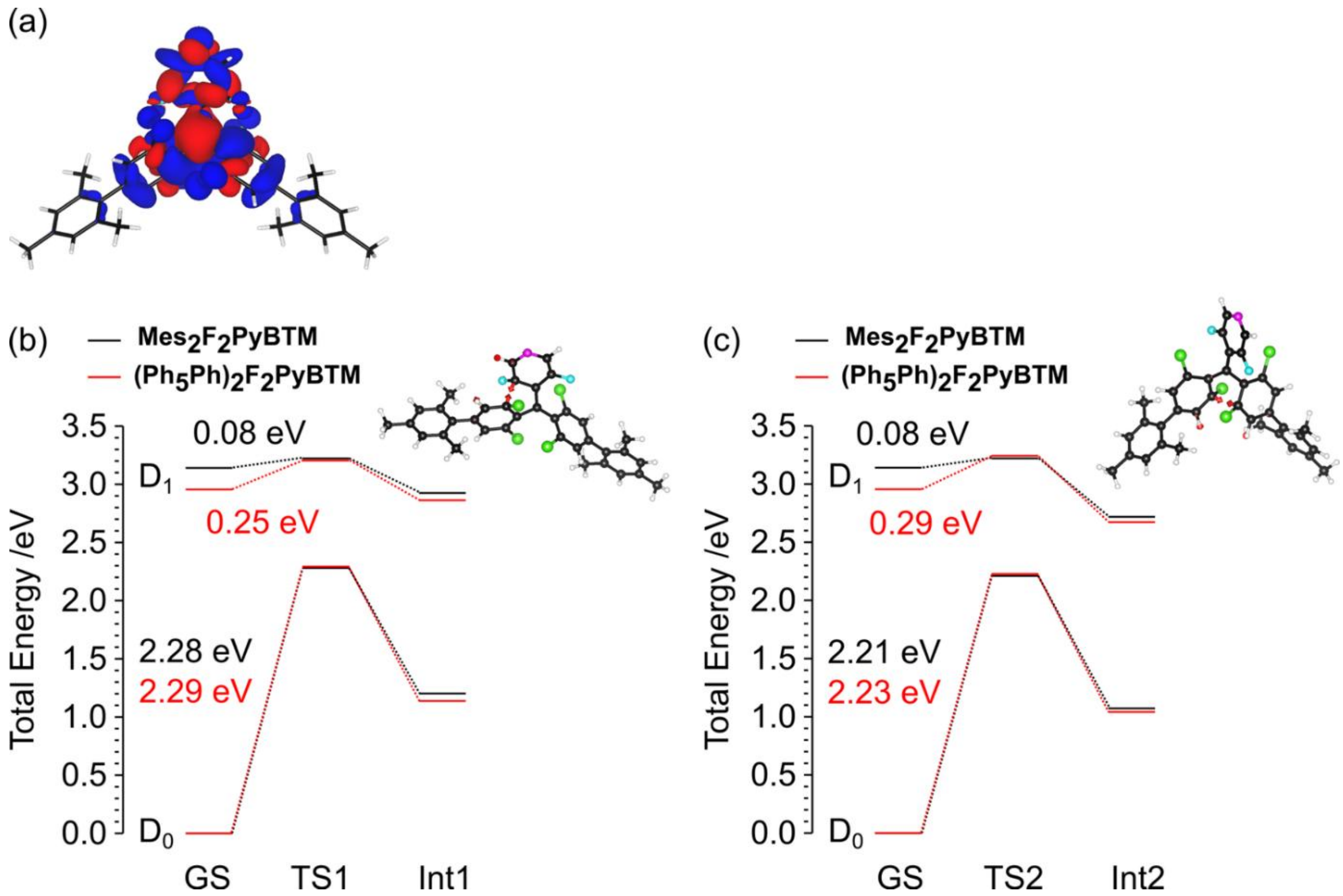

Fig. S11 (a) D_1-D_0 electron density difference of Mes_2F_2PyBTM at the ground state (isosurface value: 5×10^{-4} a.u.), Red/blue region is positive/negative. Energy diagrams for cyclization (b) between a chlorobenzene ring and a fluoropyridine ring and (c) between two chlorobenzene rings in Mes_2F_2PyBTM (black lines) and $(Ph_5Ph)_2F_2PyBTM$ (red lines) in cyclohexane. Energy reference is the energy of $D_0@D_0$:-3483.1519 a.u. for Mes_2F_2PyBTM and -5557.0052 a.u. for $(Ph_5Ph)_2F_2PyBTM$.

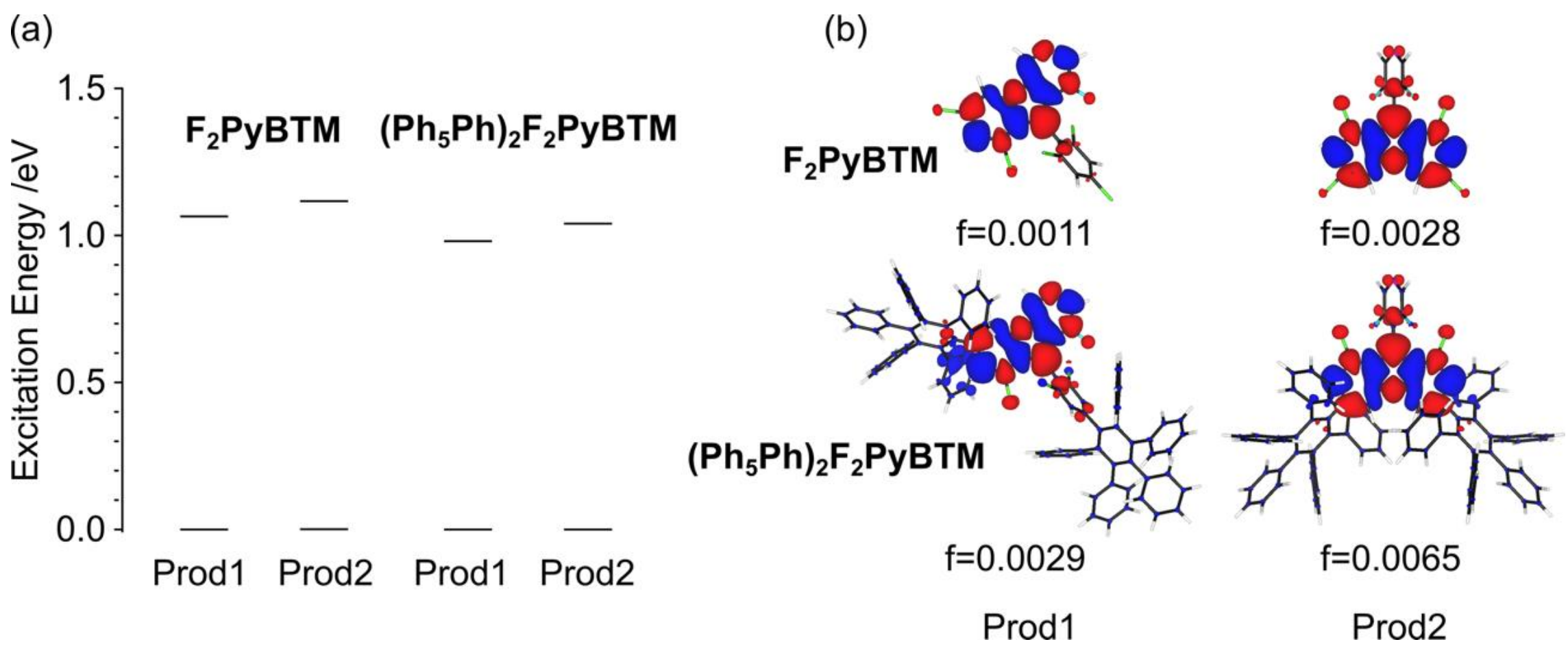

Fig. S12 (a) D_1 excitation energy at the D_1 optimized structure of the expected photodegradation products of F_2PyBTM and $(Ph_5Ph)_2F_2PyBTM$ in cyclohexane. (b) D_1-D_0 electron density difference of the expected photodegradation products. *f* represents the oscillator strength. Red/blue region is positive/negative. Isosurface value is 5×10^{-4} a.u.

(a)

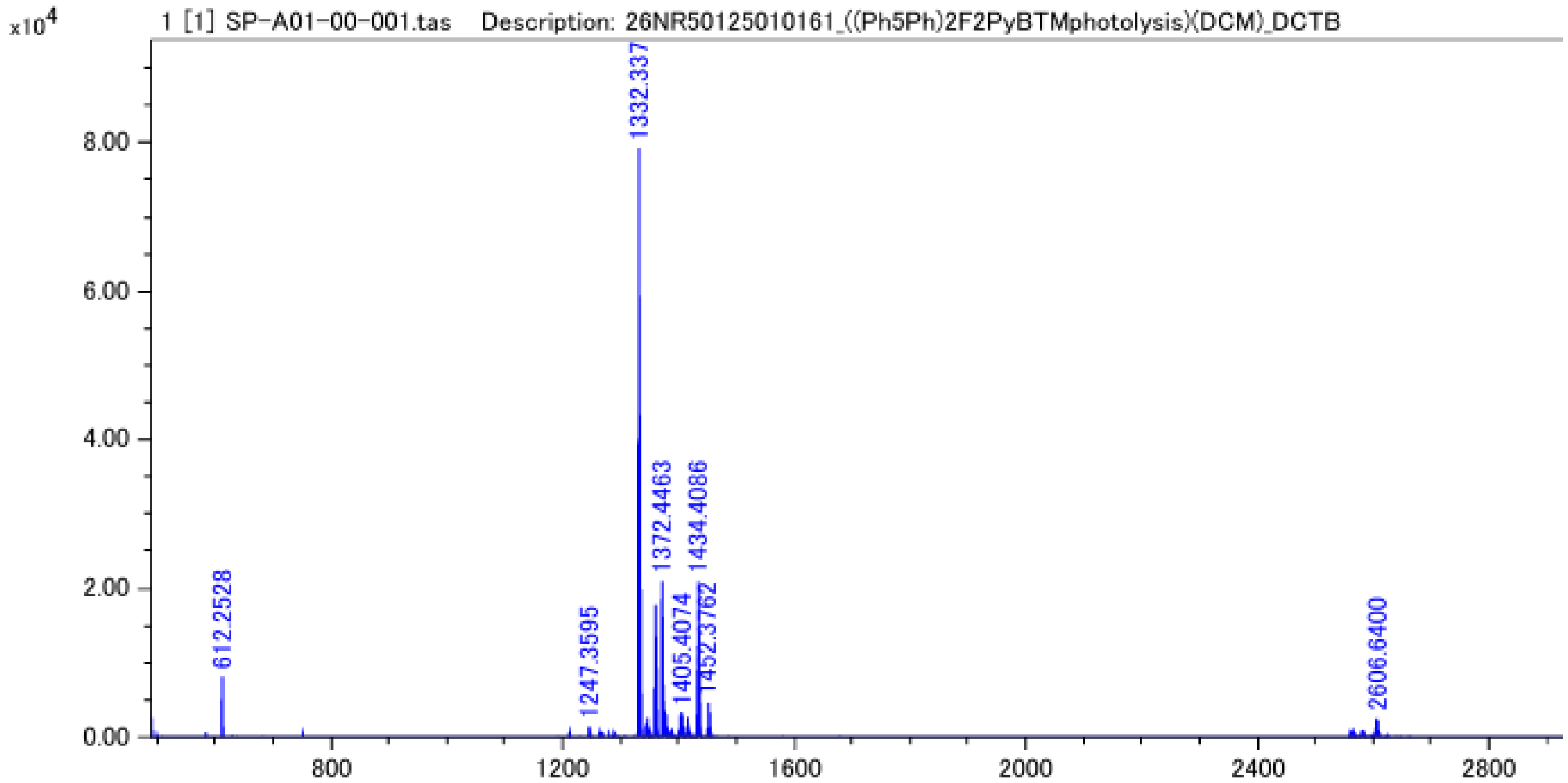

(b)

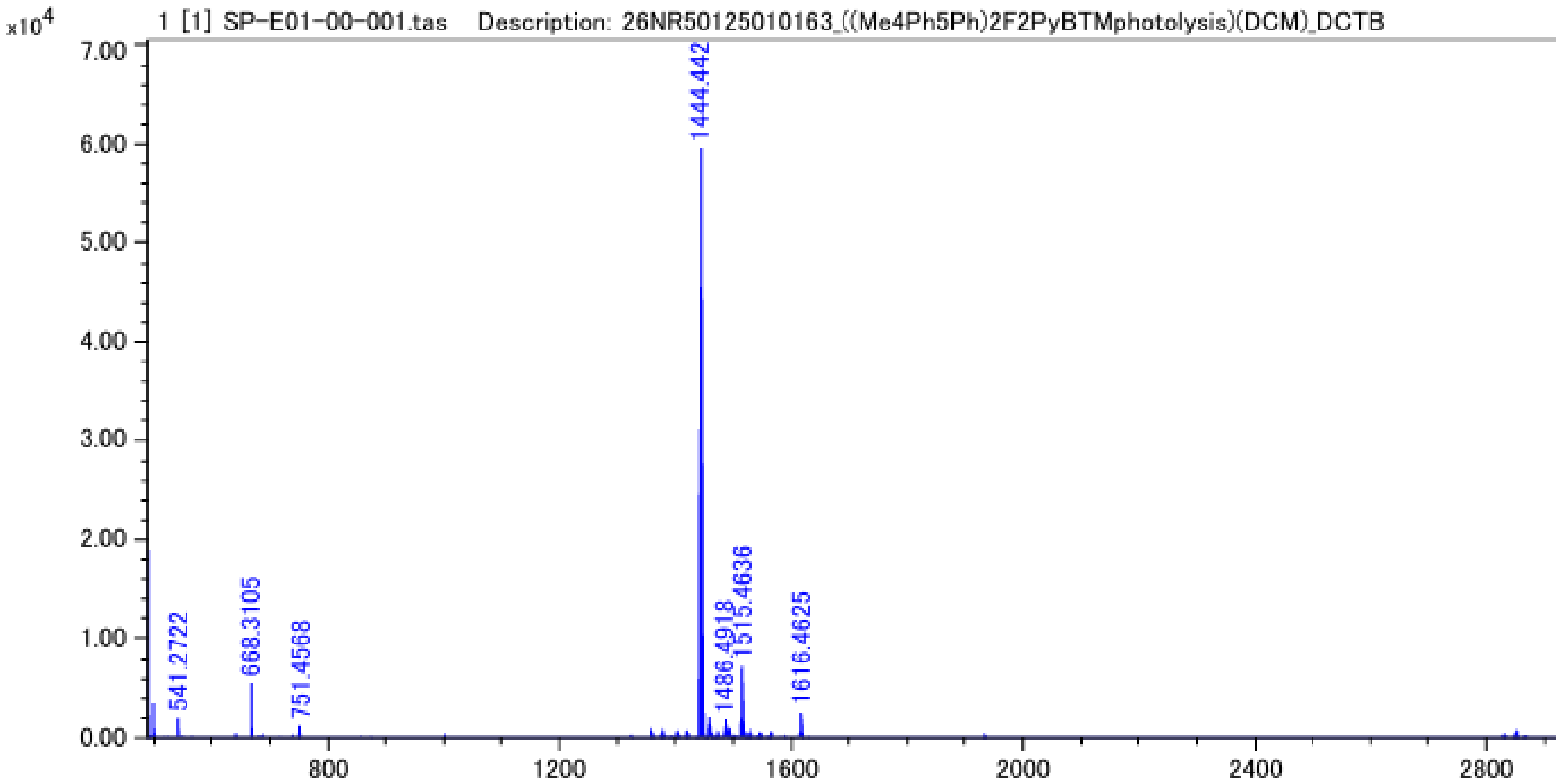

Fig. S13 HR-MALDI-TOF mass spectrum of (a) photodegraded $(Ph_5Ph)_2F_2PyBTM$ and (b) $(Me_4Ph_5Ph)_2F_2PyBTM$ prepared by 370-nm UV lamp irradiation in dichloromethane.

^{1}H NMR (400 MHz, $CDCl_3$) spectrum of αH-F_2PyBTM-[B(Epin)]$_2$.

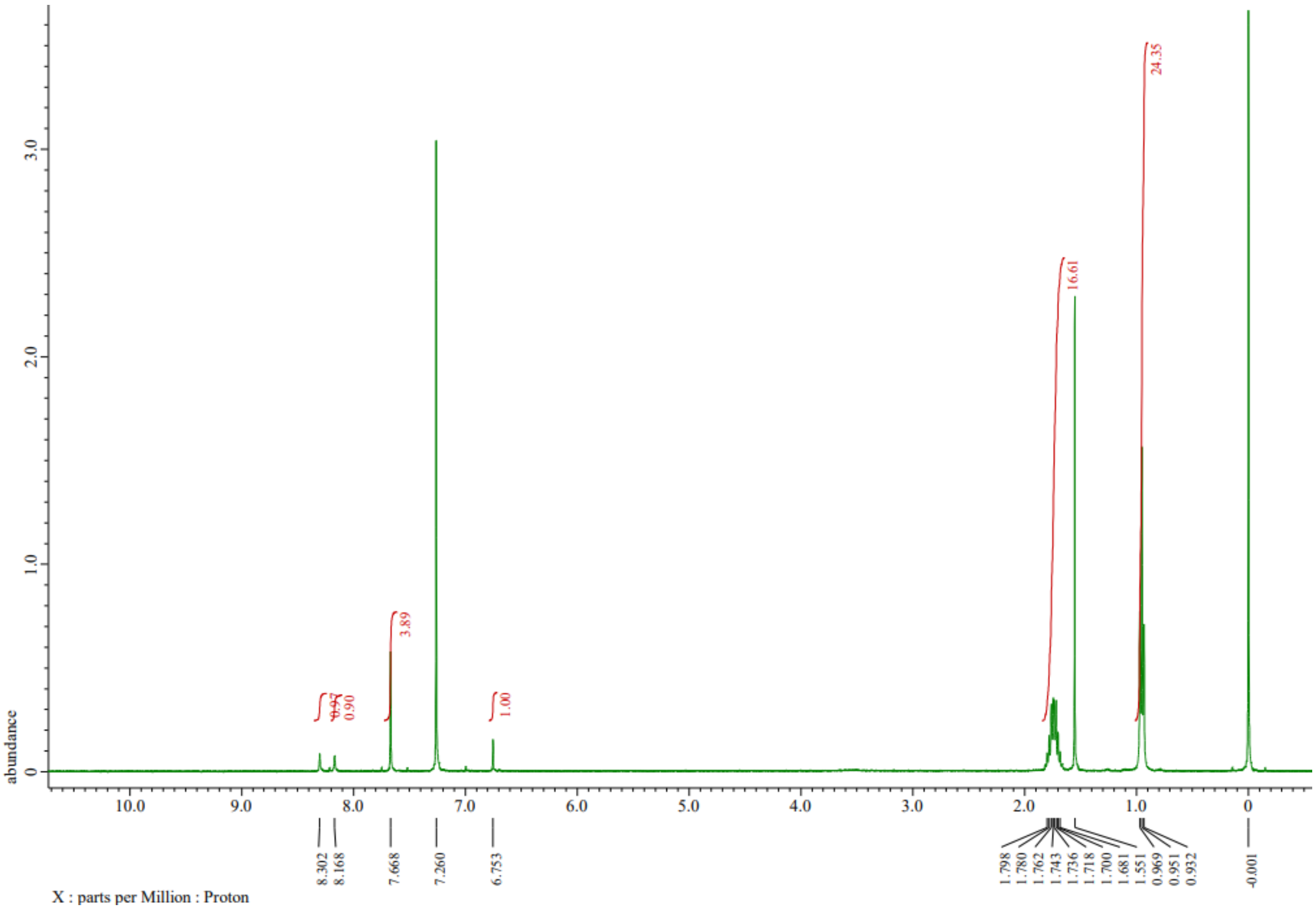

^{13}C NMR (101 MHz, $CDCl_3$) spectrum of αH-F_2PyBTM-[B(Epin)]$_2$.

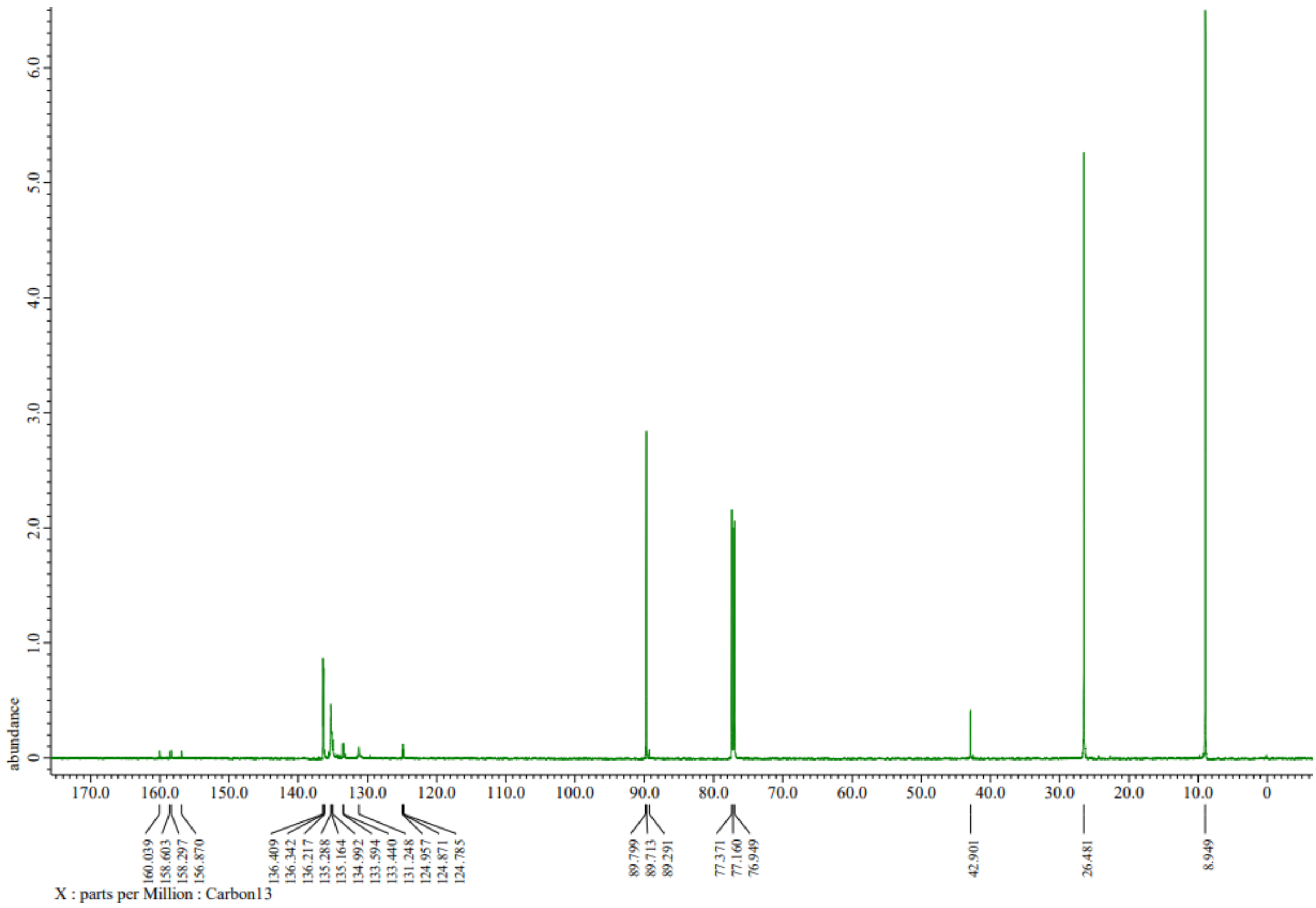

^{1}H NMR (400 MHz, $CDCl_3$) spectrum of αH-$(Me_4Ph_5Ph)_2F_2PyBTM$.

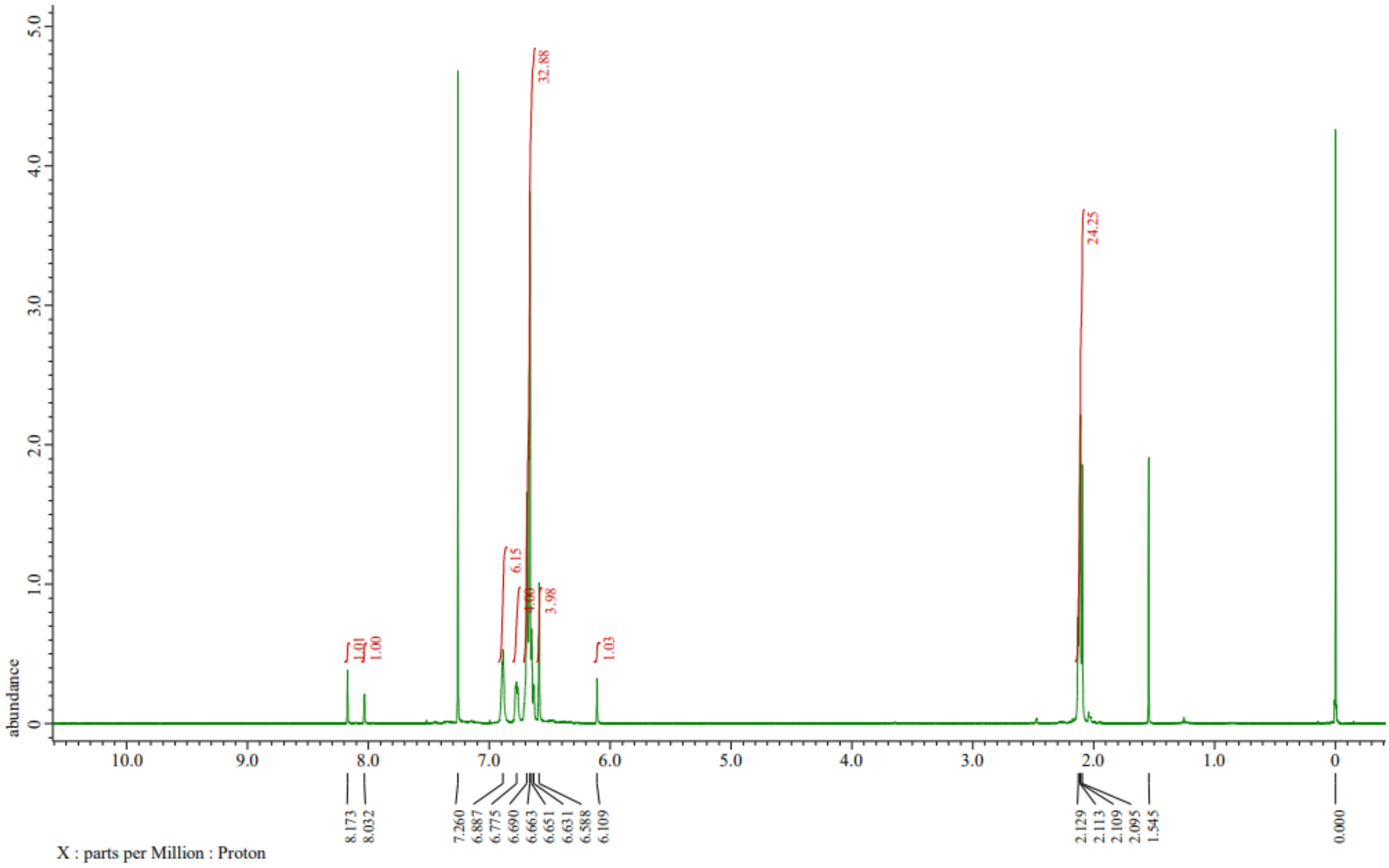

^{13}C NMR (151 MHz, $CDCl_3$) spectrum of αH-$(Me_4Ph_5Ph)_2F_2PyBTM$.

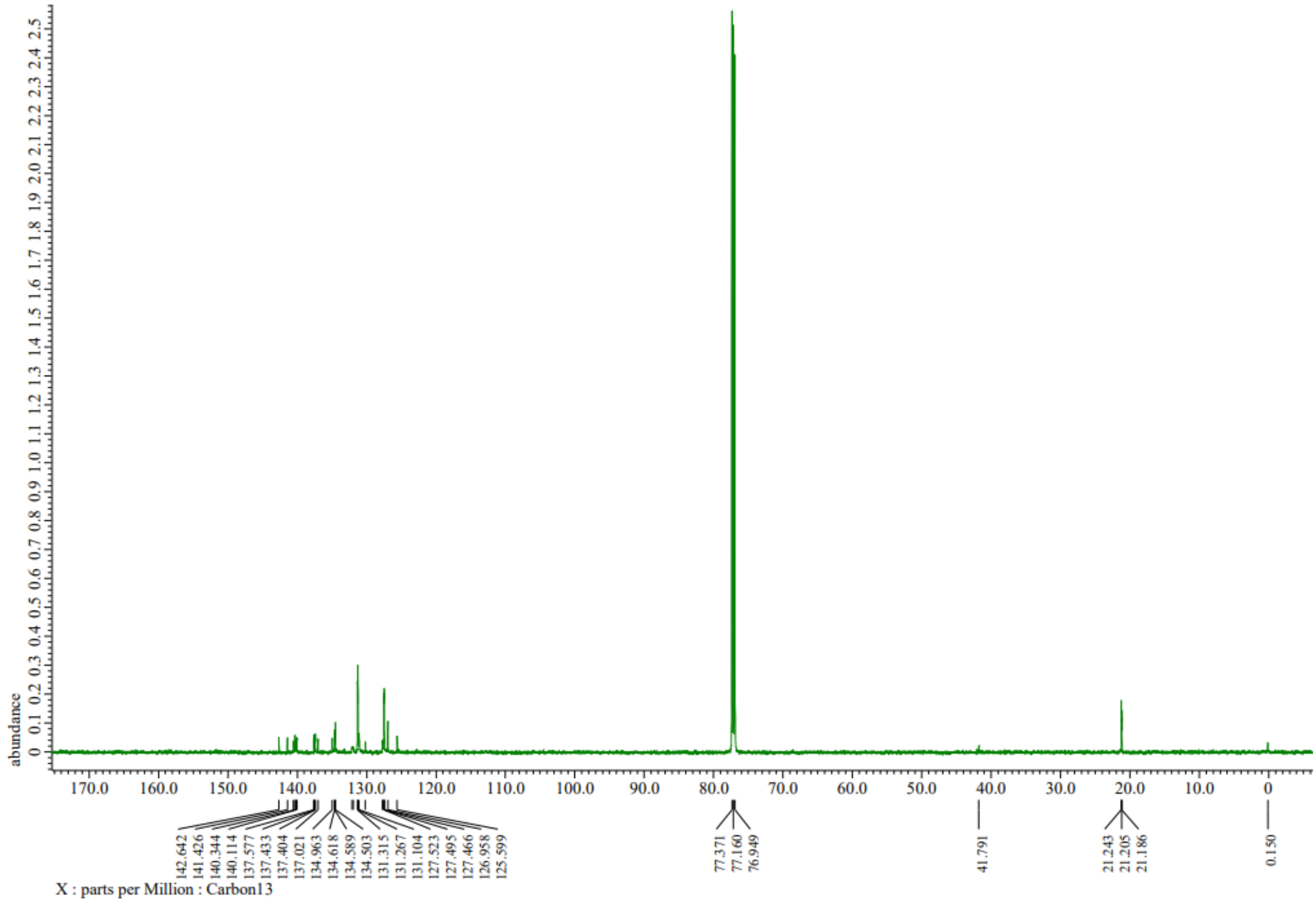

^{1}H NMR (600 MHz, $CDCl_3$) spectrum of *αH*-$(Ph_5Ph)_2F_2$PyBTM.

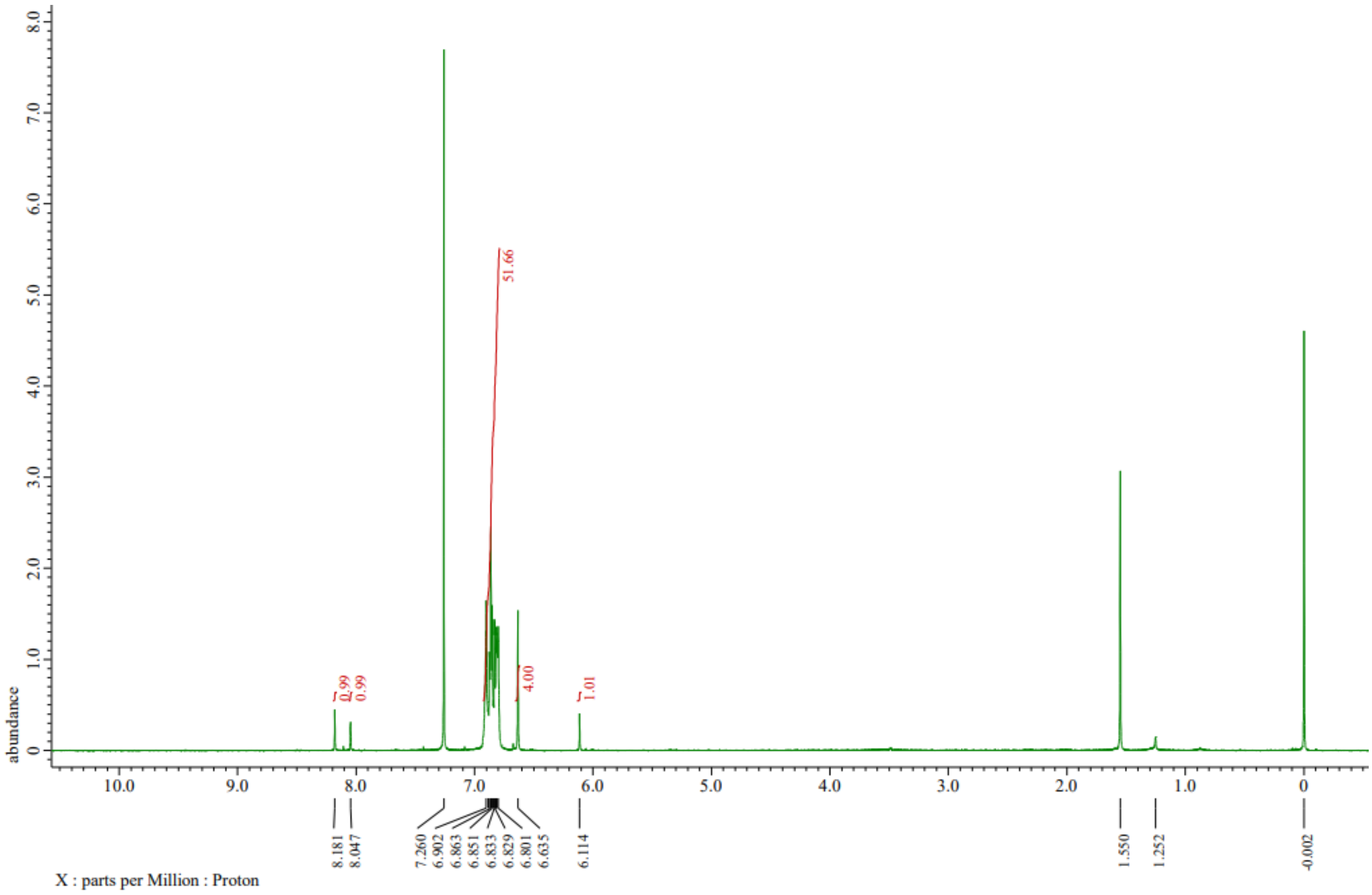

^{13}C NMR (151 MHz, $CDCl_3$) spectrum of *αH*-$(Ph_5Ph)_2F_2$PyBTM.

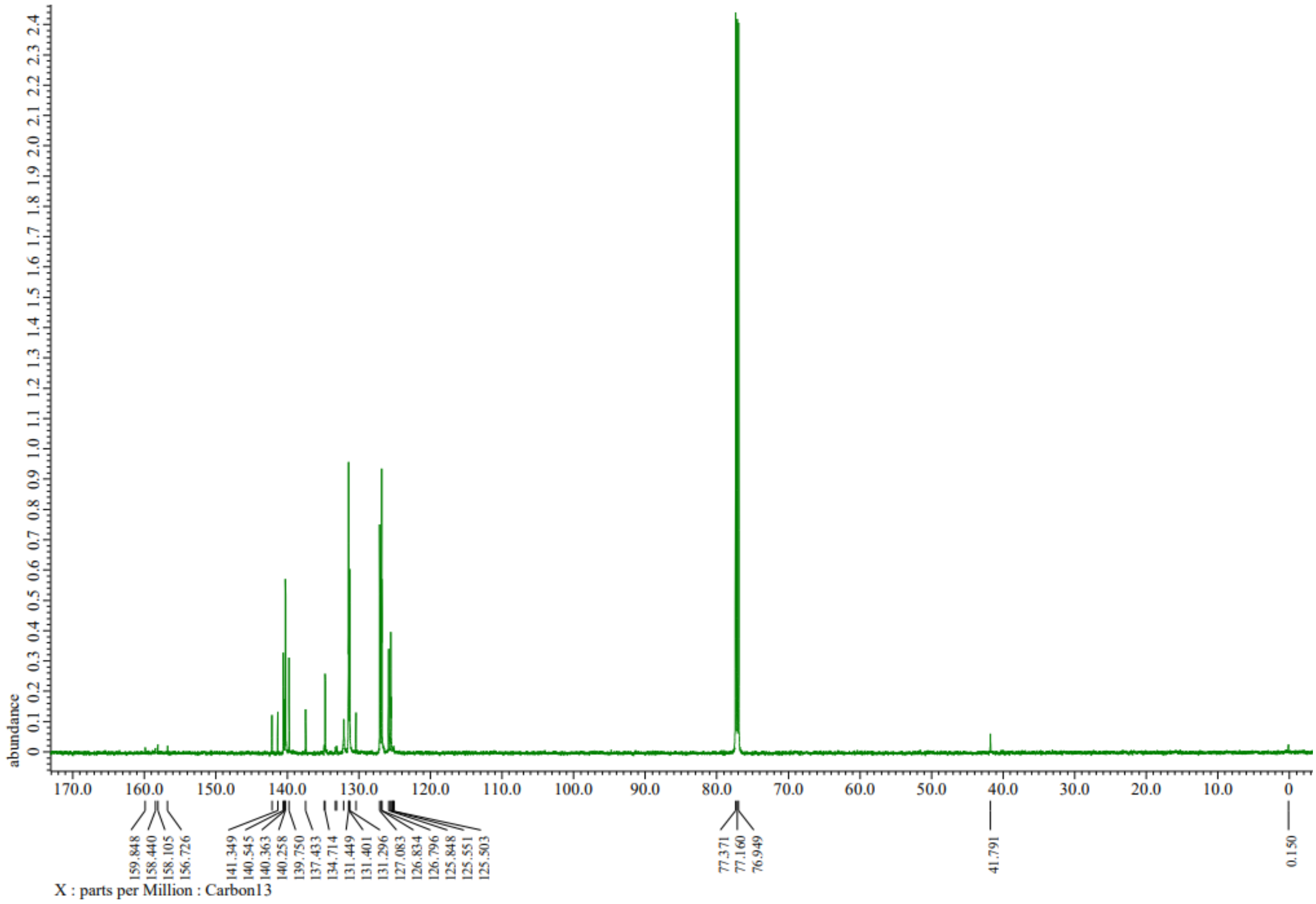

^{19}F NMR (470 MHz, $CDCl_3$) spectrum of αH-F_2PyBTM-[B(Epin)]$_2$.

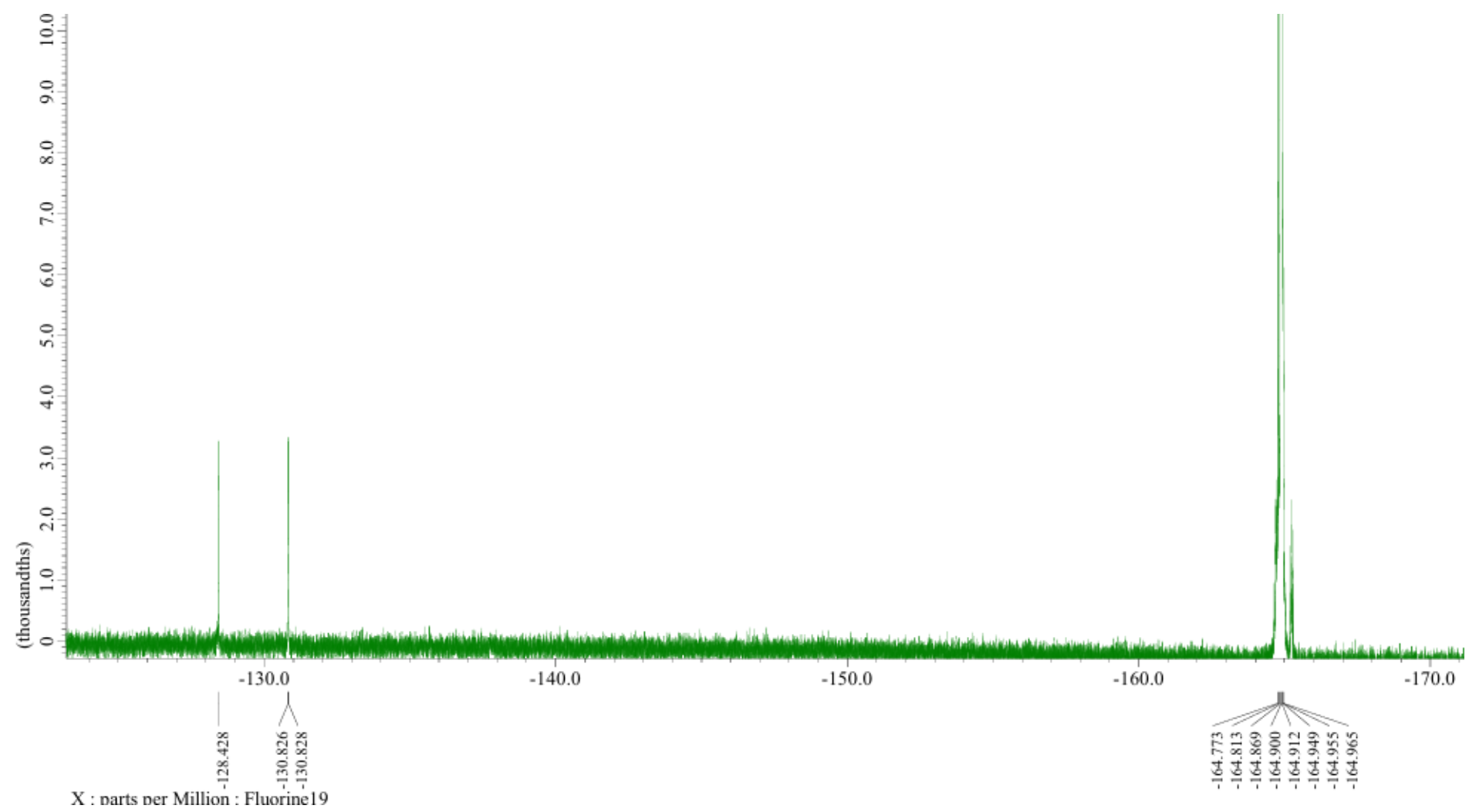

^{19}F NMR (470 MHz, $CDCl_3$) spectrum of αH-(Me_4Ph_5Ph)$_2F_2$PyBTM.

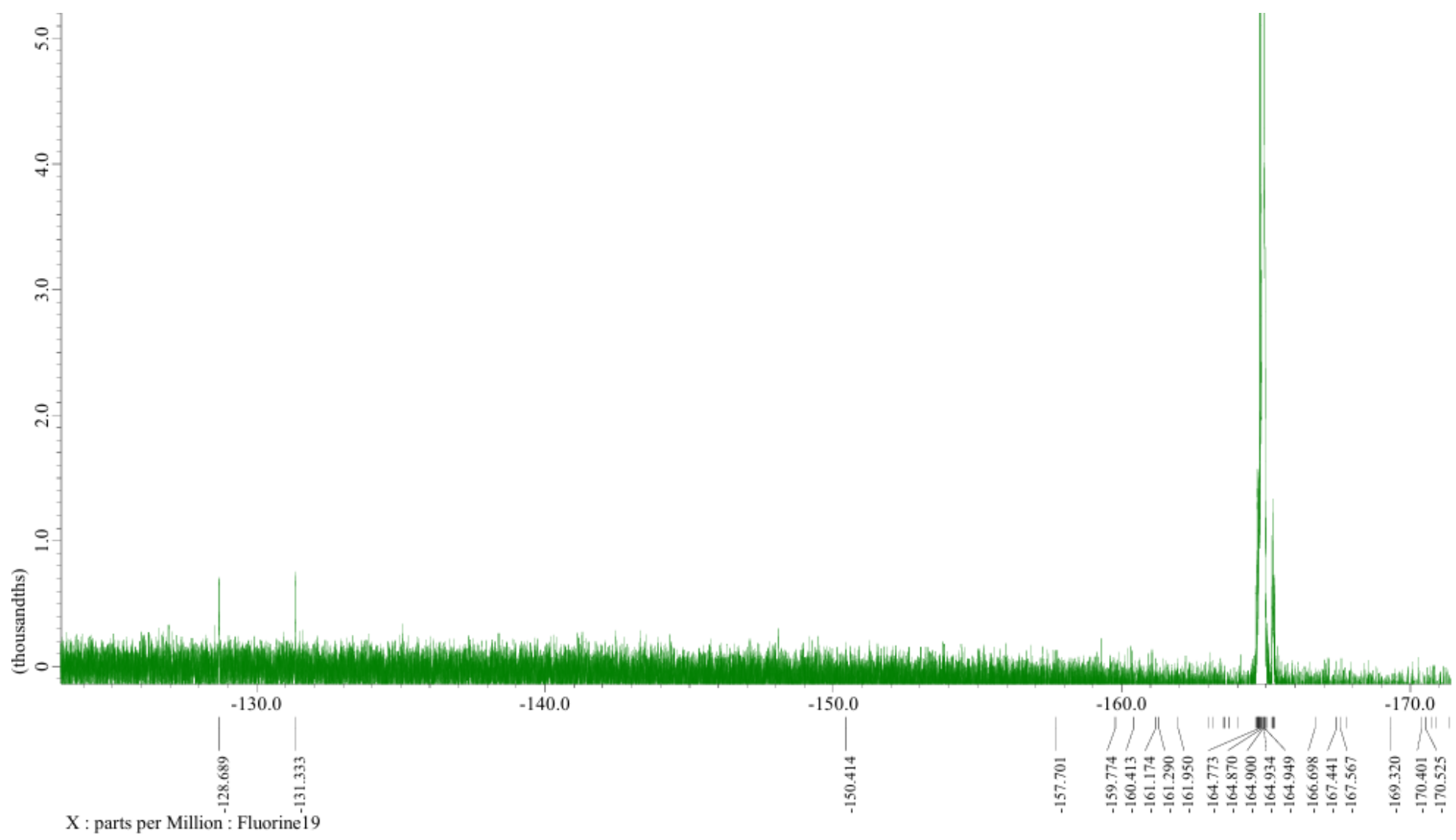

^{19}F NMR (470 MHz, $CDCl_3$) spectrum of αH-(Ph_5Ph)$_2F_2$PyBTM.

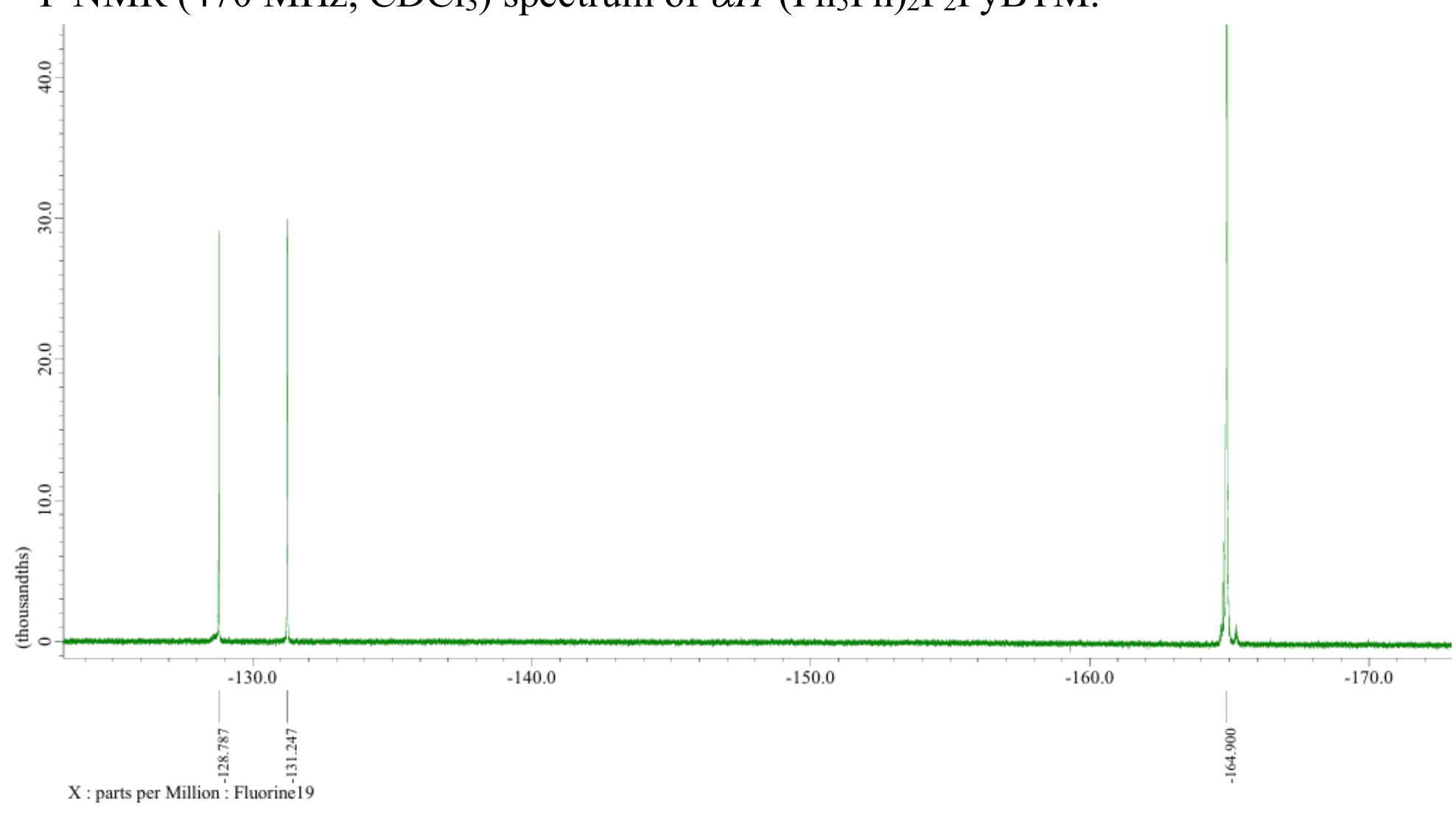

HR-MALDI-TOF mass spectrum of αH-F_2PyBTM-[B(Epin)]$_2$, full spectrum (bottom) and isotopic pattern of the molecular peak.

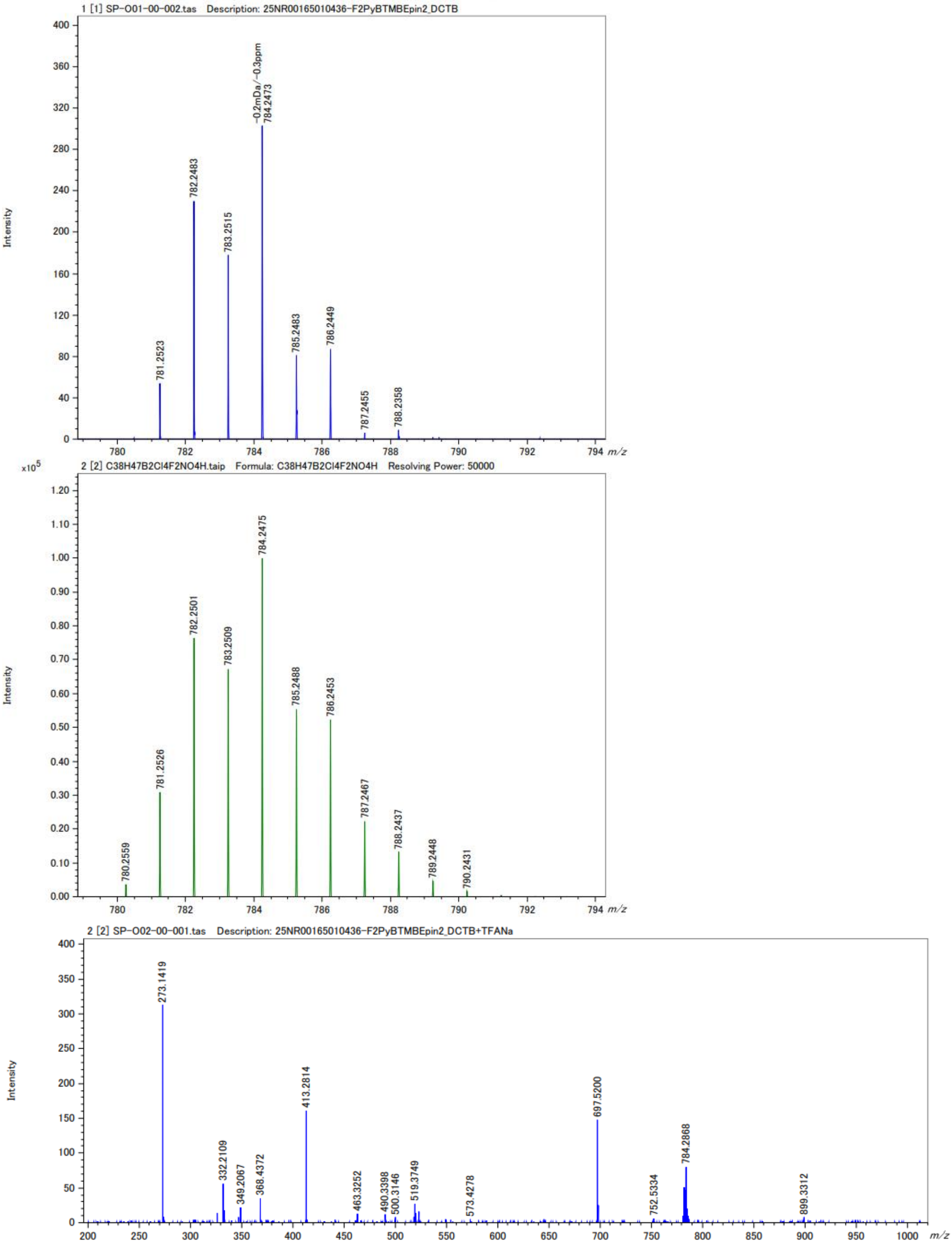

HR-MALDI-TOF mass spectrum of αH-(Me_4Ph_5Ph)$_2F_2$PyBTM, full spectrum (bottom) and isotopic pattern of the molecular peak.

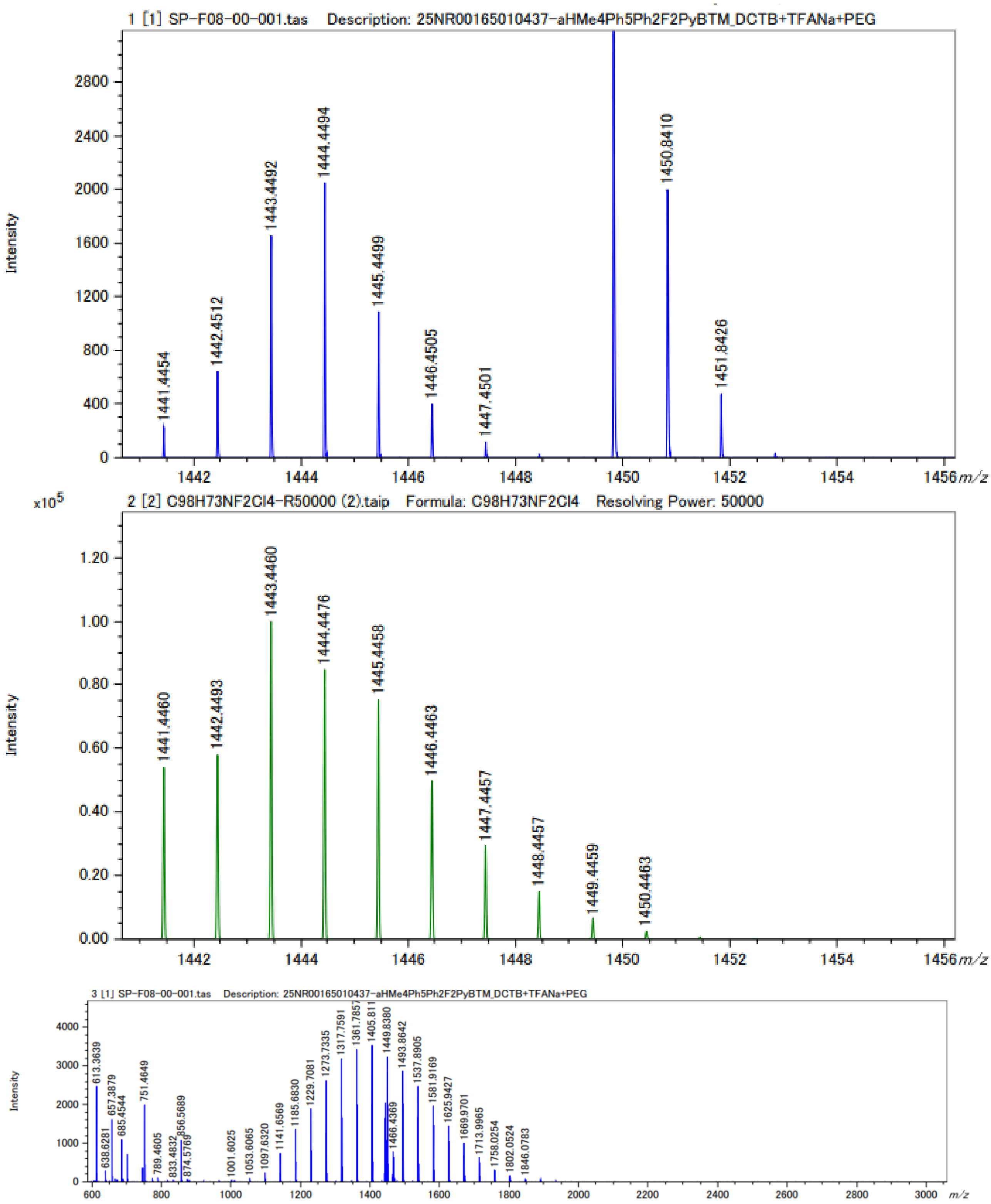

HR-MALDI-TOF mass spectrum of $(Me_4Ph_5Ph)_2F_2PyBTM$, full spectrum (bottom) and isotopic pattern of the molecular peak.

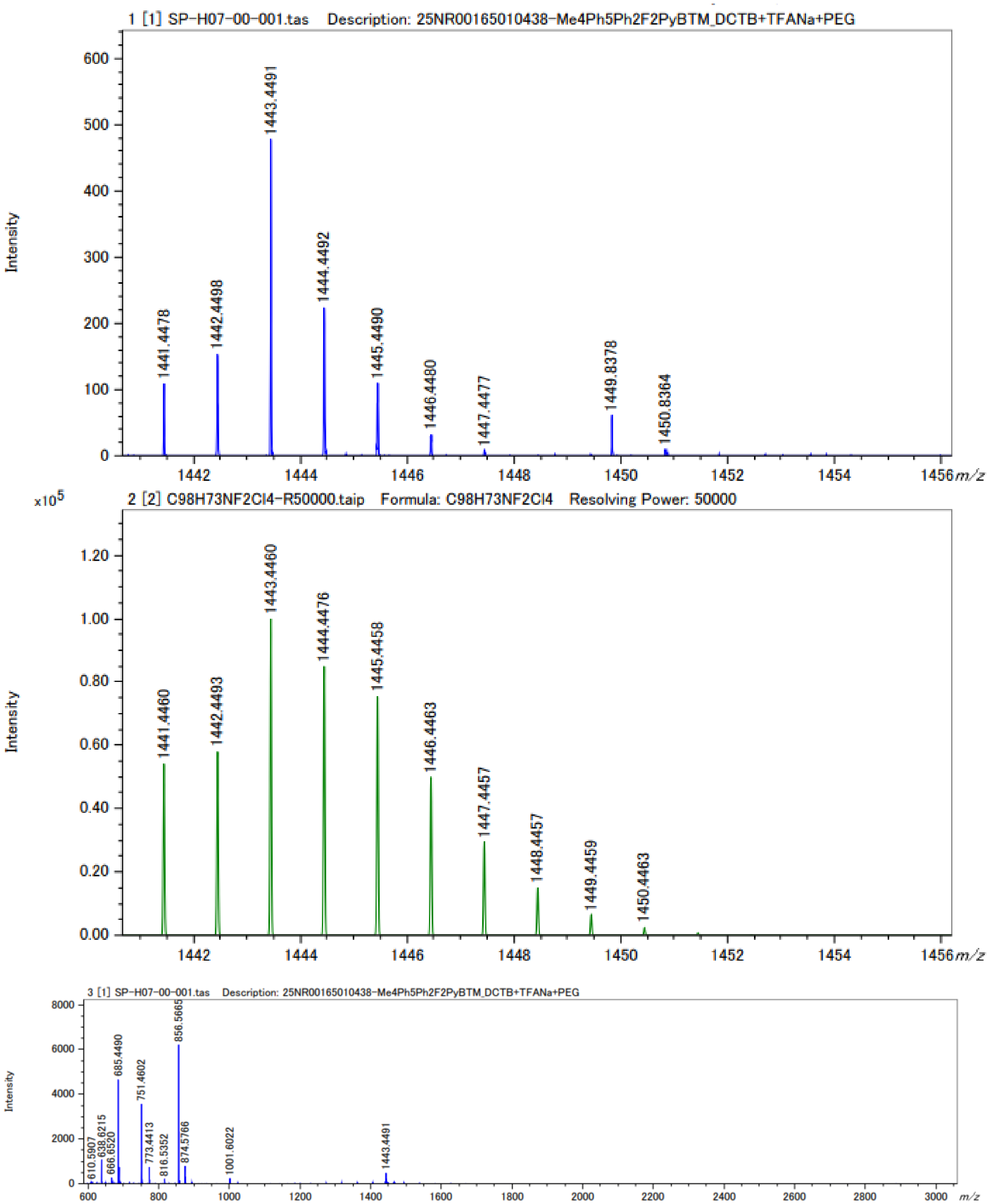

HR-MALDI-TOF mass spectrum of αH-$(Ph_5Ph)_2F_2$PyBTM, full spectrum (bottom) and isotopic pattern of the molecular peak.

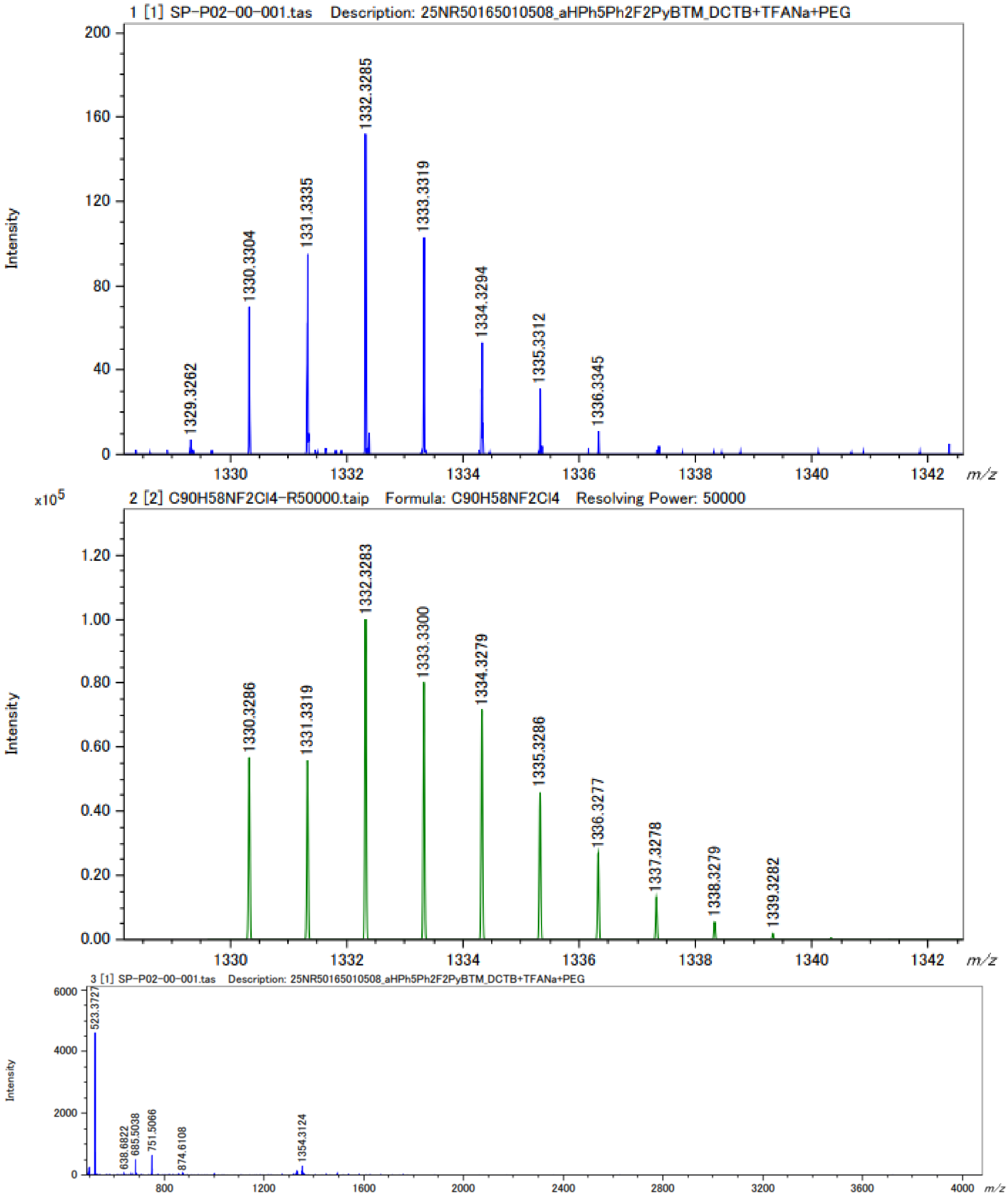

HR-MALDI-TOF mass spectrum of $(Ph_5Ph)_2F_2PyBTM$, full spectrum (bottom) and isotopic pattern of the molecular peak.

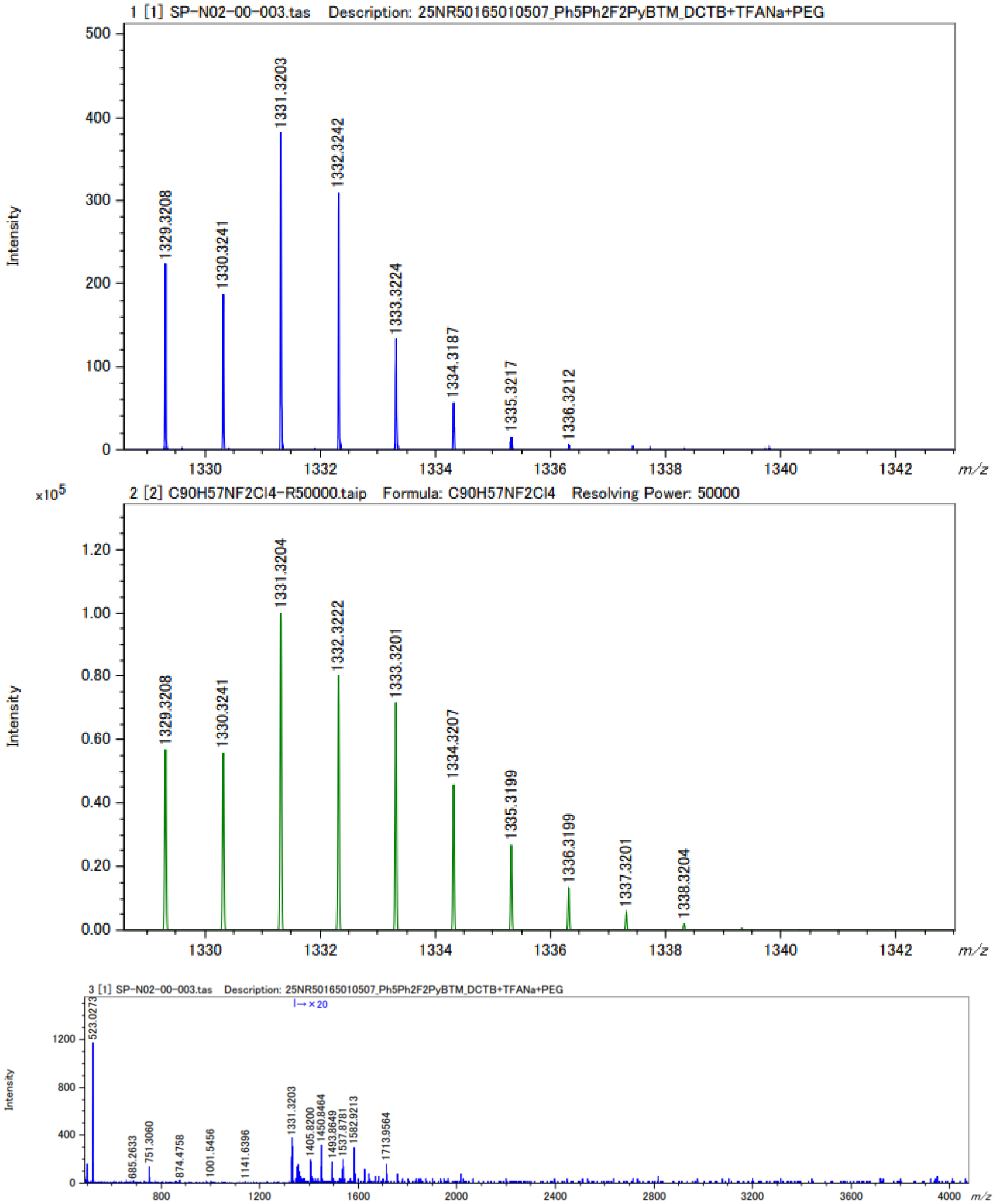

Supplement: Supplementary file 1 [file SC-017-D6SC05107C-s001.pdf]
